# Hydride-induced palladium self-diffusion and surface restructuring on Pd(111)

**DOI:** 10.1038/s41467-026-77704-9

**Published:** 2026-09-15

**Authors:** Raju Lipin, Muhammad Umer, Apinya Ngoipala, Sousa Javan Nikkhah, Matthias Vandichel

**Affiliations:** 1 Materials and Catalysis Modelling (MACATAMO) Group, Bernal Institute, University of Limerick, Limerick, Ireland; 2 Simulation of Structures across Scales (SUSAS) Group, Department of Chemistry, Maynooth University, Maynooth, County Kildare Ireland; 3 Hamilton Institute; Kathleen Lonsdale Institute, Maynooth University, Maynooth, County Kildare Ireland

**Keywords:** Energy, Electrocatalysis, Electrocatalysis, Electrocatalysis

## Abstract

Reaction conditions significantly affect the electrocatalyst surface but are often overlooked when evaluating catalytic performance. Pd forms hydride phases during proton reduction under cathodic potentials, which can lead to surface reconstructions and defect formation. However, the influence of hydride formation on Pd atom migration remains underexplored. Here, we show, using density functional theory and molecular dynamics simulations, the thermodynamic and hydride conditions associated with surface reconstruction in PdH_x_/Pd(111) systems during the initial stages of hydride formation. Surface Pourbaix diagrams show the predicted stability of PdH_x_/Pd phases under reaction conditions, while calculated Pd migration barriers decrease with hydride sublayers and become negligible at high hydrogen concentrations. Atomistic simulations further show that at least three subsurface hydride layers are necessary for observable reconstruction, dislocation and defect formation, during which Pd atoms predominantly migrate outward through strain relaxation while hydrogen atoms locally adjust to the reconstructed lattice. Overall, our results suggest an interplay between hydride formation and atom mobility, highlighting the potential importance of dynamic surface transformations when designing and evaluating Pd-based electroreduction catalysts.

## Abstract (web summary)

Palladium catalysts absorb hydrogen under reducing conditions, but the resulting surface changes remain poorly understood. Here, the authors show that hydride formation in palladium drives atom migration and surface reconstruction, creating defects that may influence catalytic activity.

## Introduction

Palladium (Pd)-based nanomaterials play a pivotal role in energy-related applications, including hydrogen storage and catalysis^1–5^. Upon interaction with hydrogen, Pd undergoes hydriding, a process that induces structural transformations and alters its physicochemical properties. Under ambient conditions, Pd forms a solid solution with hydrogen (α-phase), which evolves into a hydride phase (β-phase) at increasing hydrogen (H) concentrations. This phase transition is accompanied not only by a substantial volumetric expansion (~10%) that generates residual stresses and lattice distortions, but also by pronounced changes in the electronic structure and vibrational properties of the material, such that Pd and PdH_x_ exhibit distinct physicochemical characteristics^6–10^. Several experimental and theoretical studies have investigated the effects of hydriding on the structure of Pd, wherein the introduction of hydrogen generates surface and bulk defects, including vacancies, dislocations, and strain-induced reconstructions^11–19^. Thompson et al. showed that defect formation in Pd depends strongly on grain size, with nanocrystalline Pd favoring vacancy-like defects, while coarse-grained Pd exhibits significant dislocation densities^20^. Similarly, Ulvestad et al. used coherent X-ray diffraction imaging to reveal strain-induced reconstructions in Pd nanocubes during hydriding^21^. Our recent molecular dynamics (MD) simulations and electrochemical studies have also demonstrated that hydriding induces surface roughening and morphological evolution in Pd catalysts^22^. Beyond their traditional role in hydrogen storage, hydride phases are increasingly being recognized as catalytically relevant species under aqueous and electrochemical conditions^23–25^. For example, Xia and Wang demonstrated interfacial water dissociation at Pd-supported catalysts can generate Pd hydrides that act as a new catalytically active phase at the solid–liquid interface, directly participating in catalytic turnover rather than merely serving as hydrogen reservoirs^26^. Such findings suggest that hydride formation can fundamentally alter both the catalytic and structural state of Pd under operating conditions. Consequently, understanding how hydriding influences not only reactivity but also atomic mobility, defect evolution, and surface reconstruction is essential for establishing realistic structure–property relationships in Pd-based catalysts.

Beyond Pd systems, hydrogen-induced surface reconstruction under electrocatalytic conditions has been investigated in other transition metals using advanced theoretical approaches. For example, grand-canonical DFT (GC-DFT) combined with global optimization strategies has revealed hydrogen-driven restructuring phenomena on Cu surfaces under reducing potentials^27,28^. These studies demonstrate that adsorbate-induced stabilization of metastable surface motifs can significantly alter catalytic behavior. More recently, Alexandrova and co-workers developed a quasi-kinetic Monte Carlo framework capable of capturing potential-dependent surface evolution beyond equilibrium Boltzmann statistics, explicitly accounting for kinetic trapping under reducing and elevated-temperature conditions^29,30^. Collectively, these advances underscore the dynamic nature of catalyst surfaces under reaction environments and emphasize the need for multiscale approaches that couple thermodynamics with atomic-scale kinetics. In particular, defects generated during hydriding are expected to play a critical role in influencing hydrogen absorption, adsorption, and diffusion kinetics in metal electrocatalysts^31,32^.

While numerous studies have examined hydrogen absorption and diffusion in Pd (surfaces, slabs and nanoparticles)^33–47^, and Pd-self diffusion in bulk Pd-PdH_x_ systems^48^, considerably less attention has been paid to Pd atom mobility at Pd surfaces during the initial stages of hydride formation, where local hydrogen accumulation, strain, and surface reconstruction are strongly coupled^14^. Most existing studies on Pd self-diffusion have focused on pure Pd surfaces or adatom diffusion, often neglecting the role of hydriding. For instance, Beszeda et al. examined Pd self-diffusion on pristine surfaces^49^, while Zhang et al. showed that single vacancies are more stable in the first layer, allowing voids in the second layer to migrate to the first layer on Pd(111) and Pd(001) surfaces^50,51^. Similarly, some computational studies employing the MD simulation method with semi-empirical potentials, such as modified analytical embedded atom methods (MAEAM), have focused on the migration of vacancies and self-diffusion of Pd atoms (both single and clusters) on pure Pd surfaces^50–54^. In another set of investigations, Jun et al. computed the activation barriers corresponding to various single-atom diffusion pathways on different fcc metal surfaces using the action-derived MD (ADMD) method^55,56^. A separate MD study found that the surface diffusion of Pd adatoms on a pristine Pd(110) was faster than on a defective Pd(110)-(1 × 2) surface^57^. Hydrogen–vacancy interactions, including hydrogen trapping and hydrogen-assisted vacancy stabilization, are also well established in bulk metals, grain boundaries and Pd-based membrane systems^58,59^. However, these studies do not directly quantify how progressive subsurface hydride formation modifies Pd migration pathways and surface restructuring at PdH_x_/Pd(111) interfaces. Consequently, Pd self-diffusion at such interfaces, where hydride-induced strain and surface instabilities coexist, remains largely unquantified, despite its potential implications for catalyst stability and performance.

Herein, we employ a combination of DFT and MD simulations with atomic cluster expansion potentials trained on a PBE-D3 dataset (ACE-PBE-D3)^22,60^ to investigate Pd-migration in PdH_x_/Pd(111) systems. Our DFT calculations suggest Pd migration barriers decrease as more and deeper subsurface layers are being occupied by hydrogen atoms. Furthermore, our MD simulations indicate that, within the models considered, at least three subsurface hydride layers are associated with facilitating the movement of Pd atoms from the PdH_x_ region to the surface. Together, these results provide insights into the possible role of hydriding in Pd mobility and associated strain-induced surface reconstruction, phenomena widely observed in material science and particularly relevant in Pd catalysis and electrocatalysis.

## Results

### DFT insights: hydride layering & Pd mobility

**Fig. 1:**
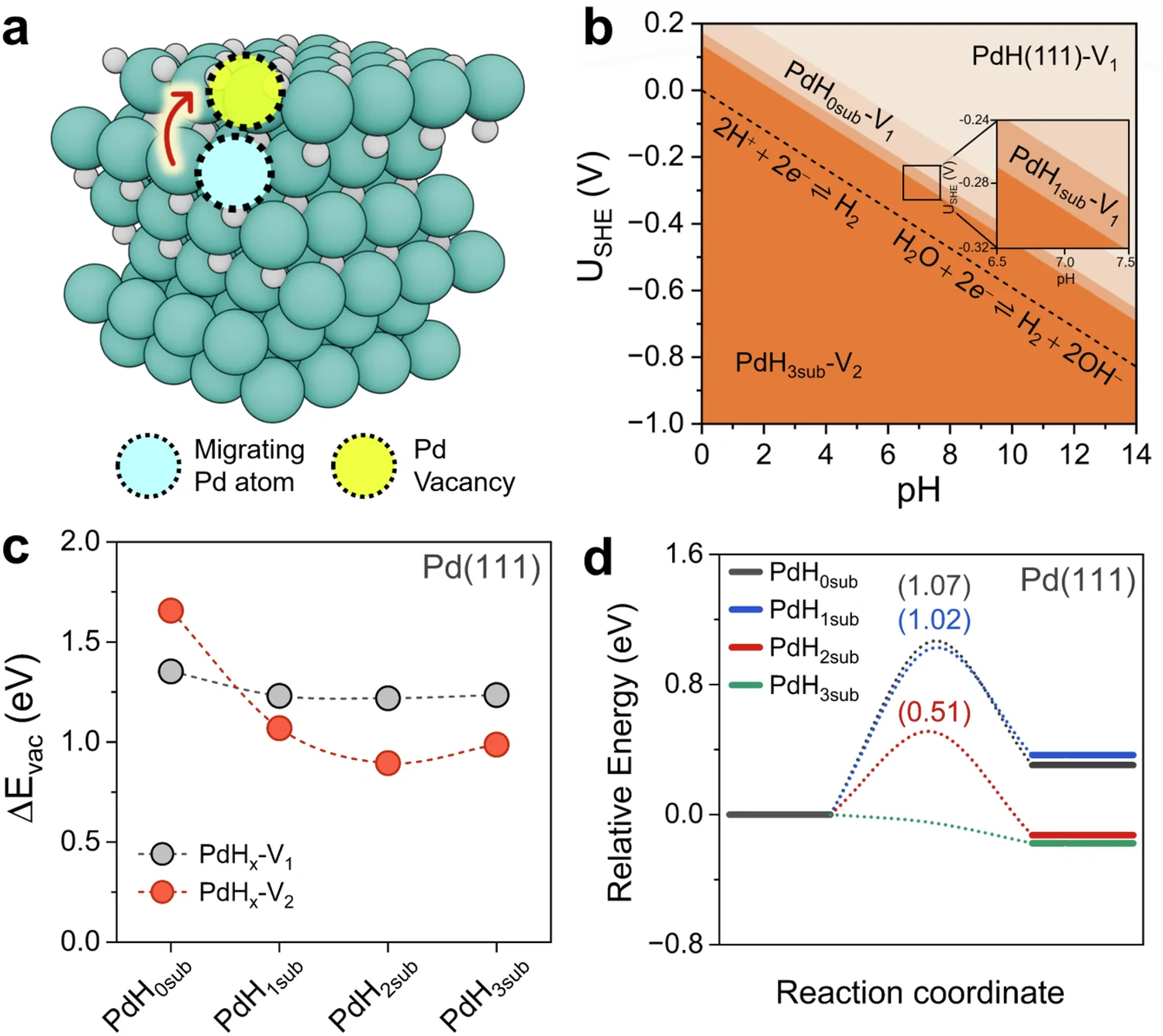
Subsurface hydrogen promotes vacancy formation and Pd migration under reducing conditions. **a** Perspective views of 1.00 ML H-covered PdH_x_/Pd(111) surfaces with single Pd-vacancy sites. **b** Surface Pourbaix diagrams for such surfaces, showing their electrochemical stability regions. The black dashed lines represent the hydrogen reduction regime as a function of pH. Here, U_SHE_ is the electrode potential relative to the standard hydrogen electrode at 298.15 K and 1 bar. **c** Vacancy formation energy (ΔE_vac_) for 1.00 ML H-covered Pd(111) surfaces with varying hydrogen subsurface concentrations (PdH_0sub_, PdH_1sub_, PdH_2sub_, PdH_3sub_). **d** Reaction energy profiles for Pd migration on Pd(111) surfaces, showing the relative energies of the initial and final states, along with the corresponding migration barriers for different hydrogen subsurface concentrations (PdH_0sub_, PdH_1sub_, PdH_2sub_, PdH_3sub_). Relative energy is the energy of the final state relative to the initial state. All structures were relaxed to allow in-plane stress relaxation while keeping the cell shape and volume fixed. The results, obtained using the PBE functional with the D3(BJ) dispersion correction, show the ease of vacancy formation in the sublayer as a function of the number of hydride sublayers and their stability under reaction conditions. Source data are provided as a Source Data file.

A Pd vacancy in the first sublayer of a Pd(111) system is shown to be energetically more stable than on the surface, particularly with increasing hydrogens in the sublayers^61^. In line with this, we extend our understanding of PdH_x_/Pd(111) surfaces by evaluating their thermodynamic stability under electrochemical conditions through the construction of surface Pourbaix diagrams (SPDs) for defective PdH_x_/Pd(111) systems with varying hydrogen sublayers and both surface (V_L1_) and subsurface (V_L2_) vacancies (Fig. 1a). In general, at increasingly cathodic potentials, defective Pd(111), like any Pd surface, tends to undergo hydriding, with hydrogen atoms occupying interstitial sites in the sublayers (Fig. 1b). Interestingly, PdH_3sub_/Pd(111) (with three hydride sublayers) is thermodynamically more stable when the vacancy is in the first sublayer (V_L2_). In contrast, for systems with fewer hydrogen sublayers, vacancies tend to be more stable at the surface^61^. From the SPD analysis, even at 298.15 K under thermocatalytic conditions (U_SHE_ = 0 V), the formation of PdH_x_/Pd(111) is highly favorable, emphasizing the importance of considering hydride layers in computational investigations, particularly when describing catalytic processes. The stability of defective PdH_x_/Pd(hkl) surfaces is further quantified by vacancy formation energies (see supporting information for details). The data indicate that in Pd(111), subsurface vacancy formation becomes increasingly favorable with additional hydrogen sublayers (Fig. 1c). The effect of hydriding into the sublayers is further highlighted in the evolution of areal strain and surface energy (Fig. S1) (see supporting information for details). The areal strain increases with higher hydrogen concentrations, indicative of lattice expansion due to hydrogen uptake. Concurrently, the surface energy decreases steadily, suggesting that hydriding in the sublayers thermodynamically stabilizes the defective surfaces. Notably, these trends are consistent across both V_L1_ and V_L2_ models, although a slightly greater stabilization is observed for V_L2_ at higher hydrogen concentrations, emphasizing the role of subsurface vacancy in modulating hydrogen accommodation and surface energetics. Importantly, since the hydrogen-rich PdH_x_ states investigated in the subsequent DFT and MD analyses are thermodynamically stable within this cathodic potential regime, Pd self-diffusion and surface reconstruction should be interpreted as occurring under reducing conditions where sufficient subsurface hydrogen accumulation is achieved. In this sense, the applied potential indirectly governs Pd mobility through control of the hydrogen chemical potential and hydride loading, rather than directly modifying the migration barrier.

**Fig. 2:**
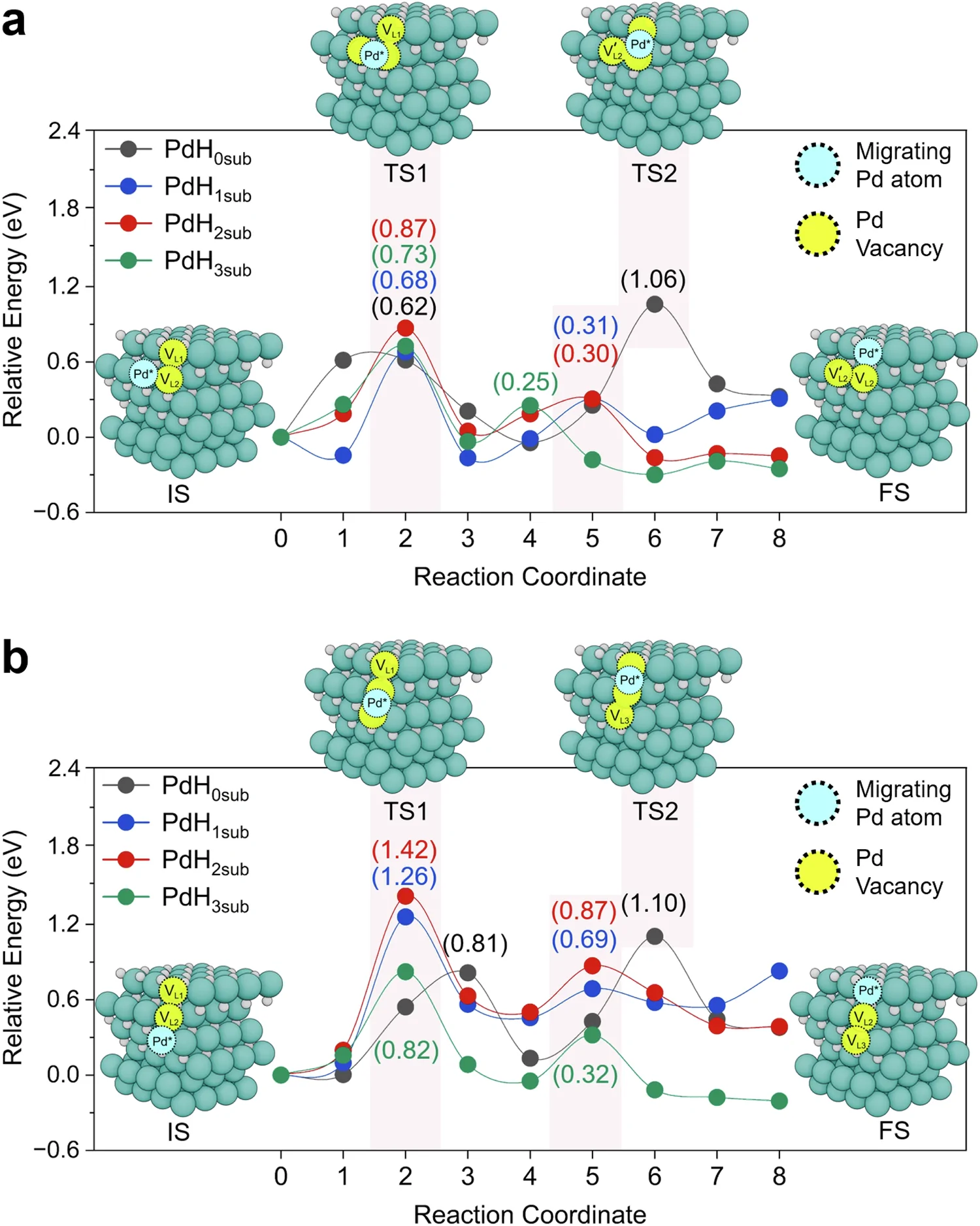
Subsurface hydrogen lowers the barriers for Pd migration. Reaction energy profiles for Pd migration on Pd(111) surfaces with a double-vacancy configuration calculated using the PBE functional, including the D3(BJ) dispersion correction. The profiles show the relative energies of the initial, intermediate, and final states, along with the corresponding migration barriers for different subsurface hydrogen concentrations (PdH_0sub_, PdH_1sub_, PdH_2sub_, PdH_3sub_). Two distinct pathways are considered: **a** Route 1 involves lateral migration within the second layer (L2) followed by upward diffusion into the surface vacancy (L1), while **b** Route 2 proceeds via a deeper-layer exchange mechanism (L3 → L2 → L1). The first migration process is rate-determining (the highest barrier), and the energy levels relative to the starting position are indicated. Source data are provided as a Source Data file.

To gain insights into Pd atom mobility, we examined the migration barrier associated with Pd movement from the first sublayer to a surface vacancy (Fig. 1a). To assess the impact of hydriding on Pd migration, we considered multiple PdH_x_/Pd(111) systems with varying hydrogen concentrations: (1) 1.00 ML of adsorbed hydrogen (H*), and (2) 1.00 ML H* with n hydrogen sublayers (*n* = 1, 2, 3). In general, we observe a stabilization trend where the product state (sublayer vacancy) becomes more stable than the reactant state (surface vacancy) with increasing sublayer hydriding. It is important to note that, in line with common practice for solid-state diffusion studies, this analysis is based solely on DFT-calculated energy differences between the initial and saddle point (∆E) and does not account for vibrational and entropic contributions used in the Pourbaix diagram construction. This discrepancy leads to some systems displaying a reverse trend when free energy corrections are considered. For all PdH_x_/Pd(111) model systems, the increased ease of Pd migration appears to be attributed with strain relaxation, which increases the distance between the migrating Pd atom and its nearest neighbors and thereby facilitates atomic movement (Fig. 1d). Interestingly, the calculated migration barriers decrease from model systems without hydride sublayers to those with a single hydride sublayer and become effectively barrierless with further subsurface hydriding, suggesting that Pd migration may be accessible at 298.15 K. To further evaluate the influence of the local vacancies on Pd self-diffusion, we extended our analysis to configurations containing two vacancies in the Pd(111) slab at different relative positions (Fig. 2). This allows assessment of how neighboring defects modulate migration energetics compared to the isolated single-vacancy case. Two distinct diffusion pathways were examined. In Route 1, a Pd atom from the second layer (L2) first occupies the adjacent L2 vacancy (${{\rm{V}}}_{{\rm{L}}2}^{{\prime} }$) through a lateral in-layer hop, followed by an upward migration into the surface vacancy (V_L1_). In Route 2, a Pd atom from the third layer (L3) first fills the V_L2_, after which the atom migrates upward into the surface vacancy. Thus, Route 1 proceeds via lateral rearrangement before vertical migration, whereas Route 2 involves a deeper-layer exchange mechanism. The calculated energetics indicate that, although the detailed barriers differ between the two routes, the qualitative trend remains consistent with the single-vacancy model: increasing subsurface hydrogen loading lowers the migration barrier and stabilizes the reconstructed configuration. Importantly, this overall trend is reproduced both with and without the D3-BJ dispersion correction. While the relative energetics at lower hydride loadings show some dependence on the treatment of dispersion correction, both approaches converge on the same key result at three hydride sublayers, where Pd migration becomes effectively lower or negligible. The corresponding calculations without the D3-BJ correction are provided in Figs. S20 and S21. In other words, hydride formation could facilitate Pd self-diffusion irrespective of whether the vacancy is isolated or part of a defect cluster. This suggests that hydrogen-rich environments promote defect-assisted surface restructuring even in more complex vacancy landscapes. In addition, we evaluated the work functions (Φ) of representative slab configurations along the vacancy-mediated diffusion coordinate to provide an approximate electrostatic reference for the potential window associated with Pd migration. The calculated work functions vary only slightly along the migration pathway, indicating that Pd self-diffusion proceeds without substantial changes in the electronic structure. In the single-vacancy models, subsurface hydrogen loading reduces the Φ difference between the initial (surface vacancy) and final (subsurface vacancy) states, consistent with hydrogen stabilizing reconstructed configurations (Table S1). While these DFT calculations provide initial insights into the influence of hydride layers on Pd mobility, they only represent static, zero-temperature approximations and are restricted to relatively small supercells. As such, they do not capture the dynamical evolution of the surface over time, long-range strain redistribution, or the collective atomic rearrangements that accompany surface reconstruction during hydride-induced lattice relaxation. To bridge this gap, we employed ACE-PBE-D3-based MD simulations to explicitly track the time-dependent structural evolution of hydrided Pd surfaces at a given temperature. This approach enables the exploration of strain relaxation mechanisms, defect formation, and collective atomic motion under varying hydrogen loadings. Given that the Pd(111) facet is the most stable and predominantly exposed surface in Pd nanostructure-based catalysts, we focused our MD investigations on PdHₓ/Pd(111) systems with systematically varied subsurface hydride layers.

### MD simulations insights: hydride-induced Pd mobility

While our earlier work provided insights into the role of hydrides in structural reconstruction, it did not quantify their impact on the self-diffusion of Pd atoms^22^. Here, we address this gap by investigating Pd(111) systems with varying hydride layers—ranging from one to four and even nine hydrogen sublayers—to mimic the effect of hydrogen loading on thermodynamically stable systems, as observed in Surface Pourbaix diagrams under cathodic conditions^61,62^. Nevertheless, the size of the model systems used in our earlier studies is too small to relax the accumulated strain, necessitating the use of larger systems. 

**Fig. 3:**
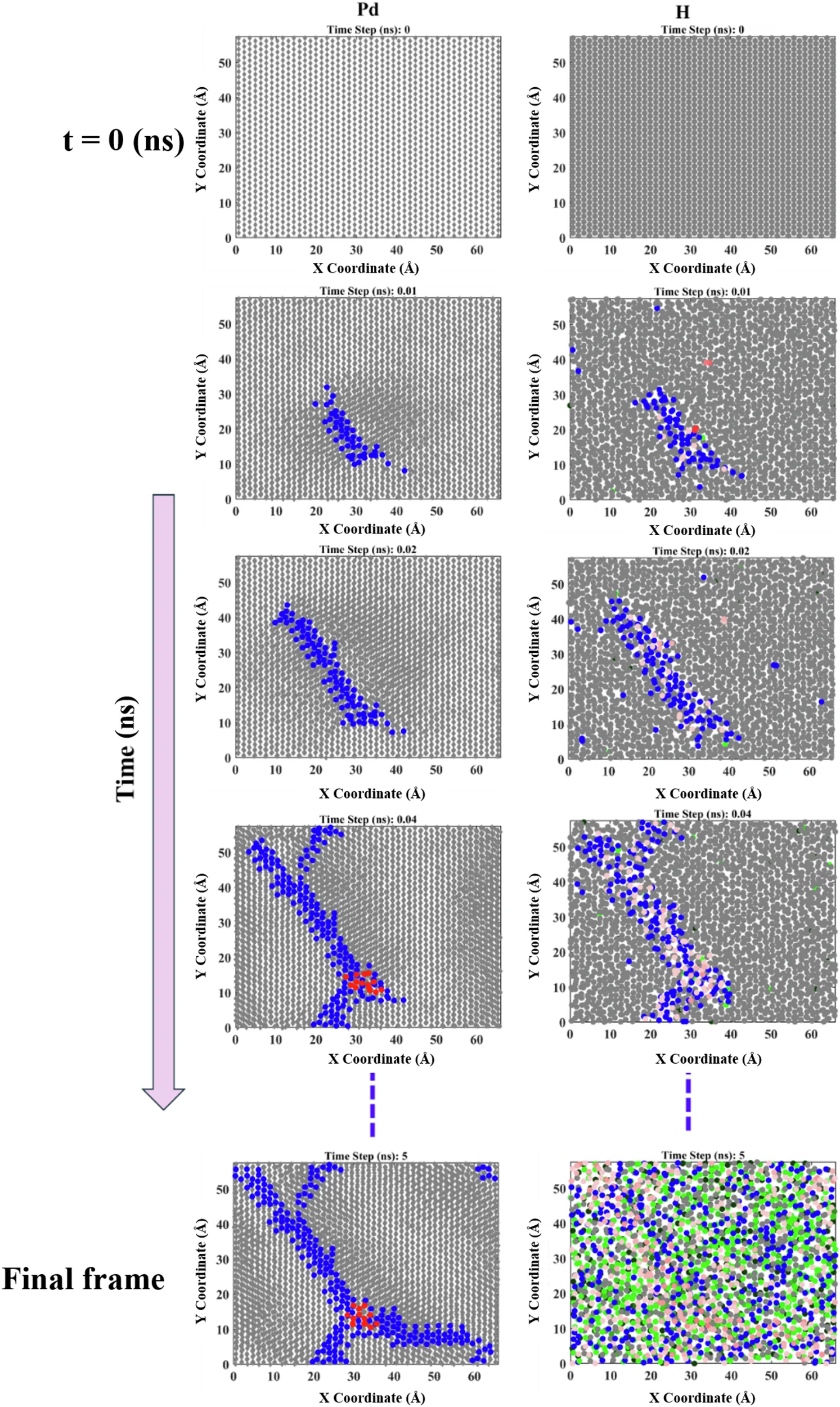
Pd migration begins from three subsurface hydride layers. Representative frames from the (left panel) Pd and (right panel) H tracking analysis of PdH_3sub_/Pd(111), shown from top views. Gray, blue, red and green indicate atoms that remain in their original layer (gray), move one layer upward (blue), move two layers upward (red) and move one layer downward (green), respectively. Larger upward and downward migrations for H atoms are represented by progressively lighter shades of red and progressively darker shades of green, respectively.

**Fig. 4:**
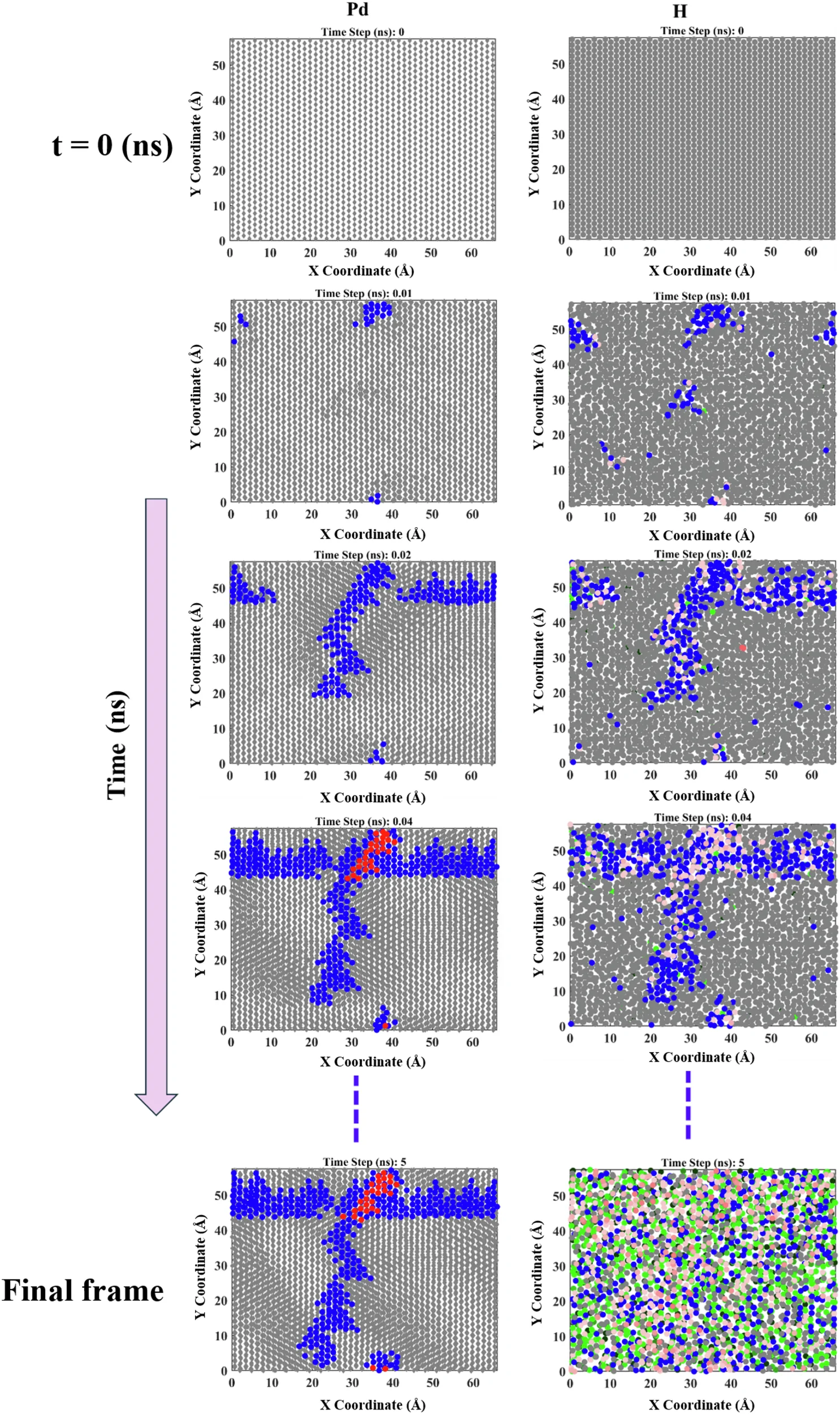
Pd migration increases at intermediate subsurface hydride loading. Representative frames from the (left panel) Pd and (right panel) H tracking analysis of PdH_4sub_/Pd(111), shown from top views. Gray, blue, red and green indicate atoms that remain in their original layer (gray), move one layer upward (blue), move two layers upward (red) and move one layer downward (green), respectively. Larger upward and downward migrations for H atoms are represented by progressively lighter shades of red and progressively darker shades of green, respectively.

**Fig. 5:**
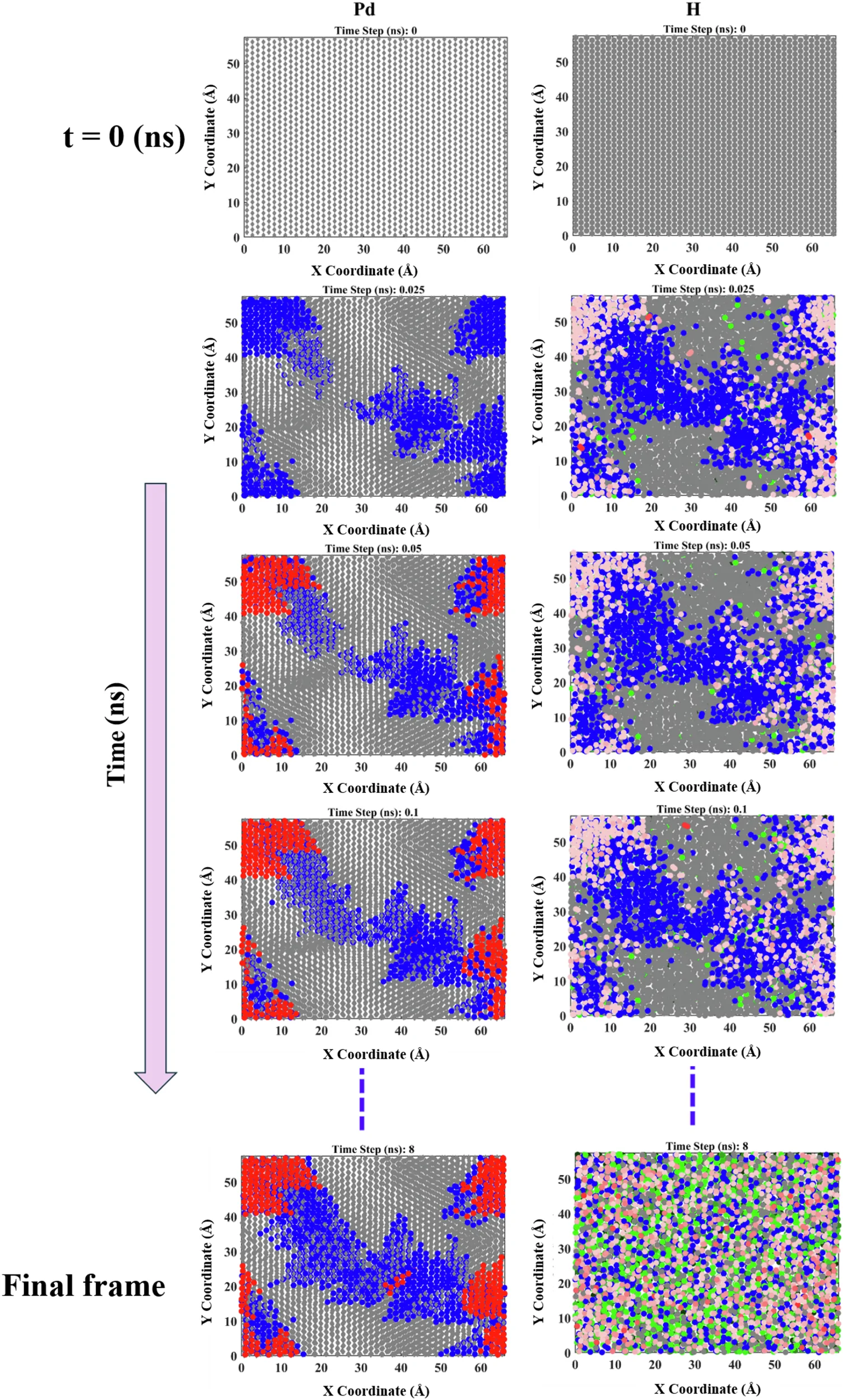
Extensive Pd migration occurs at high subsurface hydride loading. Representative frames obtained from (left panel) Pd and (right panel) H tracking analysis for PdH_9sub_/Pd(111) from top views. Gray, blue, red, and green indicate atoms that remain in their original layer (gray), move one layer upward (blue), move two layers upward (red), and move one layer downward (green), respectively. Larger upward and downward migrations for H atoms are represented by progressively lighter shades of red and progressively darker shades of green, respectively.

MD simulations were performed at 300 K for the PdH_x_/Pd(111) slab models with 0 (PdH_0subH_ + 1.00 ML H*, or PdH_0sub_), 1 (PdH_1subH_ + 1.00 ML H*, or PdH_1sub_), 2 (PdH_2subH_ + 1.00 ML H*, or PdH_2sub_), 3 (PdH_3subH_ + 1.00 ML H*, or PdH_3sub_), 4 (PdH_4subH_ + 1.00 ML H*, or PdH_4sub_) H sublayers for 5 ns, and with 9 (PdH_9subH_ + 1.00 ML H*, or PdH_9sub_) initial H sublayers for 8 ns to obtain the equilibrated structures^22^. The reoptimized surface structures after 5 ns MD equilibration at 300 K are displayed in Fig. S2. The figure shows that surface roughening remains negligible in systems containing up to two hydrogen sublayers. However, in systems containing three or more hydrogen sublayers, surface roughening and restructuring become apparent, consistent with the migration of some subsurface Pd atoms to the surface. As our previous study provided in-depth insights into reconstructed surfaces and the emergence of defects due to strain relaxation in PdH_x_/Pd(111), our focus here is specifically on atom mobility. To obtain a qualitative picture of the Pd mobility, we tracked Pd atomic movements during the simulations by color-coding each sublayer and monitoring their trajectories over time (Figs. S3–S5). Our analysis indicates that Pd atoms migrate through both one-step and two-step jumps. To examine this migration behavior in detail, we performed a tracking analysis in which we initially labeled the sublayers uniformly in gray. During the simulation, the atoms were tracked, and their color codes were updated according to their movement: blue for Pd atoms moving upwards by one adjacent layer, red for those moving upwards by two layers, and green for those moving downwards (see Figs. 3–5 and S6–S9). For atoms moving upwards by more than two layers, progressively lighter shades of red were used, whereas progressively darker shades of green indicate greater downward movement. Atoms that remained within their original sublayer retained their gray color. Representative frames from the tracking analysis of Pd atoms are presented in Figs. 3–5. Within the simulated systems, appreciable Pd migration, predominantly in the upward direction, is observed only when at least three hydride layers are present. In systems with three and four hydride sublayers, surface Pd atoms migrated from up to two layers in the z-direction, forming islands and adatoms, while Pd atoms near the PdH_x_/Pd predominantly undergo single-layer jumps (Figs. 3 and 4). In contrast, Pd atoms in the bulk region (with no hydrogen atoms) remained stationary, suggesting that hydrogen incorporation plays a crucial role in atomic mobility. At higher hydride loadings, exemplified by the system containing nine subsurface hydride layers, Pd atoms in layers near the surface undergo jumps of at least two layers, accompanied by more extensive restructuring (Fig. 5).

To further validate our hypothesis on the critical role of hydrogen incorporation for Pd mobility, we conducted a tracking analysis for all H atoms. The obtained frames are presented in the Supporting Information. The initial and final frames for systems with various H sublayers are shown in Figs. S10–S15, while Figs. 3–5 present Pd and H tracking frames together for systems with 3, 4, and 9 H sublayers. In systems with 0, 1, and 2 H sublayers, we observe that several H atoms on the surface or in sublayers close to the surface remain in place without significant upward or downward movement (Figs. S10–S12). The only movement observed in these systems involves the downward jump of some H atoms, which can be ascribed to Pd’s ability to absorb H atoms even at 298.15 K. In addition, no Pd jumps were detected in these systems, meaning no significant surface reconstruction or Pd island formation occurred at low H loading.

However, in systems with 3, 4, and 9 H sublayers, both upwards and downwards jumps of H atoms are observed (see Figs. 3–5), suggesting that a sufficient concentration of H atoms is required for upward movement to occur. Based on our observations, at least three H sublayers are required to facilitate Pd movements. Furthermore, the tracking analysis of Pd atoms reveals movement and reconstruction, particularly at the locations where H atoms exhibit upward jumps. This correlation indicates that the upward movement of H atoms drives the Pd atoms to migrate in the same direction and thus causes surface reconstruction. Additionally, we observed that, at specific time frames across all systems, H atoms exhibit greater mobility than Pd atoms. For instance, at 0.01 ns, while Pd atoms undergo only their first upward jump, some H atoms have already completed their second or even more jumps, either upward or downward (see Figs. 3–5). Overall, we can observe that the evolution of Pd atoms is majorly in the direction of H movement, until an equilibrium is reached, above which the H atoms jiggle to fit themselves in the reconstructed Pd lattice.

**Fig. 6:**
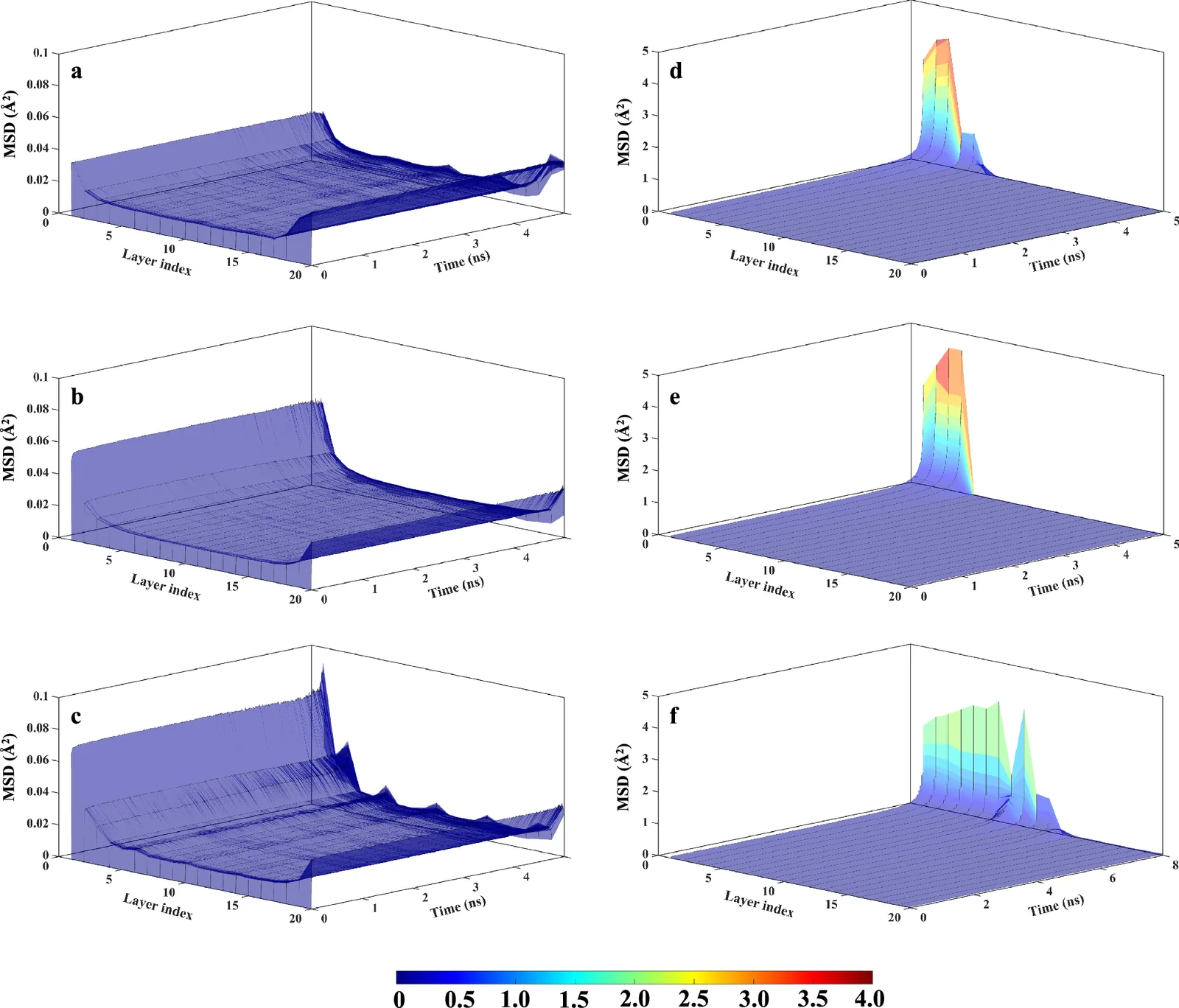
Subsurface hydride loading enhances out-of-plane Pd mobility. Mean square displacement along the z direction for each layer of Pd for **a** PdH_0sub_/Pd(111), **b** PdH_1sub_/Pd(111), **c** PdH_2sub_/Pd(111), **d** PdH_3sub_/Pd(111), **e** PdH_4sub_/Pd(111), and **f** PdH_9sub_/Pd(111). Source data are provided as a Source Data file.

**Fig. 7:**
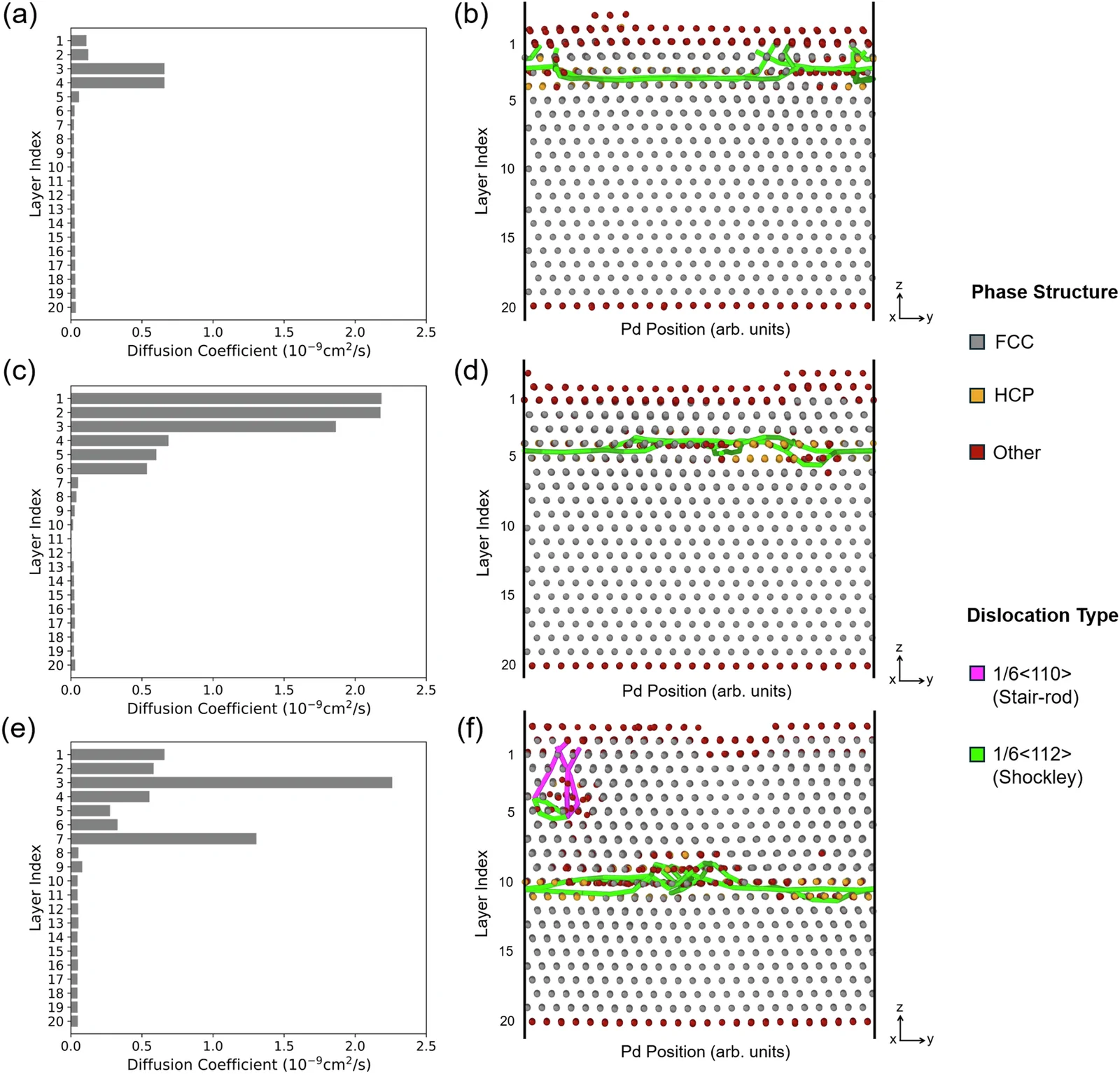
Hydride loading enhances out-of-plane Pd diffusion and structural disorder. Diffusion coefficient (D_zz_) along the z direction for Pd atoms in each layer and visualization of the local structures and dislocation after MD equilibration at 300 K for **a**, **b** PdH_3sub_/Pd(111), **c**, **d** PdH_4sub_/Pd(111), and **e**, **f** PdH_9sub_/Pd(111). Source data are provided as a Source Data file.

The mean square displacement (MSD) analysis in the z direction further supports this trend, where in systems with 0–2 hydride sublayers, Pd atoms remained largely undisturbed over time, whereas in systems with 3–4 hydride sublayers, noticeable surface changes were observed as the structure evolved (see Fig. 6). In the case of the 9-sublayer system, distortions were observed up to the PdH_x_/Pd interface, while the bulk region remained structurally stable. These results show that increasing hydride concentration induces greater structural changes. Additionally, in the case of the PdH_9sub_ system, we observed that MSD_zz_ of the 9^th^ Pd layer is higher than that of the 8^th^ and 10^th^ layers, which could be attributed to the dislocation region caused in the PdH_x_/Pd interface (see Fig. 7f). Dislocations typically occur around the β-hydride/α-hydride interface, providing extra room for the Pd atoms to move. We performed a mean square displacement (MSD) analysis in the z direction for the hydrogen layers in the PdH_3sub_, PdH_4sub_, and PdH_9sub_ systems (see Fig. S16). A comparison between Figs. S16 and 6 shows that the MSD curves for the hydrogen layers increase at a sharper rate than those for the palladium layers. This observation, together with the tracking analysis results, suggests that the upward movement of hydrogen atoms is faster and may drive the displacement of palladium atoms.

Finally, to quantify the effect of H loading on the Pd migration, the diffusion coefficients of Pd were calculated for each system in the z direction (Fig. 7). Notably, Pd diffusion coefficients increase with more hydride layers, until saturation is reached (like in PdH_9sub_/Pd(111)), highlighting the accelerating effect of hydriding on surface reconstructions. It is important to note that the pre-hydrided slab models employed here represent an idealized, hydrogen-rich regime corresponding to post α→β phase transition conditions rather than the dilute α-PdH_x_ phase. These models are not intended to reproduce the full electrochemical hydrogen uptake pathway, including adsorption, diffusion into the bulk, and dynamic coupling to the applied potential. Instead, they are designed to isolate the structural and mechanical response of Pd surfaces once substantial subsurface hydrogen accumulation has been established. Consistent with physical expectations, our MD simulations show that hydrogen redistributes rapidly within the lattice prior to significant Pd atom migration, implying that Pd mobility is enabled only after sufficient local hydride formation. Thus, the reported diffusivities and reconstruction behavior should be interpreted as characteristic of strongly hydrided, cathodic conditions where hydrogen-rich PdH_x_ phases are thermodynamically stable, rather than as descriptors of early-stage dilute hydrogen incorporation.

Overall, this study provides key insights into the role of hydride formation in initiating Pd atom mobility within Pd cathodes, where PdH_x_/Pd(111) interfaces develop under reducing conditions. By combining density functional theory and large-scale molecular dynamics simulations, we show that the extent of hydriding modulates Pd migration kinetics and the associated strain-driven surface reconstruction. Our DFT calculations suggest that Pd migration barriers decrease progressively with increasing subsurface hydrogen concentration, consistent with hydride-induced lattice expansion and softening. Complementary MD simulations indicate that sufficiently high hydride loading is required to trigger significant Pd mobility, with Pd atoms preferentially migrating in the direction of hydrogen redistribution until structural equilibration is achieved. Together, these results suggest a link between subsurface hydrogen accumulation, strain relaxation, and atomic-scale restructuring at Pd surfaces. The findings underscore the importance of considering dynamic, hydride-induced structural transformations under cathodic polarization, particularly when controlling hydrogen concentrations during electrochemical reactions such as the hydrogen evolution reaction. More broadly, this work highlights how multiscale computational approaches can help elucidate condition-dependent surface transformations that may influence catalyst stability and performance.

## Methods

### Density functional theory

Spin-polarized DFT calculations were performed using the Vienna Ab initio Simulation Package (VASP 5.4.4)^62^. The exchange-correlation function was described using the generalized gradient approximation (GGA) as parameterized by Perdew, Burke, and Ernzerhof (PBE)^63,64^. The projector-augmented wave (PAW) method was employed to describe the core-valence electron interactions, considering the 4d^10^ and 1s^1^ valence electrons for Pd and H atoms, respectively^65^. The plane-wave energy cutoff was set to 500 eV to ensure convergence. For geometry optimization, the convergence criteria were set with an electronic energy threshold of 1.0 × 10^−5^ eV and a force threshold of 0.01 eV/Å. Brillouin zone integration was performed using the Gaussian smearing method with a smearing width of 0.05 eV^66^. Van der Waals interactions were accounted for using Grimme’s D3 dispersion correction with Becke–Johnson damping, as implemented in VASP^67,68^. Geometry optimization and nudged elastic band (NEB) calculations employed the same parameters to maintain consistency. The NEB method was applied to determine the minimum energy pathways for Pd migration^69^. A climbing-image NEB approach was employed with seven intermediate images constructed between the initial and final states^70^. The spring constant was set to –5.0 eV/Å^**2**^ to ensure stable convergence of the NEB pathway, and the atomic forces were converged below 0.06 eV **Å**^−1^. To assess the influence of the dispersion correction on the calculated migration barriers, additional NEB calculations were also performed without the D3-BJ correction; the corresponding results are provided in Figs. S20 and S21.

### DFT models

For the DFT calculations, PdH_x_/Pd(111) models were constructed by cleaving the optimized Pd bulk with a lattice constant of 3.891 Å (Fig. S17). The 111 facet was chosen based on previous experimental and computational studies, where it was shown to be highly stable and commonly exposed over the synthesized nanoparticles^10,71^. The models consisted of 4 × 4 × 1 supercells with 6 layers of Pd, and included a vacuum layer of ∼20 Å to avoid interactions between periodic images. A Γ-centered k-point mesh of 3 × 3 × 1 was used during the structural optimization of the Pd systems and their adsorbate-surface slabs.

To study the hydriding effect, a monolayer of H atoms was added at the octahedral voids in each sublayer, up to three sublayers. The models for studying Pd migration from the first subsurface to the surface were obtained by removing a Pd atom from the surface, thereby creating a surface vacancy (void). Based on our hypothesis of the ironing or surface-healing process, the DFT study was restricted to investigating migration from only the first sublayer.

### Surface Pourbaix diagrams

Surface Pourbaix Diagrams (SPDs) are constructed by using the potential (${U}_{{SHE}}$) and pH-dependent change in Gibbs free energy ($\Delta {G}_{{H}^{*}}$) equation for hydrogen adsorption:

1 $$\Delta {G}_{{H}^{*}}\left({pH},{U}_{{SHE}}\right)={G}_{{{nH}}^{*}}-{G}_{*}-{\rm{n}}\left(\frac{1}{2}{G}_{{H}_{2}}^{0}-{{\rm{k}}}_{{\rm{B}}}T{\mathrm{ln}}\left(10\right).{pH}-{\rm{e}}.{U}_{{SHE}}\right)$$

where ${{\rm{G}}}_{{{\rm{nH}}}^{*}}$, and ${{\rm{G}}}_{*}$ are the Gibbs free energies of the system with “n” hydrogen atoms adsorbed, and the clean surface without any H atoms, respectively. ${G}_{{H}_{2}}^{0}$ is the Gibbs free energy of an H_2_ gas molecule placed in a supercell with dimensions of $20\times 20\times 20$ Å^3^. T is the temperature (herein, *T* = 298.15 K) and ${{\rm{k}}}_{{\rm{B}}}$ is the Boltzmann constant. A detailed explanation of the SPD construction can be found in our previous works^71–73^.

${{\rm{G}}}_{n{{\rm{H}}}^{*}}$ can be further described by incorporating vibrational contributions:

2 $${G}_{{{nH}}^{*}}={E}_{{DFT}}+{E}_{{ZPVE}}+{\Delta E}_{{vib}}\left({\rm{T}}\right)-T{S}_{{vib}}\approx {E}_{{DFT}}+{F}_{{vib}}\left({\rm{T}}\right)$$

where ${{\rm{E}}}_{{\rm{DFT}}}$ represents the total energy, and ${{\rm{F}}}_{{\rm{vib}}}$ is the vibrational energy contributions calculated using:

3 $${F}_{{vib}}(T)={E}_{{ZPVE}}+{\Delta E}_{{vib}}\left(T\right)-T.{S}_{{vib}}=\frac{1}{2}{\sum }_{i}\left\{{\rm{h}}{{\rm{\nu }}}_{{\rm{i}}}+2{{\rm{k}}}_{{\rm{B}}}T{\mathrm{ln}}\left[1-\exp \left(-\frac{{\rm{h}}{{\rm{\nu }}}_{{\rm{i}}}}{{{\rm{k}}}_{{\rm{B}}}T}\right)\right]\right\}$$

where ${v}_{i}$ represents the vibrational frequencies of hydrogen atoms on the adsorbed surfaces. ${{\rm{G}}}_{{{\rm{H}}}_{2}}^{0}$ is the Gibbs free energy for an H_2_ molecule in the gas-phase at standard conditions (*P* = 1 bar, U = 0 V and pH = 0). The ${{\rm{G}}}_{{{\rm{H}}}_{2}}^{0}$ is calculated as:

4 $${G}_{{H}_{2}}^{0}={E}_{{DFT}}+{E}_{{ZPVE}}+{\Delta E}_{{vib}}\left(T\right)-T\left({S}_{{vib}}+{S}_{{trans}}+{S}_{{rot}}\right)+\int _{0}^{298.15}{C}_{p}{dT}+{PV}$$

which simplifies to:

5 $${G}_{{H}_{2}}^{0}={E}_{{DFT}}+{F}_{{vib}}\left(T\right)-T\left({S}_{{trans}}+{S}_{{rot}}\right)+\int _{0}^{298.15}{C}_{p}{dT}+{PV}$$

Here ${\int }_{0}^{298.15}{{\rm{C}}}_{{\rm{p}}}{\rm{d}}T$ represents the heat capacity contribution, and ${{\rm{S}}}_{{\rm{trans}}}$ and ${{\rm{S}}}_{{\rm{rot}}}$ are the translational and rotational entropies, respectively. These values are calculated using the Sackur-Tetrode equations, as detailed in our previous work on pristine and defective Pd surfaces^61,71^.

### Molecular dynamics simulations

Molecular dynamics (MD) simulations were performed using the Large-scale Atomic/Molecular Massively Parallel Simulator (LAMMPS) package^74^, with the ML_PACE package^75^. Interatomic interactions were described using atomic cluster expansion (ACE) potentials^76^, parametrized and iteratively trained for the Pd–H system. The training dataset comprised a diverse set of PBE-D3 calculations, including elemental Pd and H phases as well as various Pd–H binary structures, generated using the Pacemaker package^77^. The reliability of the ACE-D3 potential for describing both bulk and surface properties of Pd and PdH crystals has been established in earlier work^22,60,78^.

Prior to the MD simulations, energy minimization of all structures was employed with LAMMPS using the conjugate gradient method, with a total energy tolerance of 10^−10^ eV and atomic force convergence of 10^−10^ eV/Å, while maintaining a constant cell shape and volume. The energy-minimized structures were then subjected to canonical ensemble (NVT) MD simulations at 300 K, using the Nosé-Hoover thermostat with a temperature damping parameter of 0.05 ps. A 1 fs time step was used for all simulations. After the production runs, the final structures were minimized again using the conjugate gradient method, maintaining constant cell shape and volume for post-analysis. Visualization of surfaces and dislocation structures was performed using the Open Visualization Tool (OVITO) software^79^. Additionally, Visual Molecular Dynamics (VMD) software was utilized to monitor the movement of Pd sublayers during the MD simulation^80^.

### Diffusion coefficients

To comprehensively understand the dynamics of the Pd-H system, we calculated MSD in the zz spatial dimensions. This analysis provides insights into the directional diffusion behavior of Pd atoms moving within the lattice. ${{MSD}}_{{zz}}$ is obtained using the following equation.

6 $${{MSD}}_{{zz}}=\left\langle {\left[z\left(t+\Delta t\right)-z\left(t\right)\right]}^{2}\right\rangle$$

To extract the diffusivity coefficient from MSD data, we applied the Einstein-Smoluchowski relation^81,82^, which relates the diffusivity D to the MSD. The equation can be generalized to relate the mean square displacement tensor, and the diffusion tensor as follows:

7 $${D}_{{zz}}=\frac{1}{2d}\frac{{{MSD}}_{{zz}}\left(\Delta t\right)}{\Delta t}\,$$

8 $${\rm{Or}}{D}_{{zz}}=\mathop{{lim}}\limits_{t\to+\infty }\frac{1}{2d}\frac{\,{\rm{MSD}}\,{{zz}}{(t)}}{t}\,$$

*d* is the dimensionality of the system and Δt the time interval. By examining the MSD plots, we computed the diffusion coefficients along different directions within a three-dimensional space (d = 3). This involved fitting MSD data with respect to the lag-time to a linear model.

### MD models

The MD simulations were performed on a Pd(111) slab derived from the DFT-optimized bulk structure (a = 3.891 Å), consisting of 20 atomic layers with 576 Pd atoms per layer. For the initial PdH_x_/Pd(111) configurations, the surface was fully covered with one monolayer of hydrogen atoms at three-fold fcc hollow sites, while subsurface hydrogen atoms were placed in octahedral interstitial sites. Under high subsurface hydrogen concentration (β-PdH_x_), it is shown that surface reconstruction and roughening occur due to strain relaxation, with H predominantly occupying octahedral sites in the upper half of the Pd slab models^22^. To investigate the role of the degree of hydriding on Pd mobility, we used the Pd slab models with pre-absorbed subsurface hydrogen, specifically with 0, 1, 2, 3, 4, and 9 H sublayers. These models enable a direct assessment of how H penetration depth influences Pd migration rates and consequently the onset of surface reconstruction (Fig. S18).

## Supplementary information

Supplementary Information

Description of Additional Supplementary Files

Supplementary Data 1

Transparent Peer Review file

## Source data

Source Data

## Data Availability

The source data underlying all graphs in the main and Supplementary Figures are provided with this paper as a Source Data file. The optimized atomic coordinates of the computational models and the initial and final configurations of the molecular dynamics simulations are provided in this paper as Supplementary Data 1. Source data are provided with this paper.

## Code availability

The Pd–H-specific ACE–PBE-D3 machine-learning interatomic potential and its training dataset are available through Zenodo at https://doi.org/10.5281/zenodo.20591787^83^. The custom scripts developed for post-processing and analysis of the molecular dynamics trajectories are available from the corresponding authors upon request.

## References

[1] Li, H. X. & Li, G. Novel palladium-based nanomaterials for multifunctional ORR/OER/HER electrocatalysis. *J. Mater. Chem. A* **11**, 9383–9400 (2023).

[2] Boasiako, C. A., Zhou, Z., Huo, X. C. & Ye, T. Development of Pd-based catalysts for hydrogenation of nitrite and nitrate in water: a review. *J. Hazard Mater.* **446**, 130661 (2023). 10.1016/j.jhazmat.2022.130661 36587602

[3] Joudeh, N., Saragliadis, A., Koster, G., Mikheenko, P. & Linke, D. Synthesis methods and applications of palladium nanoparticles: a review. *Front. Nanotechnol.* 10.3389/fnano.2022.1062608 (2022).

[4] Voskresenskaya, E. N., Kirilets, V. M., Taran, O. P. & Kuznetsov, B. N. Hydrogen production by the heterogeneous catalytic dehydrogenation of formic acid: a review. *Catal. Ind.* **16**, 339–349 (2024).

[5] Darmadi, I., Nugroho, F. A. A. & Langhammer, C. High-performance nanostructured palladium-based hydrogen sensors-current limitations and strategies for their mitigation. *Acs Sens.* **5**, 3306–3327 (2020). 10.1021/acssensors.0c02019 PMC7735785 33181012

[6] Goods, S. H. & Guthrie, S. E. Mechanical-properties of palladium and palladium hydride. *Scr. Met. Mater.* **26**, 561–565 (1992).

[7] DeNinno, A., LaBarbera, A. & Violante, V. Deformations induced by high loading ratios in palladium-deuterium compounds. *J. Alloy Compd.* **253**, 181–184 (1997).

[8] Wang, Q. R., Zhang, S. Y., Guo, J. P. & Chen, P. Advances in highly hydrided palladium. *Front. Mater.* **11** 10.3389/fmats.2024.1365526 (2024).

[9] Fukai, Y. & Okuma, N. Formation of superabundant vacancies in Pd hydride under high hydrogen pressures. *Phys. Rev. Lett.* **73**, 1640–1643 (1994). 10.1103/PhysRevLett.73.1640 10056846

[10] Schmidt, T. O. et al. Elucidation of structure-activity relations in proton electroreduction at Pd surfaces: theoretical and experimental study. *Small* **18**, e2202410 (2022). 10.1002/smll.202202410 35726004

[11] Suzana, A. F. et al. Structure of a seeded palladium nanoparticle and its dynamics during the hydride phase transformation. *Commun. Chem.* **4**, 64 (2021). 10.1038/s42004-021-00500-7 PMC9814609 36697569

[12] Setayandeh, S. S., Gould, T., Vaez, A. & Gray, E. Effect of pseudopotential choice on the calculated electron and phonon band structures of palladium hydride and its vacancy defect phases. *Int J. Hydrog. Energ.* **46**, 943–954 (2021).

[13] Wagner, S. & Pundt, A. Hydrogen as a probe for defects in materials: Isotherms and related microstructures of palladium-hydrogen thin films. *Aims Mater. Sci.* **7**, 399–419 (2020).

[14] Burlaka, V., Roddatis, V., Bongers, M. D. & Pundt, A. Defect generation in Pd layers by ‘smart’ films with high H-affinity. *Sci. Rep.-Uk* **7**, 9564 (2017). 10.1038/s41598-017-09900-z PMC5573398 28842694

[15] Melikhova, O., Cízek, J. & Procházka, I. Hydrogen-induced defects in palladium. *Acta Phys. Pol. A* **125**, 752–755 (2014).

[16] Trinkle, D. R., Ju, H.S., Heuser, B. J. & Udovic, T. J. Nanoscale hydride formation at dislocations in palladium: theory and inelastic neutron scattering measurements. *Phys. Rev. B* **83**, 174116 (2011).

[17] Zhirov, G. I., Gol’tsov, V. A. & Glyakov, D. A. Mechanical properties of palladium hydride after annealing and hydrogen-induced transformation hardening. *Phys. Met Metallogr.* **97**, 104–110 (2004).

[18] Abe, H. et al. Improvement of the hydrogen absorption rate of Pd by ion irradiation. *Nucl. Instrum. Meth B* **206**, 224–227 (2003).

[19] Storms, E. & Talcottstorms, C. The effect of hydriding on the physical structure of palladium and on the release of contained tritium. *Fusion Technol.* **20**, 246–257 (1991).

[20] Eastman, J. A., Fitzsimmons, M. R. & Thompson, L. J. Anomalous defect behavior resulting from the hydriding of nanocrystalline palladium. *Philos. Mag. Lett.* **68**, 335–343 (1993).

[21] Ulvestad, A. et al. Avalanching strain dynamics during the hydriding phase transformation in individual palladium nanoparticles. *Nat. Commun.* **6**, 10092 (2015). 10.1038/ncomms10092 PMC4682038 26655832

[22] Ngoipala, A. et al. Hydride-induced reconstruction of Pd electrode surfaces: a combined computational and experimental study. *Adv. Mater.* **37**, e2410951 (2024). 10.1002/adma.202410951 PMC11775870 39632662

[23] Camuti, L. et al. Phase-stable palladium hydride derived from PdCoO_2_ for sustainable hydrogen evolution reaction. *Adv. Funct. Mater.* **36**, e14366 10.1002/adfm.202514366 (2025).

[24] Hou, S. T. et al. Design of a stable PdH_0.706_ catalyst supported on a hydrophobic aromatic siloxane framework for hydrogenation reactions. *ACS Sustain Chem. Eng.* **14**, 8095–8110 (2026).

[25] Padavala, S. K. M. & Stoerzinger, K. A. Role of hydride formation in electrocatalysis for sustainable chemical transformations. *Acs Catal.* **13**, 4544–4551 (2023).

[26] Xia, G. J. & Wang, Y. G. Solvent promotion on the metal-support interaction and activity of Pd@ZrO_2_ Catalyst: formation of metal hydrides as the new catalytic active phase at the Solid-Liquid interface. *J. Catal.* **404**, 537–550 (2021).

[27] Zhang, Z. S., Wei, Z. Y., Sautet, P. & Alexandrova, A. N. Hydrogen-induced restructuring of a Cu(100) electrode in electroreduction conditions. *J. Am. Chem. Soc.* **144**, 19284–19293 (2022). 10.1021/jacs.2c06188 36227161

[28] Wei, J. et al. Role of surface hydroxyls in atomic-scale copper restructuring during CO electroreduction. *J. Am. Chem. Soc.* **147**, 45178–45188 (2025). 10.1021/jacs.5c14516 PMC12703730 41283916

[29] Zhang, Z. S., Gee, W., Sautet, P. & Alexandrova, A. N. H and CO Co-induced roughening of Cu surface in CO_2_ electroreduction conditions. *J. Am. Chem. Soc.* **146**, 16119–16127 (2024). 10.1021/jacs.4c03515 38815275

[30] Wei, Y., Sautet, P. & Alexandrova, A. N. Temperature-dependent adsorbate-induced surface roughening onset in electrochemical CO_2_ reduction on copper. *J. Phys. Chem. Lett.* **16**, 12193–12201 (2025). 10.1021/acs.jpclett.5c03061 41246974

[31] Yau, A., Harder, R. J., Kanan, M. W. & Ulvestad, A. Imaging the hydrogen absorption dynamics of individual grains in polycrystalline palladium thin films in 3D. *Acs Nano* **11**, 10945–10954 (2017). 10.1021/acsnano.7b04735 29035558

[32] Lumbeeck, G. et al. Effect of hydriding-induced defects on the small-scale plasticity mechanisms in nanocrystalline palladium thin films. *J. Appl. Phys.* **124**, 225105 (2018).

[33] Okuyama, H., Siga, W., Takagi, N., Nishijima, M. & Aruga, T. Path and mechanism of hydrogen absorption at Pd(100). *Surf. Sci.* **401**, 344–354 (1998).

[34] Sun, X. S. Exploring solute-defect interactions in nanosized palladium hydrides across multiple time scales. *Comp. Mater. Sci.* **231**, 112582 10.1016/j.commatsci.2023.112582 (2024).

[35] Zhou, S. Y. et al. Fast and non-equilibrium uptake of hydrogen by Pd Icosahedral nanocrystals. *Angew. Chem. Int Ed.* **62**, e202306906 (2023). 10.1002/anie.202306906 37528509

[36] Moumaneix, L., Rautakorpi, A. & Kallio, T. Interactions between hydrogen and palladium nanoparticles: resolving adsorption and absorption contributions. *Chemelectrochem* **10,** e202201109 10.1002/celc.202201109 (2023).

[37] Tang, J. Y. et al. Hydrogen absorption and diffusion behaviors in cube-shaped palladium nanoparticles revealed by ambient-pressure X-ray photoelectron spectroscopy. *Appl. Surf. Sci.* **587,** 152797 10.1016/j.apsusc.2022.152797 (2022).

[38] Schwarzer, M. et al. Adsorption and absorption energies of hydrogen with palladium. *J. Phys. Chem. C.* **126**, 14500–14508 (2022). 10.1021/acs.jpcc.2c04567 PMC9442642 36081903

[39] Sakagami, H., Tachikawa, M. & Ishimoto, T. Hydrogen/deuterium adsorption and absorption properties on and in palladium using a combined plane wave and localized basis set method. *Int. J. Quantum Chem.* **120**, e26275 10.1002/qua.26275 (2020).

[40] Sun, X., Ariza, M. P., Ortiz, M. & Wang, K. G. Atomistic modeling and analysis of hydride phase transformation in palladium nanoparticles. *J. Mech. Phys. Solids* **125**, 360–383 (2019).

[41] Zhang, L. N., Qiao, L. J., Bligaard, T. & Su, Y. J. A first-principles study of H adsorption and absorption under the influence of coverage. *Appl. Surf. Sci.* **457**, 280–286 (2018).

[42] Senftle, T. P., Janik, M. J. & van Duin, A. C. T. A ReaxFF investigation of hydride formation in palladium nanoclusters via Monte Carlo and molecular dynamics simulations. *J. Phys. Chem. C.* **118**, 4967–4981 (2014).

[43] Ohno, S., Wilde, M. & Fukutani, K. Novel insight into the hydrogen absorption mechanism at the Pd(110) surface. *J. Chem. Phys.* **140**, 134705 (2014). 10.1063/1.4869544 24712806

[44] Padama, A. A. B., Kasai, H., Budhi, Y. W. & Arboleda, N. B. Investigation of hydrogen atom adsorption and absorption on Pd(110) surface. *J. Phys. Soc. Jpn.* **81**, 114705 (2012).

[45] Hong, S. & Rahman, T. S. Adsorption and diffusion of hydrogen on Pd(211) and Pd(111): results from first-principles electronic structure calculations. *Phys. Rev. B* **75**, 155405 (2007).

[46] Jewell, L. L. & Davis, B. H. Review of absorption and adsorption in the hydrogen-palladium system. *Appl. Catal. a-Gen.* **310**, 1–15 (2006).

[47] Zhang, W. S., Zhang, Z. L. & Zhang, X. W. Effects of temperature on hydrogen absorption into palladium hydride electrodes in the hydrogen evolution reaction. *J. Electroanal. Chem.* **481**, 13–23 (2000).

[48] Polfus, J. M., Peters, T., Bredesen, R. & Lovvik, O. M. Vacancy diffusion in palladium hydrides. *Phys. Chem. Chem. Phys.* **23**, 13680–13686 (2021). 10.1039/d1cp01960k 34124732

[49] Beszeda, I., Gontier-Moya, E. G. & Beke, D. L. Investigation of mass transfer surface self-diffusion on palladium. *Surf. Sci.* **547**, 229–238 (2003).

[50] Chen, G. X., Zhang, J. M., Xu, K. W. & Ji, V. Computer simulation study of self-diffusion in Pd(001) surface. *J. Phys. Chem. Solids* **68**, 389–393 (2007).

[51] Chen, G. X., Zhang, J. M., Xu, K. W. & Ji, V. Computer simulation study of self-diffusion in Pd(111) surface. *J. Mol. Catal. a-Chem.* **258**, 341–345 (2006).

[52] Liu, F. S. et al. Self-diffusion behaviors of Pd adatom and dimer on Pd(001) surface. *Comp. Mater. Sci.* **47**, 501–505 (2009).

[53] Liu, F. S. et al. Dynamics diffusion behaviors of Pd small clusters on a Pd(111) surface. *Model. Simul. Mater. Sci. Eng.* **18**, 045010 10.1088/0965-0393/18/4/045010 (2010).

[54] Baník, I., Lukovičová, J. & Pavlendová, G. in *Defect and Diffusion Forum* **353**, 292–297 (Trans Tech Publ, 2014).

[55] Kim, S. Y., Lee, I. H. & Jun, S. Transition-pathway models of atomic diffusion on fcc metal surfaces. I. Flat surfaces. *Phys. Rev. B* **76**, 245407 (2007).

[56] Kim, S. Y., Lee, I. H. & Jun, S. Transition-pathway models of atomic diffusion on fcc metal surfaces. II. Stepped surfaces. *Phys. Rev B* **76**, 245407 (2007).

[57] Liu, F. S. et al. Anisotropy diffusion dynamics behaviors on Pd(110) surfaces: a molecular dynamics study. *Surf. Rev. Lett.* **22**, 1550013 (2015).

[58] Ding, Y. et al. Hydrogen-enhanced grain boundary vacancy stockpiling causes a transgranular to intergranular fracture transition. *Acta Mater.* **239**, 118279 (2022).

[59] Luo, H. Y. et al. Point-defect modulation of hydrogen migration in Pd-based membranes. *J. Membr. Sci.* **713**, 123285 (2025).

[60] Atlan, C. et al. Probing Strain in Individual Palladium Nanocrystals during Electrochemically Induced Phase Transitions. *J. Am. Chem. Soc.* **147**, 25417–25428 (2025). 10.1021/jacs.5c05102 40637705

[61] Lipin, R., Ngoipala, A., Arevalo, R. L. & Vandichel, M. Computational surface Pourbaix diagrams to unravel cathodic hydride formation on defective palladium surfaces. *Int J. Hydrog. Energ.* **61**, 460–472 (2024).

[62] Kresse, G. & Furthmuller, J. Efficient iterative schemes for ab initio total-energy calculations using a plane-wave basis set. *Phys. Rev. B Condens Matter.* **54**, 11169–11186 (1996). 10.1103/physrevb.54.11169 9984901

[63] Perdew, J. P., Burke, K. & Ernzerhof, M. Generalized gradient approximation made simple. *Phys. Rev. Lett.* **77**, 3865 (1996). 10.1103/PhysRevLett.77.3865 10062328

[64] Perdew, J. P., Burke, K. & Wang, Y. Generalized gradient approximation for the exchange-correlation hole of a many-electron system. *Phys. Rev. B Condens Matter.* **54**, 16533–16539 (1996). 10.1103/physrevb.54.16533 9985776

[65] Blochl, P. E. Projector augmented-wave method. *Phys. Rev. B Condens Matter* **50**, 17953–17979 (1994). 10.1103/physrevb.50.17953 9976227

[66] Jorgensen, J. J. & Hart, G. L. Effectiveness of smearing and tetrahedron methods: best practices in DFT codes. *Model. Simul. Mater. Sci. Eng.* **29**, 065014 (2021).

[67] Grimme, S. Accurate description of van der Waals complexes by density functional theory including empirical corrections. *J. Comput Chem.* **25**, 1463–1473 (2004). 10.1002/jcc.20078 15224390

[68] Grimme, S., Ehrlich, S. & Goerigk, L. Effect of the damping function in dispersion corrected density functional theory. *J. Comput Chem.* **32**, 1456–1465 (2011). 10.1002/jcc.21759 21370243

[69] Henkelman, G. & Jónsson, H. Improved tangent estimate in the nudged elastic band method for finding minimum energy paths and saddle points. *J. Chem. Phys.* **113**, 9978–9985 (2000).

[70] Henkelman, G., Uberuaga, B. P. & Jónsson, H. A climbing image nudged elastic band method for finding saddle points and minimum energy paths. *J. Chem. Phys.* **113**, 9901–9904 (2000).

[71] Ngoipala, A., Lipin, R., Arevalo, R. L. & Vandichel, M. Computational unravelling of cathodic hydride formation on palladium surfaces. *Int J. Hydrog. Energ.* **53**, 829–839 (2024).

[72] Rodriguez-Olguin, M. A. et al. Temperature promotes selectivity during electrochemical CO_2_ reduction on NiO:SnO_2_ nanofibers. *J. Mater. Chem. A Mater.* **12**, 32821–32835 (2024). 10.1039/d4ta04116j PMC11363033 39219709

[73] Rodriguez-Olguin, M. A. et al. Chlorine in NiO promotes the electroreduction of CO2 to formate. *Appl. Mater. Today* **28**, 101528 (2022).

[74] Thompson, A. P. et al. LAMMPS-a flexible simulation tool for particle-based materials modeling at the atomic, meso, and continuum scales. *Comput. Phys. Commun.* **271**, 108171 (2022).

[75] Lysogorskiy, Y. et al. Performant implementation of the atomic cluster expansion (PACE) and application to copper and silicon. *Npj Comput. Mater.* 10.1038/s41524-021-00559-9 (2021).

[76] Xie, T. & Grossman, J. C. Crystal graph convolutional neural networks for an accurate and interpretable prediction of material properties. *Phys. Rev. Lett.* **120**, 145301 (2018). 10.1103/PhysRevLett.120.145301 29694125

[77] Bochkarev, A. et al. Efficient parametrization of the atomic cluster expansion. *Phys. Rev. Mater.* **6**, 013804 (2022).

[78] Viola, A. et al. Influence of the size and shape of palladium nanoparticles on their electrochemical hydrogen sorption capacity. *J. Am. Chem. Soc.* **148**, 9548–9562 (2026). 10.1021/jacs.5c19692 41758973

[79] Stukowski, A. Visualization and analysis of atomistic simulation data with OVITO-the Open Visualization Tool. *Model. Simul. Mater. Sci. Eng.* **18**, 015012 (2010).

[80] Humphrey, W., Dalke, A. & Schulten, K. V. M. D. Visual molecular dynamics. *J. Mol. Graph Model.* **14**, 33–38 (1996). 10.1016/0263-7855(96)00018-5 8744570

[81] Evangelista, L. R., Lenzi, E. K., Barbero, G. & Scarfone, A. M. On the Einstein-Smoluchowski relation in the framework of generalized statistical mechanics. *Phys. A: Stat. Mech. Appl.* **635**, 129491 (2024).

[82] Islam, M. A. Einstein-Smoluchowski diffusion equation: a discussion. *Phys. Scr.* **70**, 120–125 (2004).

[83] Mrovec, M., Vandichel, M., Ngoipala, A., Qamar, M. & Drautz, R. Hydride formation and phase separation in palladium nanoparticles from a transferable atomic cluster expansion potential. *Zenodo*10.5281/zenodo.20591787 (2026).

