# Supplementary material for: Hydride-induced palladium self-diffusion and surface restructuring on Pd(111)
Source: Nat Commun. 2026 Sep 15;17:10839. doi: 10.1038/s41467-026-77704-9 (PMC13672345; doi:10.1038/s41467-026-77704-9)
Supplement: Supplementary file 1 [file 41467_2026_77704_MOESM1_ESM.pdf]

Supplementary Information

Hydride-Induced Palladium Self-Diffusion and Surface Restructuring on Pd(111)

Raju Lipin[1], Muhammad Umer[1], Apinya Ngoipala[1], Sousa Javan Nikkhah[2,3,]* and Matthias Vandichel[1,]*

[1] Materials and Catalysis Modelling (MACATAMO) Group, Bernal Institute, University of Limerick, Limerick V94 T9PX, Ireland

[2] Simulation of Structures across Scales (SUSAS) Group, Department of Chemistry, Maynooth University, Maynooth, County Kildare W23 F2H6, Ireland

[3] Hamilton Institute; Kathleen Lonsdale Institute, Maynooth University, Maynooth, County Kildare W23 F2H6, Ireland

*Corresponding authors: Sousa.Javannikkhah@mu.ie, Matthias.Vandichel@ul.ie

Computational Methods

Vacancy Formation Energy

Expanding upon our previous findings, we introduced a single Pd vacancy on the surface and in the first subsurface of PdH_x/Pd(111) slab models with hydrogen contents ranging from 1 to 4 sublayers. Energy minimization was performed on all considered structures, and the Pd vacancy formation energy (ΔE_{vac}) was calculated for both surface and subsurface vacancies using the following equation:

$$\Delta E_{vac} = E_{\mathrm{slab+Pd-va}} + E_{\mathrm{Pd_1,bulk}} - E_{\mathrm{slab,perfect}} \quad (1)$$

where $E_{\mathrm{slab+Pd-vac}}$ and $E_{\mathrm{slab,perfect}}$ represent the total energies of the PdH_x/Pd(111) slab with a single Pd vacancy and the perfect PdH_x/Pd(111) slab, respectively, while $E_{\mathrm{Pd_1,bulk}}$ denotes the total energy of one Pd atom from bulk Pd with an fcc structure. Figure 1c shows the vacancy formation energies for the (4×4) unit cell model systems, while Figure S19 shows the same for large PdH_x/Pd(111) models prior to MD simulations.

As illustrated in Figure S19, the overall trend indicates that as the number of H layers increase, the vacancy formation energy decreases for both surface and subsurface vacancies for the MD models systems, consistent with the DFT results. This suggests that hydrogen absorption facilitates the formation of Pd vacancies. Initially, when there is only one H layer (only on the surface), the formation energy for a subsurface vacancy is significantly higher (1.235 eV) compared to a surface vacancy (0.810 eV). However, as more H layers are populated, the energy for both types of vacancies decreases. By the time the number of H layers reaches three, the formation energies of surface and subsurface vacancies converge to around 0.5 eV. Beyond this point, the values remain nearly identical, suggesting a saturation effect. This behavior can be explained by the fact that hydrogen absorption weakens the Pd-Pd bonds by increasing interatomic distances, thereby facilitating vacancy formation. These findings are consistent with our previous results, which showed that the formation of substantial hydride layers induces strain and surface reconstruction, causing Pd atoms to shift to accommodate the absorbed hydrogen.[1, 2]

Surface Energy

To access the relative stabilities of the Pd(111) surfaces, with varying H concentration, the surface energies were computed as:

$$\gamma_{Pd,clean} = \frac{1}{2A}\left(E_{Pd,slab} - N_{Pd}E_{Pd_1,bulk}\right) \qquad (2)$$

where $E_{Pd,slab}$ and $E_{Pd_1,bulk}$ are the total energies of the slab and one Pd atom, respectively. The $E_{Pd_1,bulk}$ energy was calculated from the total energy of a bulk Pd with an fcc structure. N_{Pd} and A are the number of Pd atoms per supercell and the surface area of the slab, respectively. Further, the effect of H concentration on the surface energies (γ) is calculated as:

$$\gamma = \gamma_{H^*/Pd} - \gamma_{Pd,clean} \qquad (3)$$

where $\gamma_{H^*/Pd}$ is the surface energy of a slab with hydrogen atoms on one side of the slab. This is computed as

$$\gamma_{H^*/Pd} = \frac{1}{A}\left(E_{Pd,slab+H^*} - \frac{N_H}{2}\mu_{H_2} - N_{Pd}E_{Pd_1,bulk}\right) \qquad (4)$$

where N_H and N_{Pd} are the number of H and Pd atoms in the supercell, respectively.

Areal Strain

The areal strain $\varepsilon_A(\%)$ of the Pd(111) models upon H loading (hydriding) is defined as

$$\varepsilon_A(\%) = \frac{A_{uc} - A_c}{A_c} \times 100 \qquad (5)$$

where A_{uc} and A_c are the areas of the unconstrained and constrained PdH_x/Pd(111) interface systems, respectively. Here, unconstrained refers to models relaxed using ISIF=4 in VASP, which allows in-plane lattice vectors to adjust freely in response to hydrogen-induced stress. In contrast, constrained models are fixed to the in-plane lattice constants of the clean Pd(111) surface (ISIF=2).

Work function

The work function (Φ) for the configurations along the diffusion pathway was calculated using the following equation:

$$\Phi = E_{vac} - E_F$$

where E_{vac} is the electrostatic potential energy in the vacuum level and E_F represent the Fermi energy. These calculations employed the same computational setting used for structural optimization, but with a denser Γ-centered 7×7×1 k-point mesh to ensure accurate determination of the electrostatic potential. The work function values were obtained from the planar-averaged electrostatic potential using VASPKIT.[3]

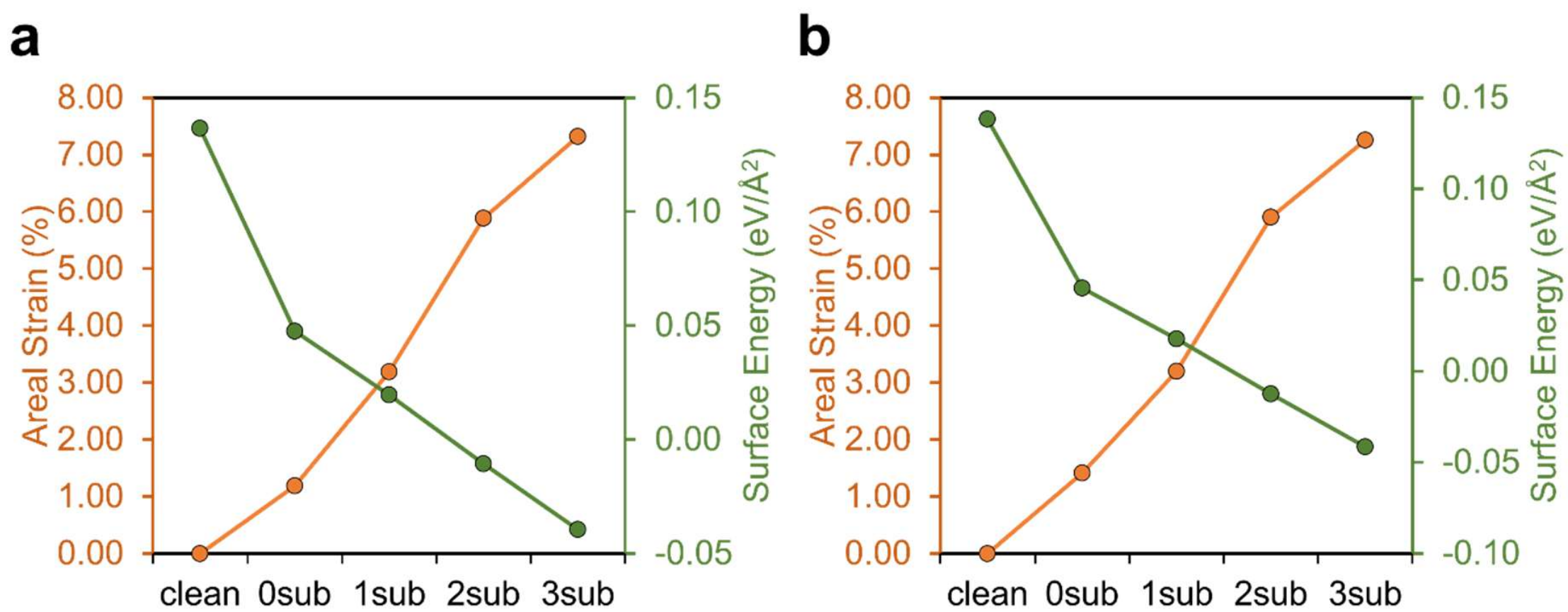

Figure S1. | Subsurface hydrogen alters the strain and stability of vacancy-containing Pd surfaces. Variation of areal strain (%) and surface energy (eV/Å²) of **(a)** Pd(111)-V_{L1} and **(b)** Pd(111)-V_{L2} as a function of increasing hydrogen subsurface concentrations (PdH_{0sub}, PdH_{1sub}, PdH_{2sub}, and PdH_{3sub}). Note "clean" here means the Pd(111)-$V_{L1/2}$ without H atoms on both surface and subsurface layers, while in all other cases, the surface is covered with 1.00 ML of H*. Source data are provided as a Source Data file.

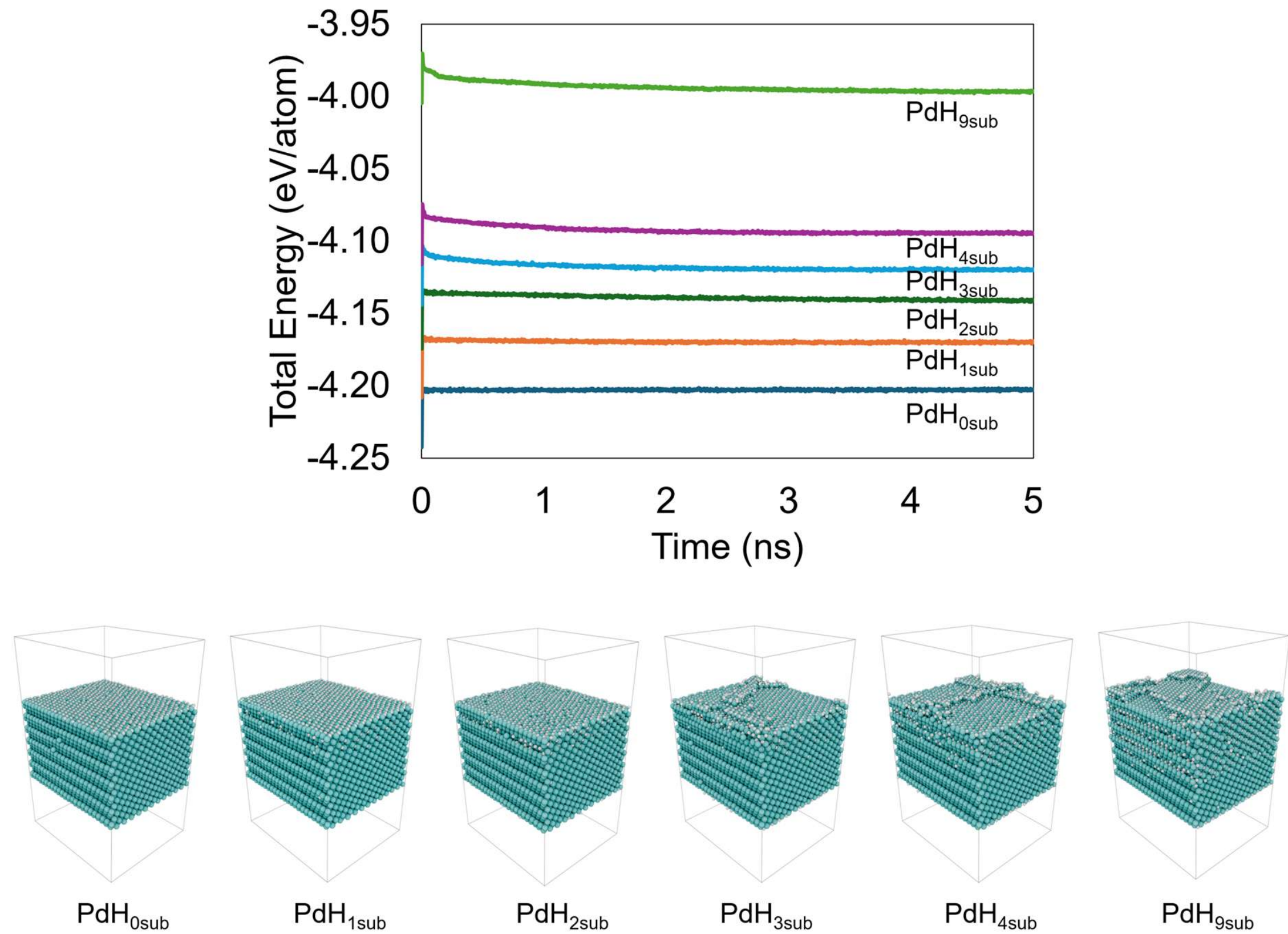

Figure S2. | Molecular dynamics equilibration modifies the structures of PdH_x/Pd(111) systems. (Top panel) Total energy versus time for PdH_x/Pd(111) during 5 ns MD simulations at 300 K, showing results for different hydrogen layer configurations. (Bottom panel) Reoptimized structures after 5 ns of MD equilibration. Source data are provided as a Source Data file.

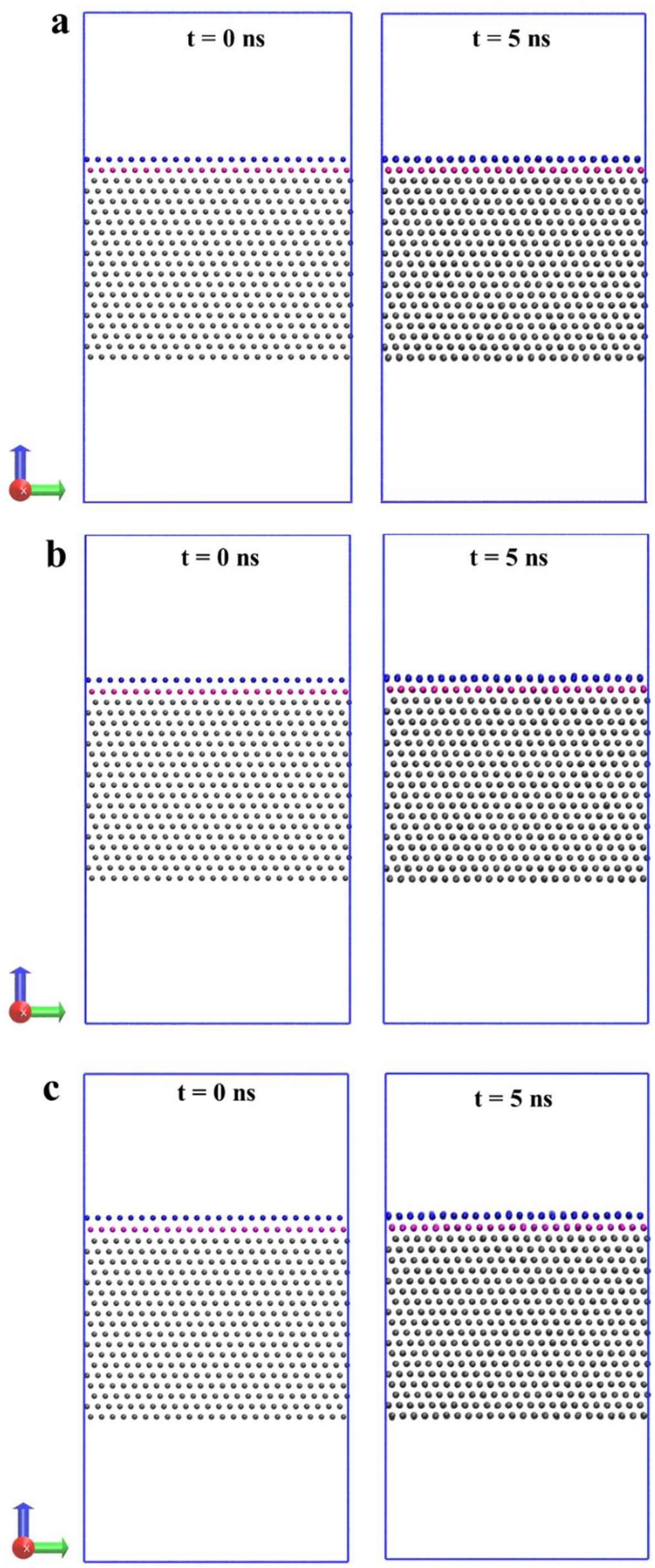

Figure S3. | Up to two subsurface hydride layers produce limited structural rearrangement. Color-coded Pd layers showing the (left panel) initial and (right panel) final structures obtained from the MD simulation for systems containing (a) 0, (b) 1, and (c) 2 H sublayers.

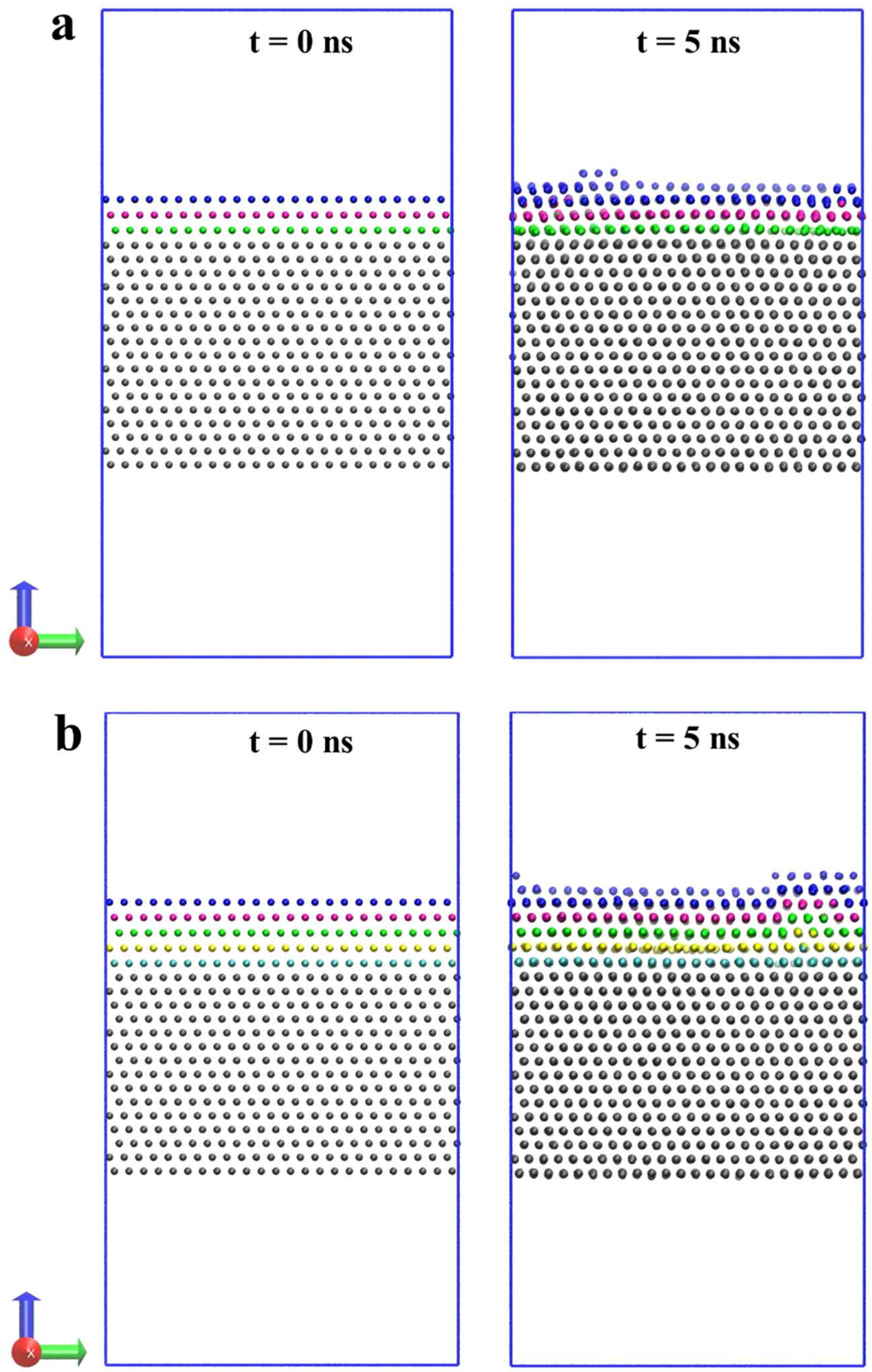

Figure S4. | Three and four subsurface hydride layers promote Pd surface restructuring. Color-coded Pd layers showing the (left panel) initial and (right panel) final structures obtained from the MD simulation for systems containing (a) 3, and (b) 4 H sublayers.

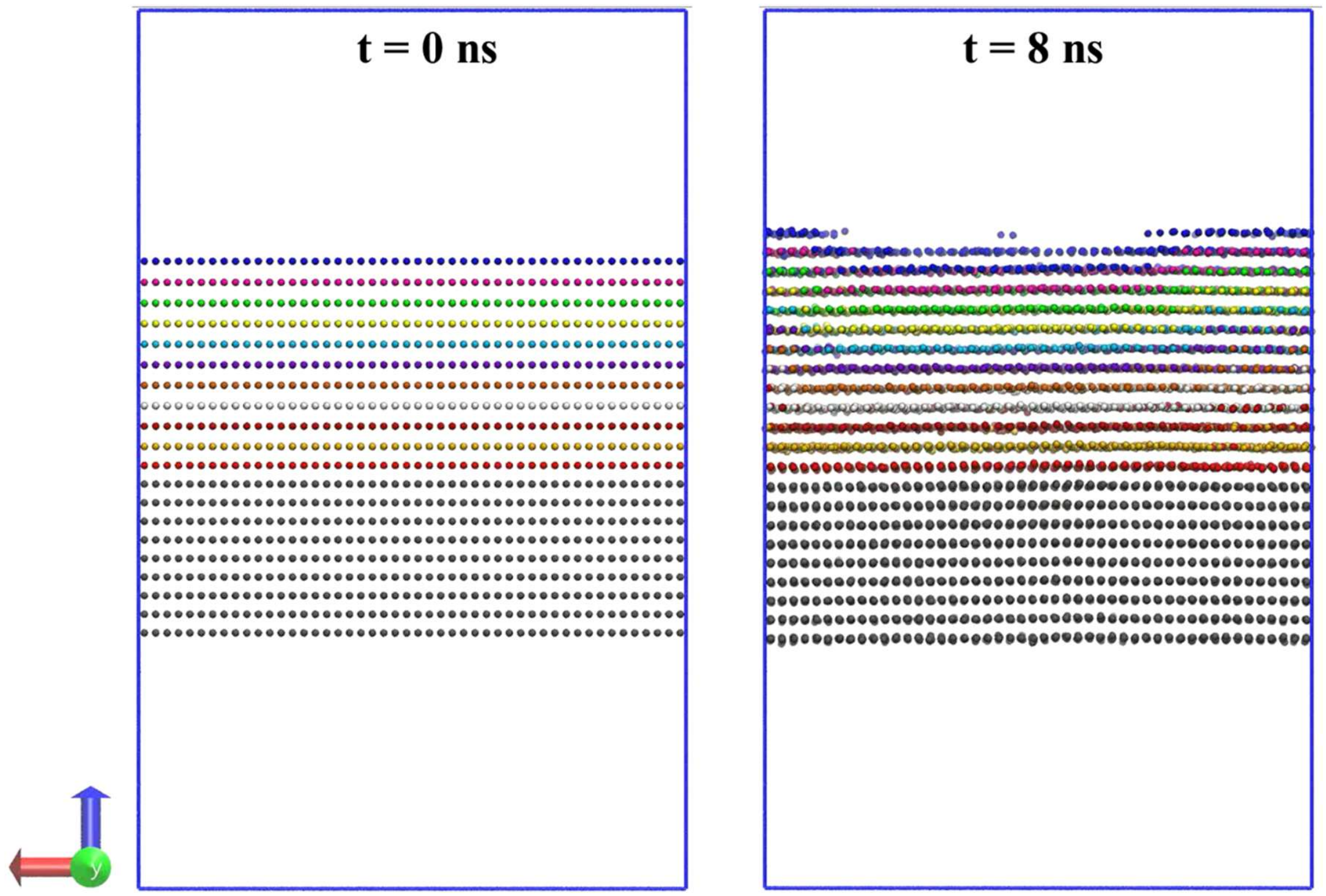

Figure S5. | Extensive Pd restructuring occurs at high subsurface hydride loading. Color-coded Pd layers showing the (left panel) initial and (right panel) final structures obtained from the MD simulation for systems containing 9 H sublayers.

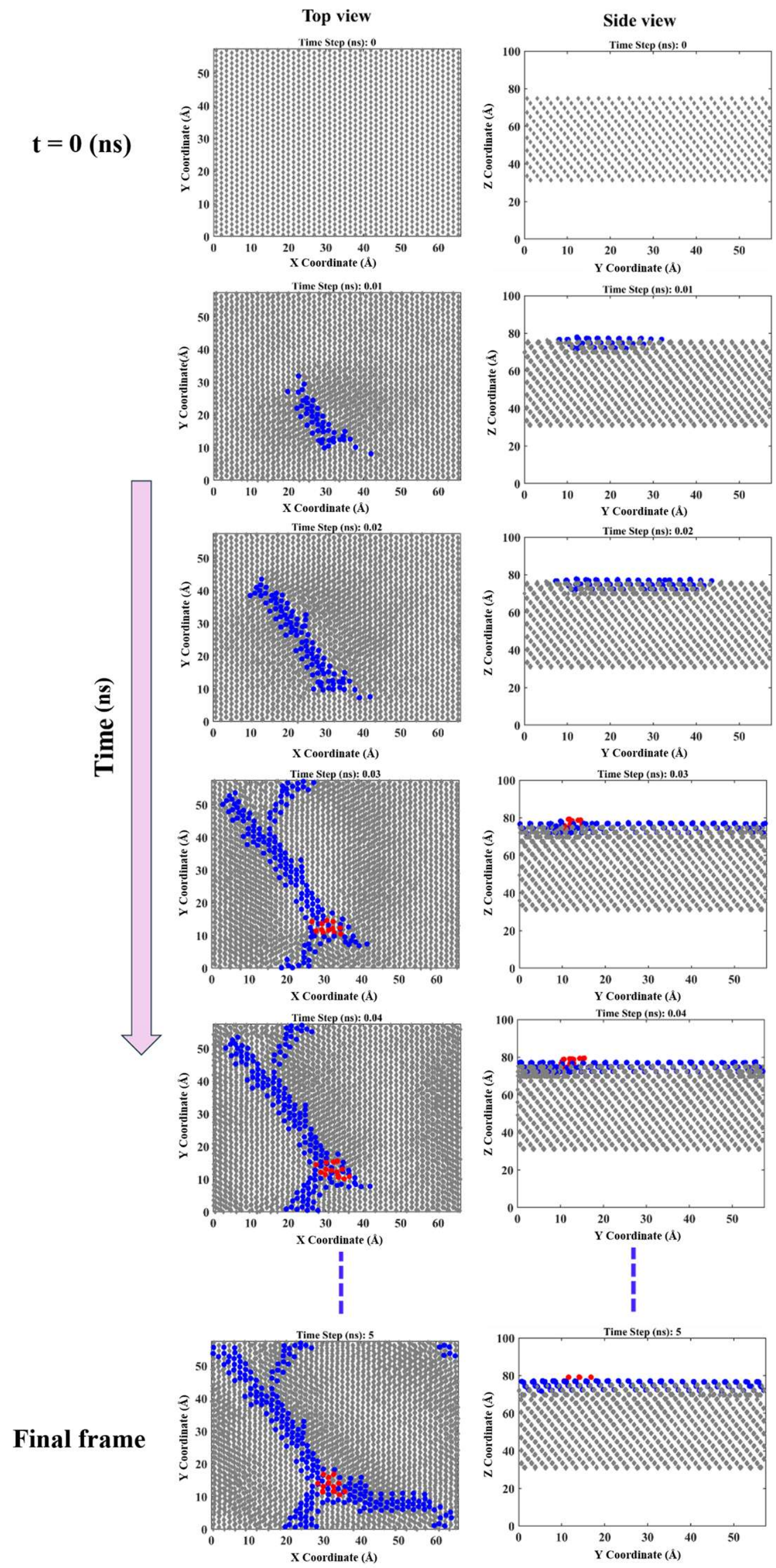

Figure S6. | Pd migration initiates in the system containing three subsurface hydride layers. Pd tracking analysis frames over time for PdH_{3sub}/Pd(111) from top and side views. Gray, blue, and red, represent Pd atoms that remain stable in their original layer (gray), move one layer upward (blue), and move two layers upward (red), respectively. For the corresponding H-tracking analysis, see Figure S13.

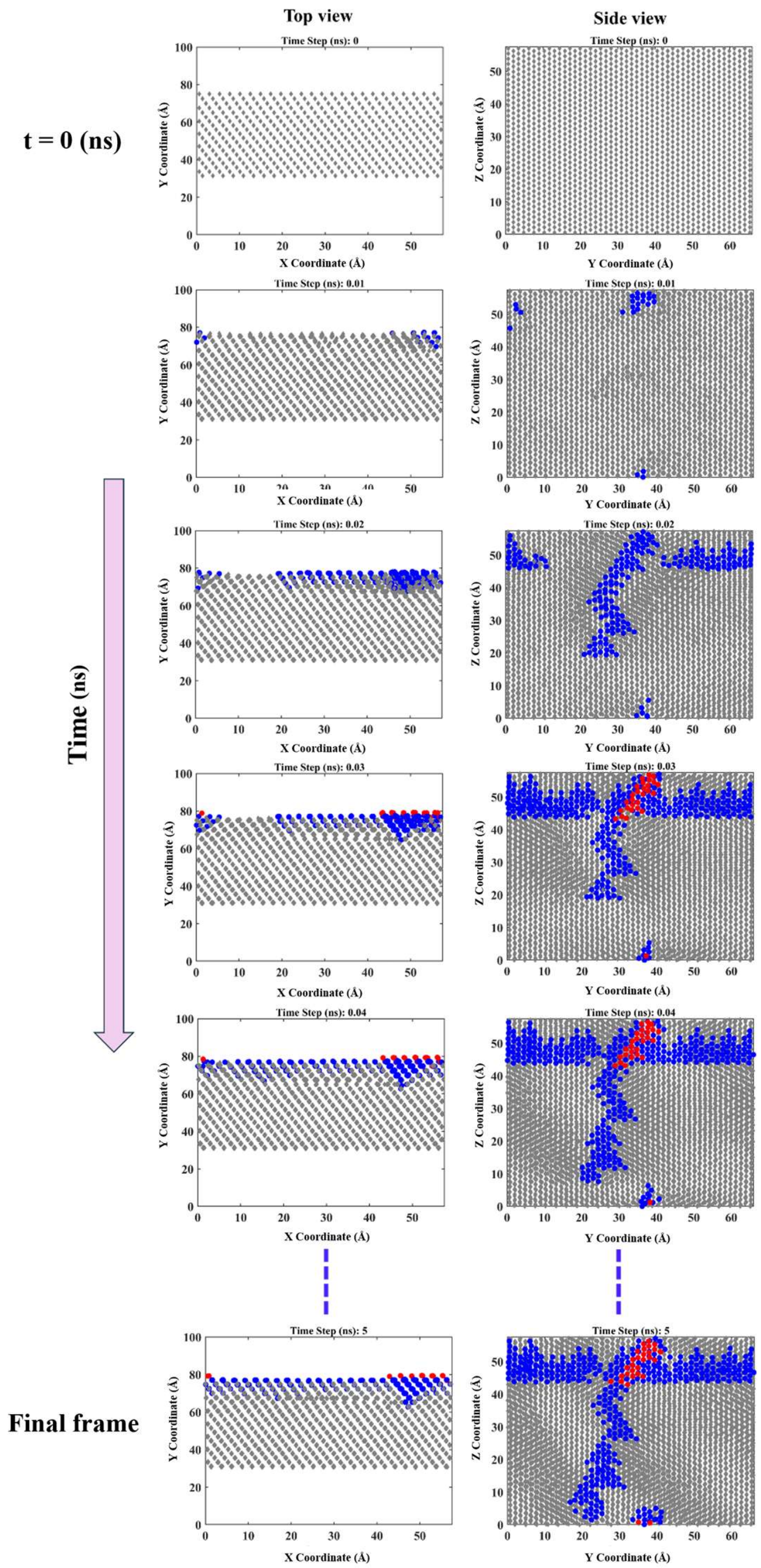

Figure S7. | Pd migration increases in the system containing four subsurface hydride layers. Pd tracking analysis frames over time for PdH_{4sub}/Pd(111) from side and top views. Gray, blue, and red, represent Pd atoms that remain stable in their original layer (gray), move one layer upward (blue), and move two layers upward (red), respectively. For the corresponding H-tracking analysis, see Figure S14.

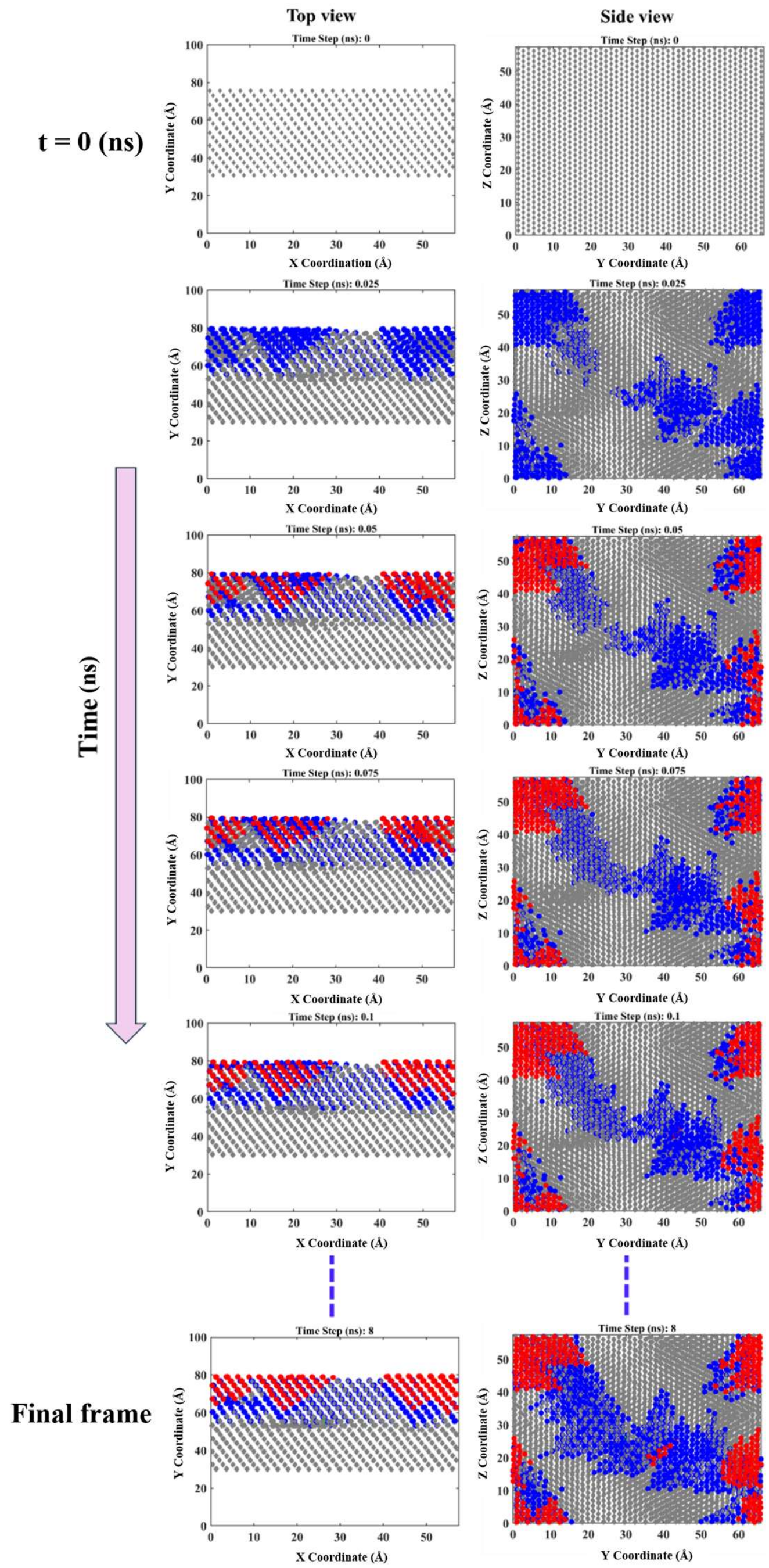

Figure S8. | Extensive Pd migration occurs in the system containing nine subsurface hydride layers. Pd tracking analysis frames over time for PdH_{9sub}/Pd(111) from side and top views. Gray, blue, and red, represent Pd atoms that remain stable in their original layer (gray), move one layer upward (blue), and move two layers upward (red), respectively. For the corresponding H-tracking analysis, see Figure S15.

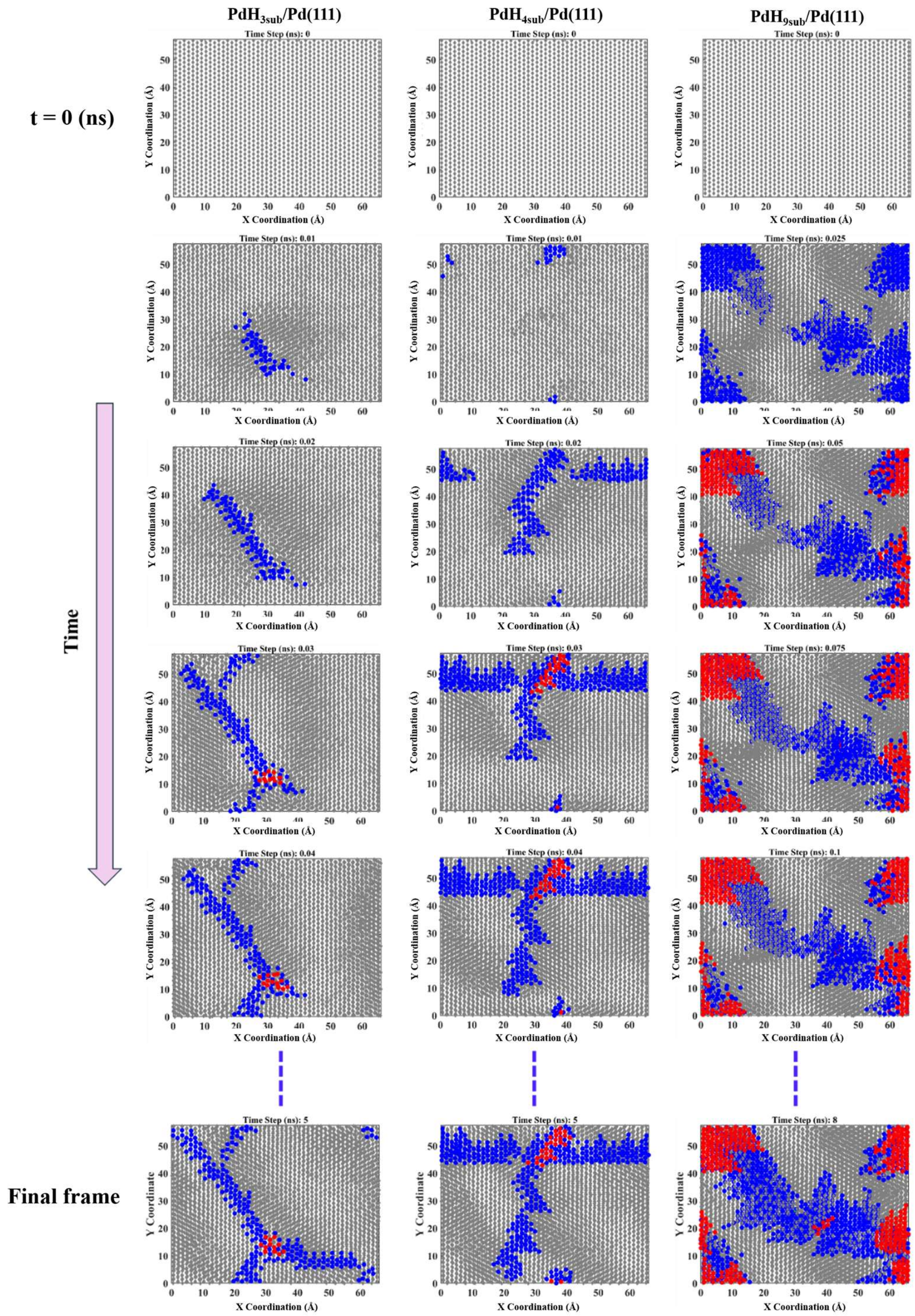

Figure S9. | Comparison of Pd migration during molecular dynamics equilibration. Pd tracking analysis frames over time for the three systems (PdH_{3sub}/Pd(111), PdH_{4sub}/Pd(111) and PdH_{9sub}/Pd(111)) compared, shows subsequently the emerging of Pd-mobility (PdH_{3sub}/Pd(111)), further increased Pd-mobility (PdH_{4sub}/Pd(111)) and extensive Pd-mobility (PdH_{9sub}/Pd(111)). Gray, blue, and red, represent Pd atoms that remain stable in their original layer (gray), move one layer upward (blue), and move two layers upward (red), respectively. For the corresponding H-tracking analysis, see Figures S13-S15.

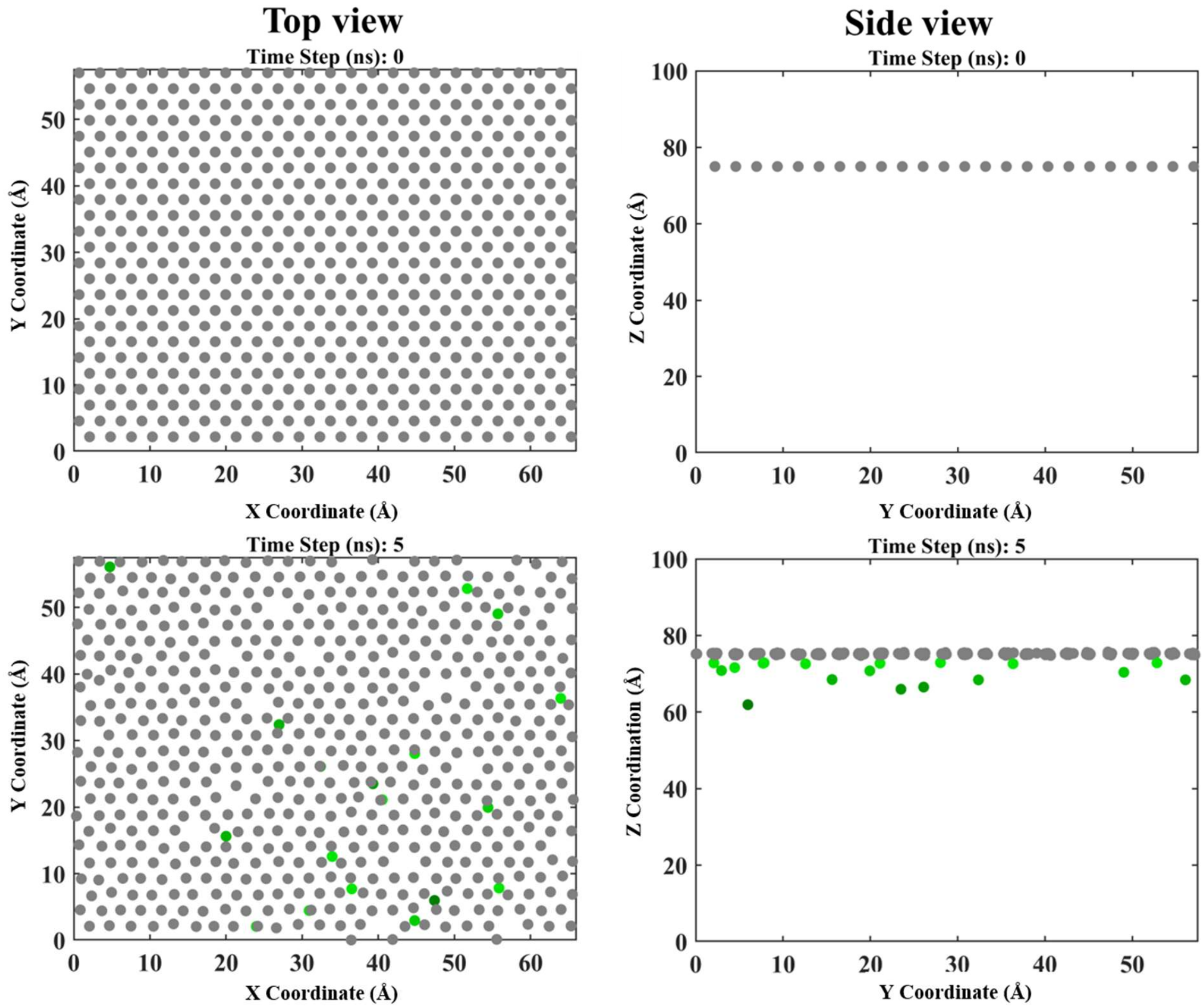

Figure S10. | H-tracking analysis for the system with one monolayer surface hydrogen and no subsurface hydrogen, in which Pd atoms remain largely within their initial layers. The first and last frames obtained from H tracking analysis for PdH_{0sub}/Pd(111) from top and side views. Gray, blue, red, green, and dark green represent Pd atoms that remain stable in their original layer (gray), move one layer upward (blue), move two layers upward (red), and move one layer downward (green) respectively. Larger upward and downward migrations are represented by progressively lighter shades of red and progressively darker shades of green, respectively. After 5 ns MD equilibration at 298 K, there is clearly hydrogen absorption in subsurface layers.

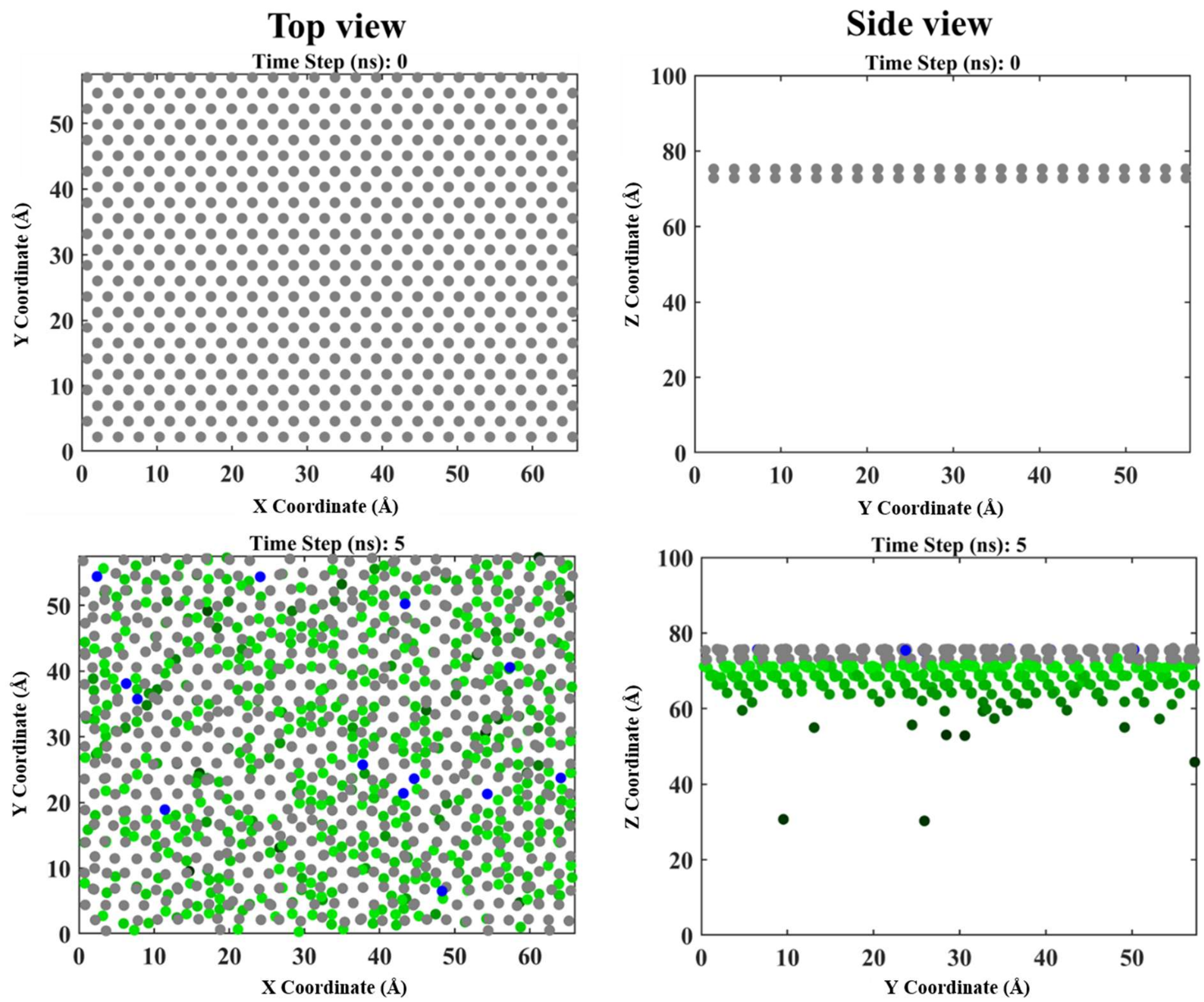

Figure S11. | H-tracking analysis for the system with one monolayer surface hydrogen and one subsurface hydrogen layer, showing limited Pd movement. First and last frames obtained from H tracking analysis for $PdH_{1sub}/Pd(111)$ from top and side views. Gray, blue, red, green, and dark green represent Pd atoms that remain stable in their original layer (gray), move one layer upward (blue), move two layers upward (red), and move one layer downward (green) respectively. Larger upward and downward migrations are represented by progressively lighter shades of red and progressively darker shades of green, respectively. After 5 ns MD equilibration at 298 K, there is clearly hydrogen absorption in subsurface layers.

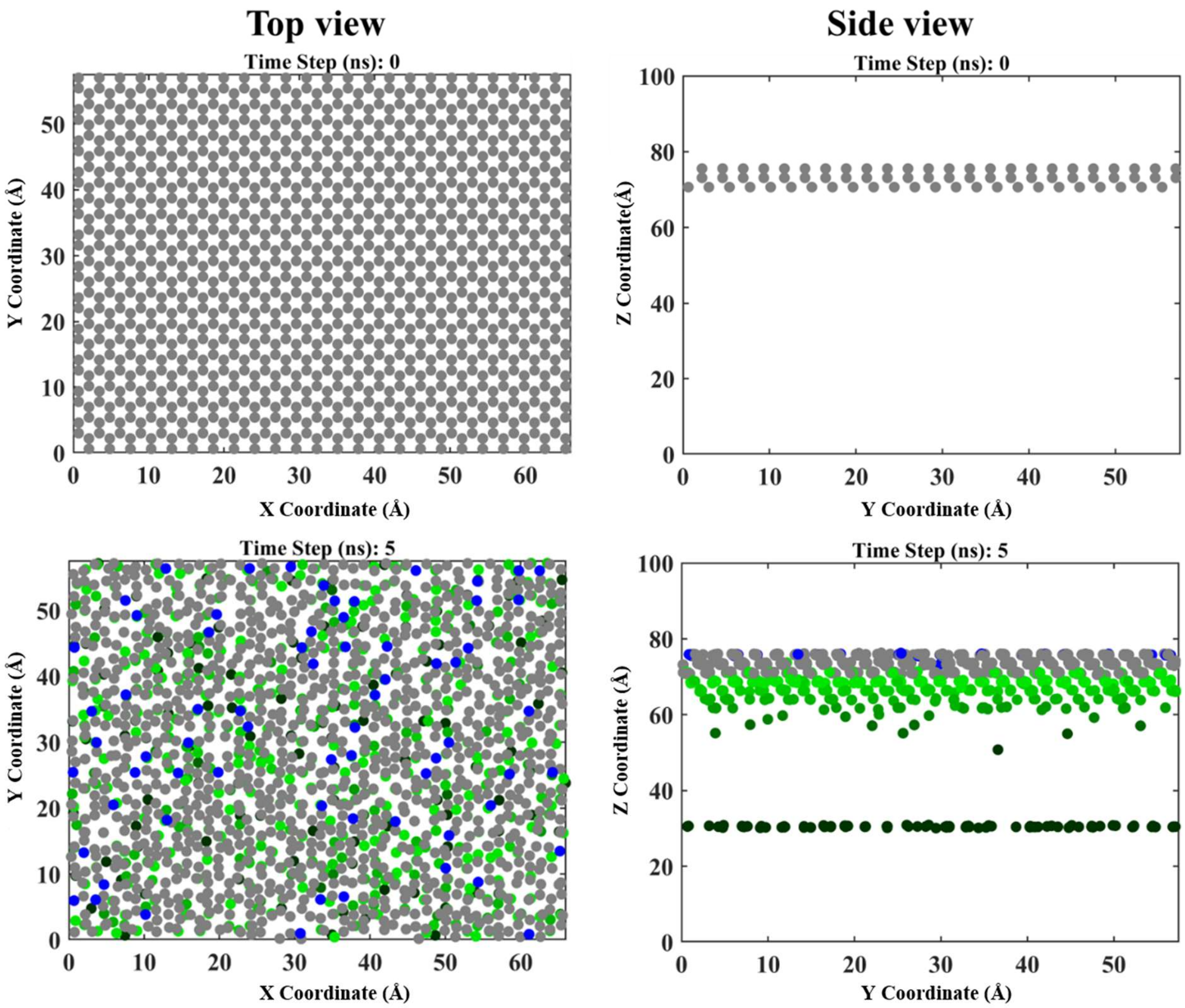

Figure S12. | H-tracking analysis for the system with one monolayer surface hydrogen and two subsurface hydrogen layers, showing limited Pd movement occurs. First and last frames obtained from H tracking analysis for PdH_{2sub}/Pd(111) from top and side views. Gray, blue, red, green, and dark green represent Pd atoms that remain stable in their original layer (gray), move one layer upward (blue), move two layers upward (red), and move one layer downward (green) respectively. Larger upward and downward migrations are represented by progressively lighter shades of red and progressively darker shades of green, respectively. After 5 ns MD equilibration at 298 K, there is clearly hydrogen absorption in subsurface layers, and some of the hydrogens even reach the opposite side of the slab after diffusing ~ 40 Å deeper.

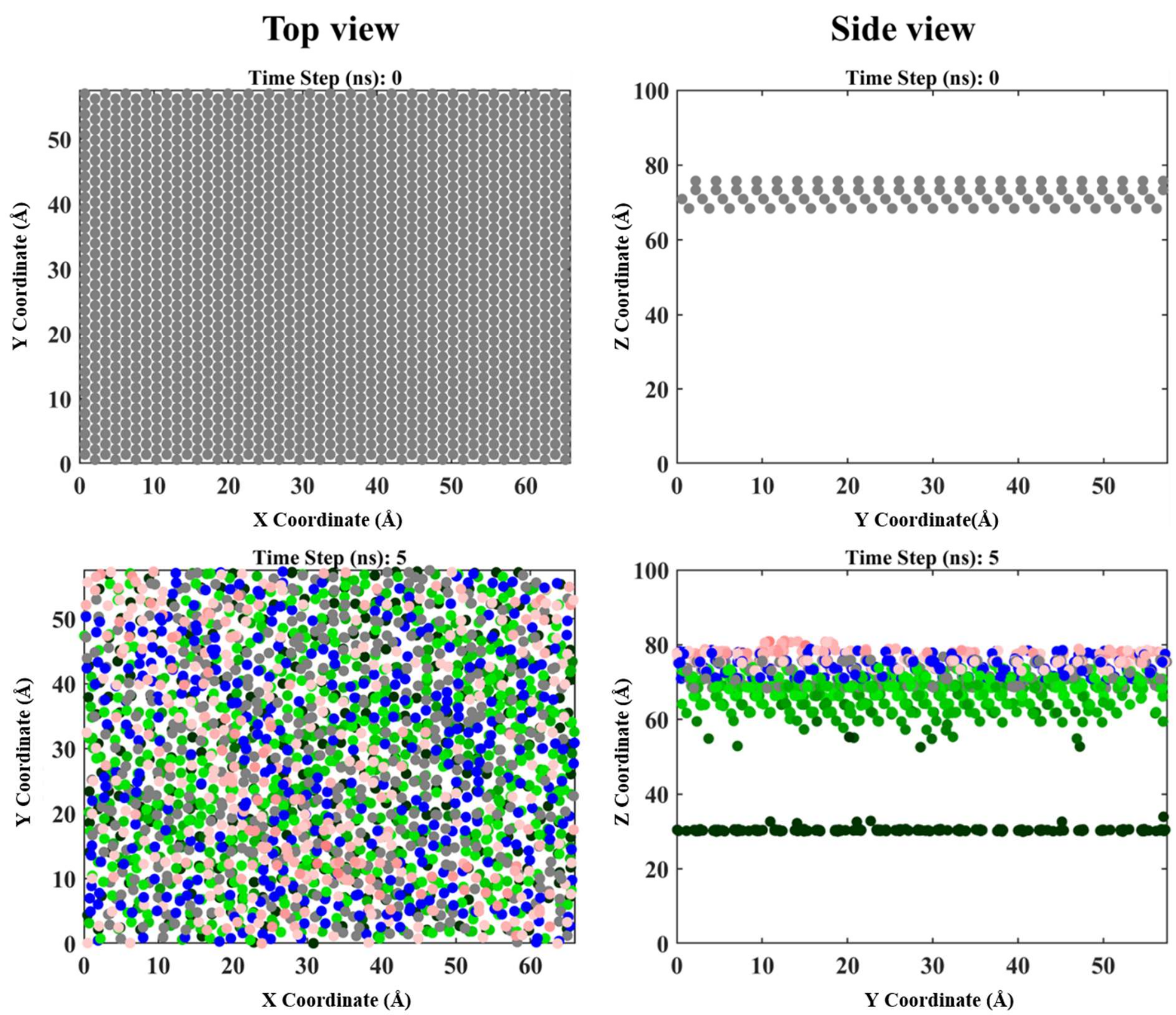

Figure S13. | H-tracking analysis for the system with one monolayer surface hydrogen and three subsurface hydrogen layers, showing the emergence of Pd migration. The first and last frames obtained from H tracking analysis for PdH_{3sub}/Pd(111) from top and side views. Gray, blue, red, green, and dark green represent Pd atoms that remain stable in their original layer (gray), move one layer upward (blue), move two layers upward (red), and move one layer downward (green) respectively. Larger upward and downward migrations are represented by progressively lighter shades of red and progressively darker shades of green, respectively.

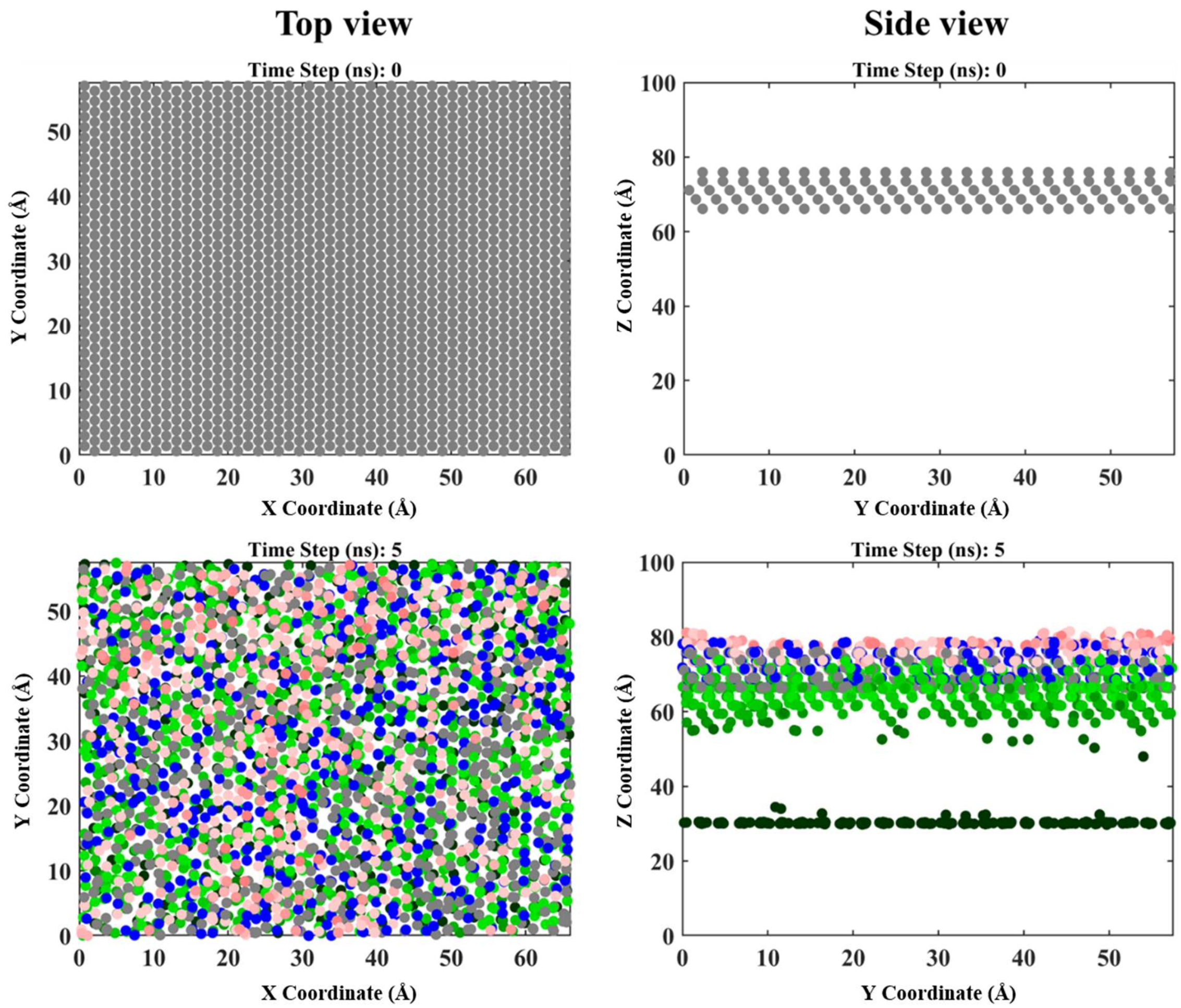

Figure S14. | H-tracking analysis for the system with one monolayer surface hydrogen and four subsurface hydrogen layers, showing increased Pd migration increases. The first and last frames obtained from H tracking analysis for PdH_{4sub}/Pd(111) from top and side views. Gray, blue, red, green, and dark green represent Pd atoms that remain stable in their original layer (gray), move one layer upward (blue), move two layers upward (red), and move one layer downward (green) respectively. Larger upward and downward migrations are represented by progressively lighter shades of red and progressively darker shades of green, respectively.

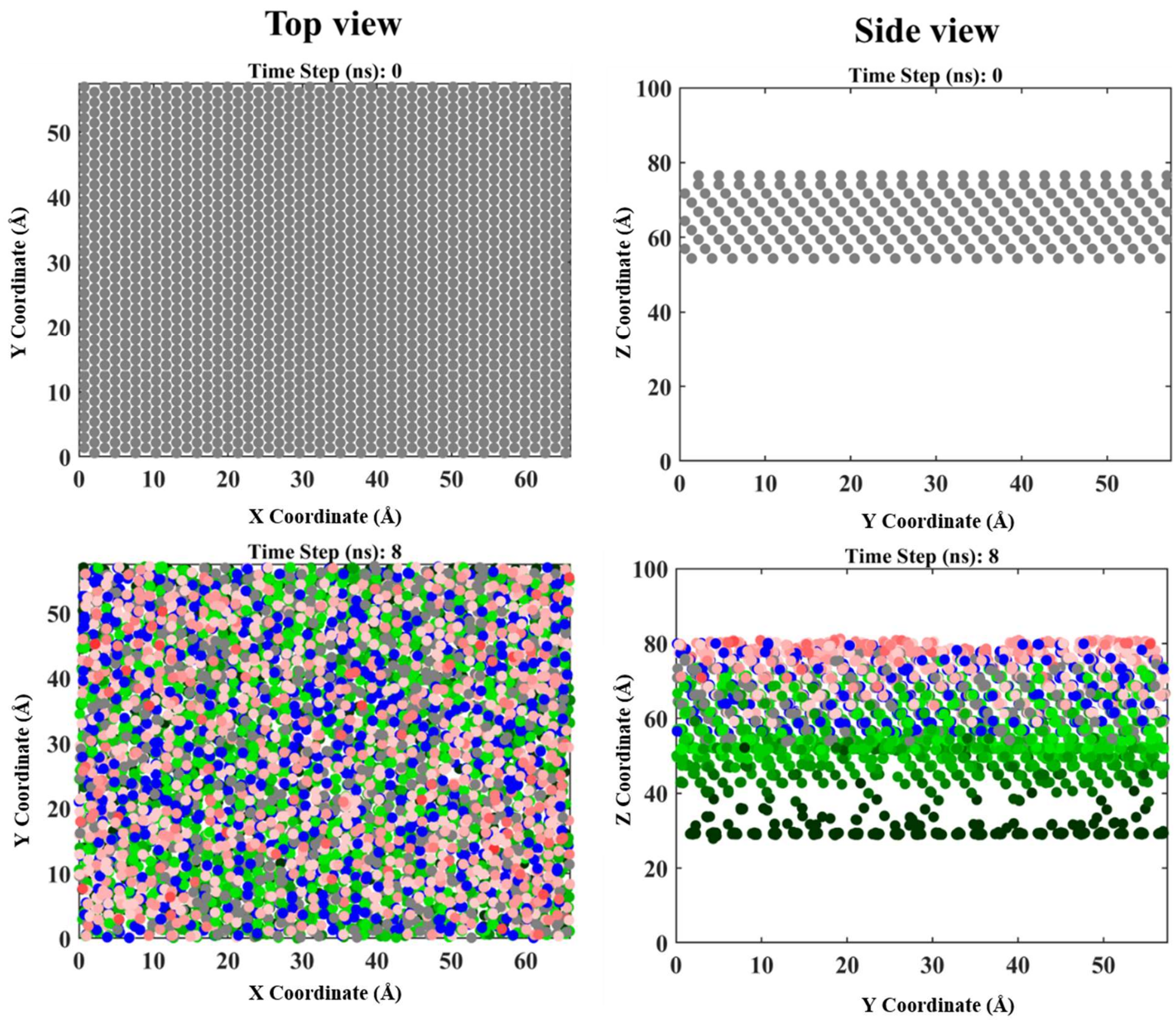

Figure S15. | H-tracking analysis for the system with one monolayer surface hydrogen and nine subsurface hydrogen layers. The first and last frames obtained from H tracking analysis for PdH_{9sub}/Pd(111) from top and side views. Gray, blue, red, green, and dark green represent Pd atoms that remain stable in their original layer (gray), move one layer upward (blue), move two layers upward (red), and move one layer downward (green) respectively. Larger upward and downward migrations are represented by progressively lighter shades of red and progressively darker shades of green, respectively.

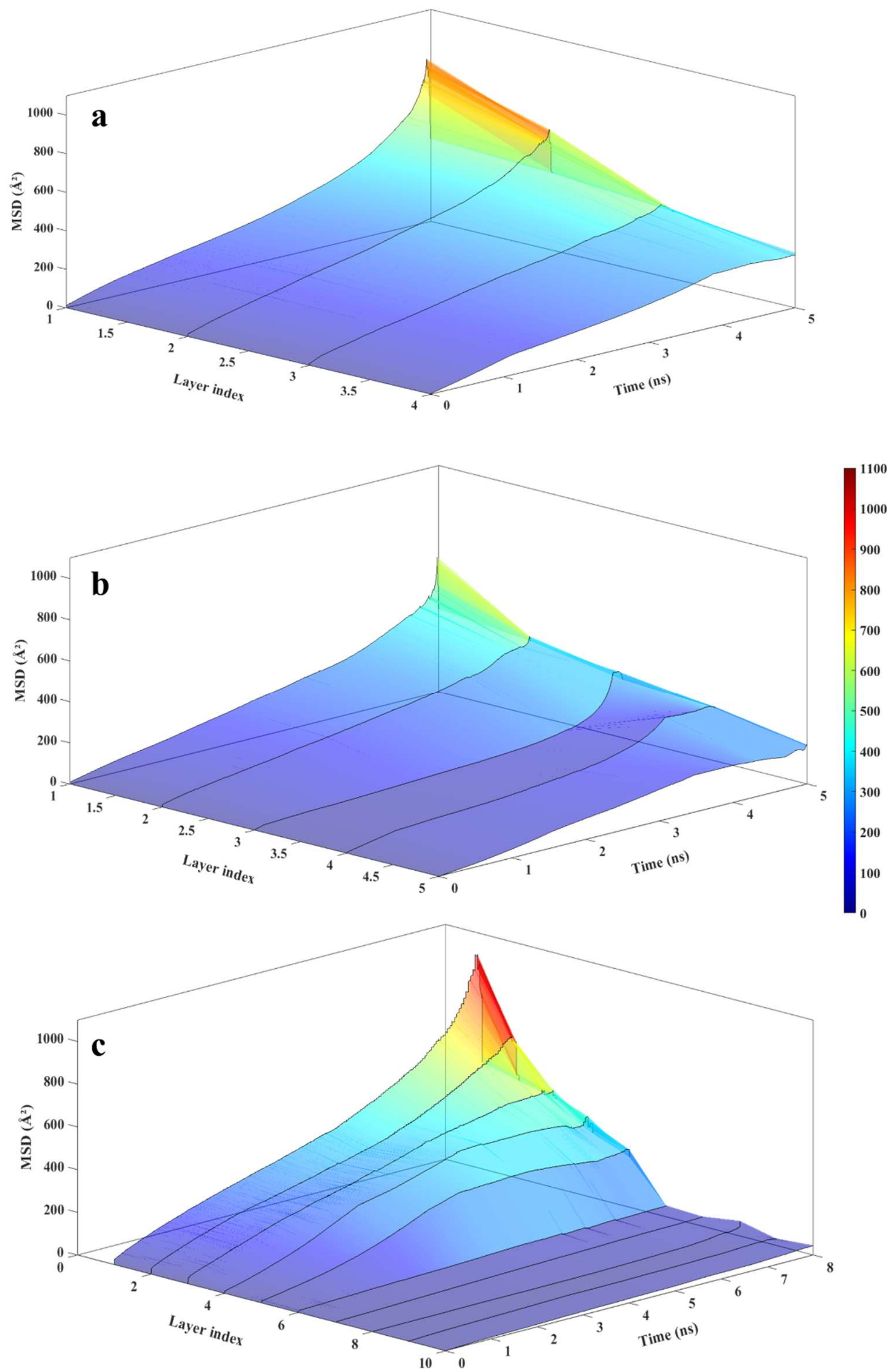

Figure S16. | Hydrogen mobility increases with subsurface hydride loading. Mean square displacement (MSD) in the z direction for each layer of H for (a) PdH_{3sub}/Pd(111), (b) PdH_{4sub}/Pd(111), and (c) PdH_{9sub}/Pd(111). Source data are provided as a Source Data file.

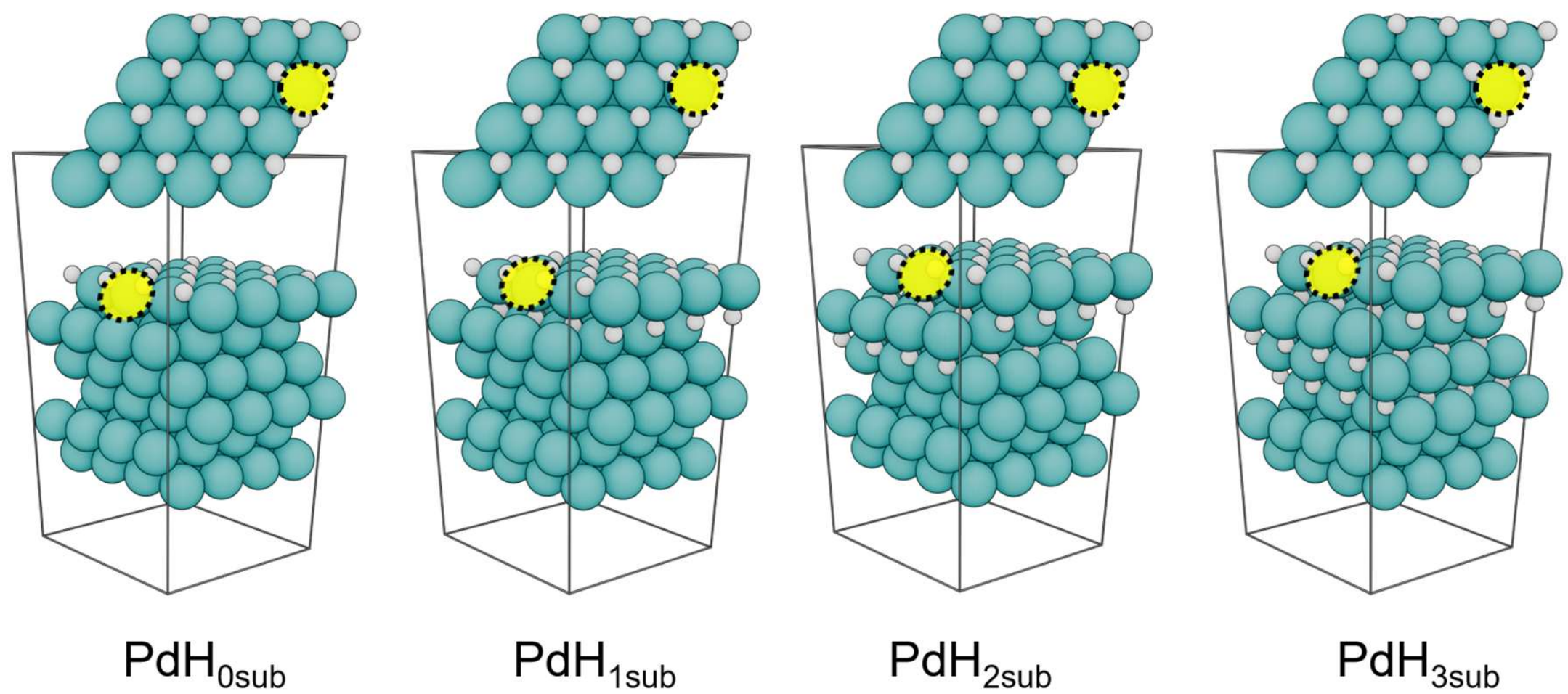

Figure S17. | Atomic structures of vacancy-containing Pd surfaces at different subsurface hydrogen concentrations. Top and perspective views of 1ML H-covered Pd(111) surfaces at different hydrogen subsurface concentrations (PdH_{0sub}, PdH_{1sub}, PdH_{2sub}, PdH_{3sub}), with vacancy sites highlighted by yellow circles.

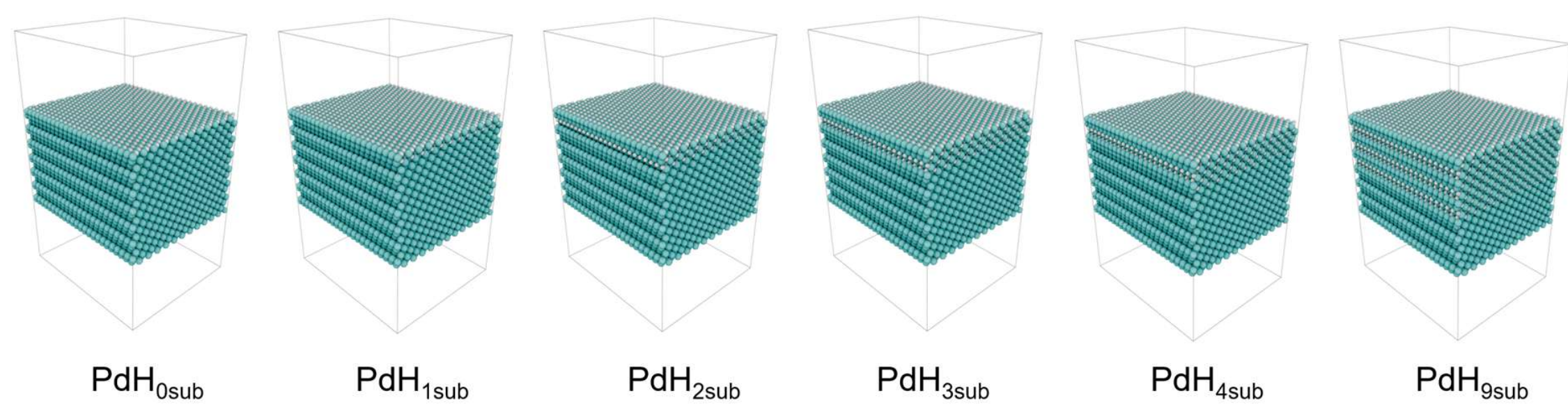

Figure S18. | Initial PdH_x/Pd(111) models spanning a range of subsurface hydride loadings. Initial models before molecular dynamics simulations of PdH_x/Pd(111) systems with varying numbers of hydride sublayers (0, 1, 2, 3, 4, and 9 H sublayers). The aqua and white spheres represent Pd and H atoms, respectively.

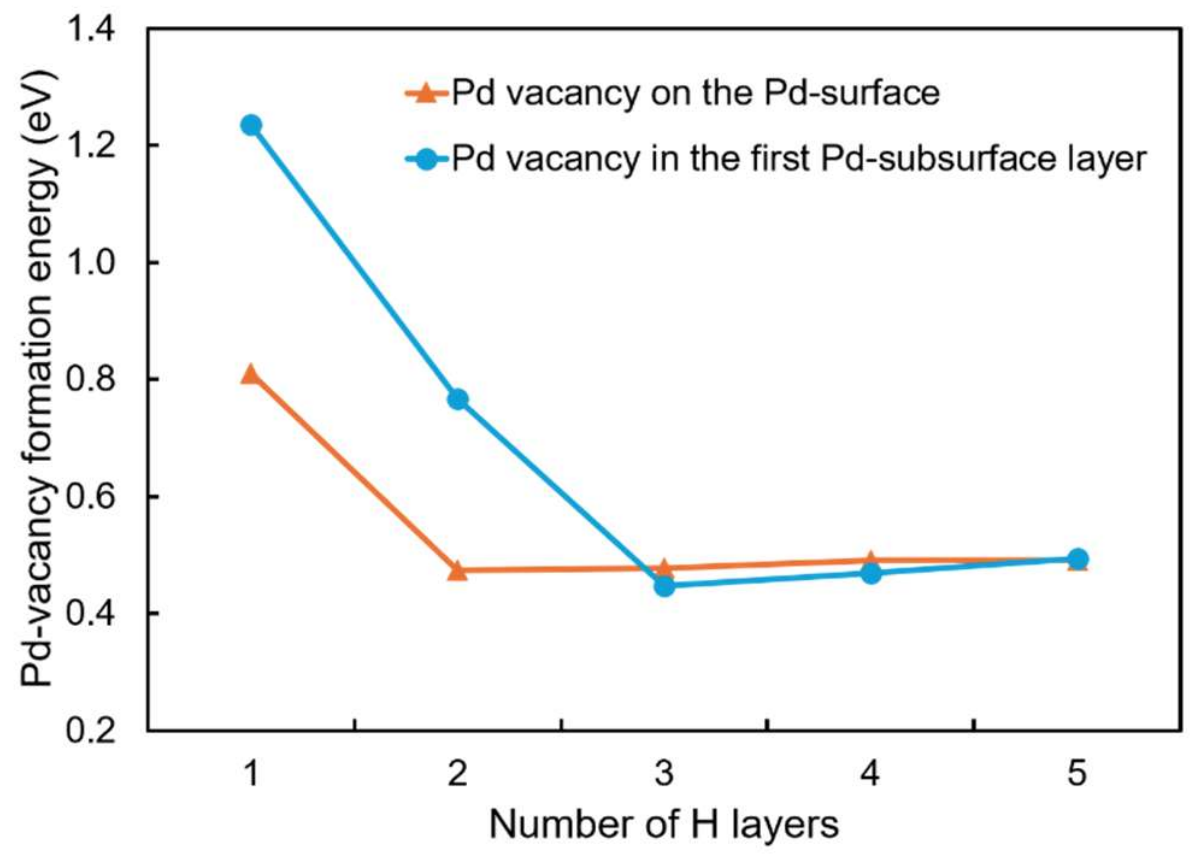

Figure S19. | Subsurface hydrogen modifies Pd-vacancy formation energies. The Pd-vacancy formation energies calculated using the ACE-PBE-D3 potential for structures optimized prior to MD simulations. Vacancies were introduced in the surface Pd layer and the first subsurface Pd layer of PdH_x/Pd(111), and the corresponding formation energies are presented as a function of the number of subsurface hydrogen layers. Source data are provided as a Source Data file.

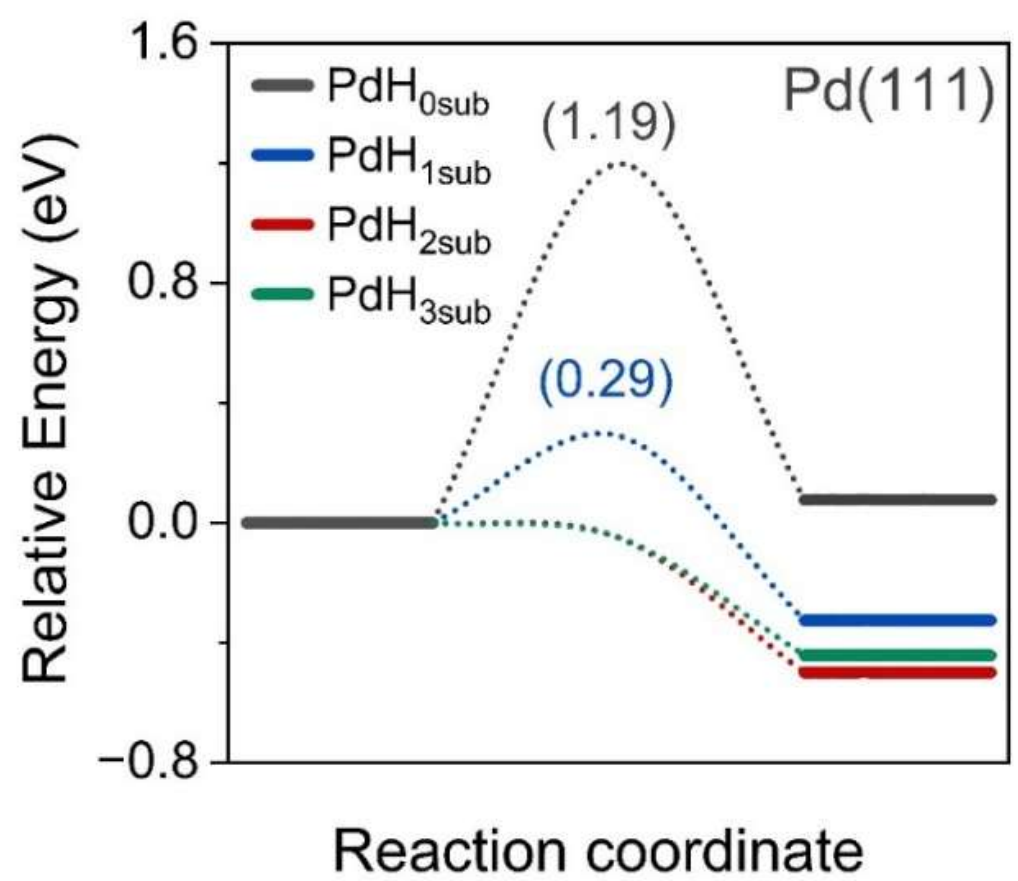

Figure S20. | Subsurface hydrogen promotes Pd migration. Reaction energy profiles for Pd migration on Pd(111) surfaces calculated using the PBE functional without the D3(BJ) dispersion correction. For the corresponding profiles with D3(BJ), see Figure 1d. The corresponding migration barriers for different hydrogen subsurface concentrations (PdH_{0sub}, PdH_{1sub}, PdH_{2sub}, PdH_{3sub}) are shown relative to the initial state as defined in Figure 1d. All structures were relaxed to allow in-plane stress relaxation while keeping the cell shape and volume fixed. The results show the ease of vacancy formation in the sublayer as a function of the number of hydride sublayers and their stability under reaction conditions. Source data are provided as a Source Data file.

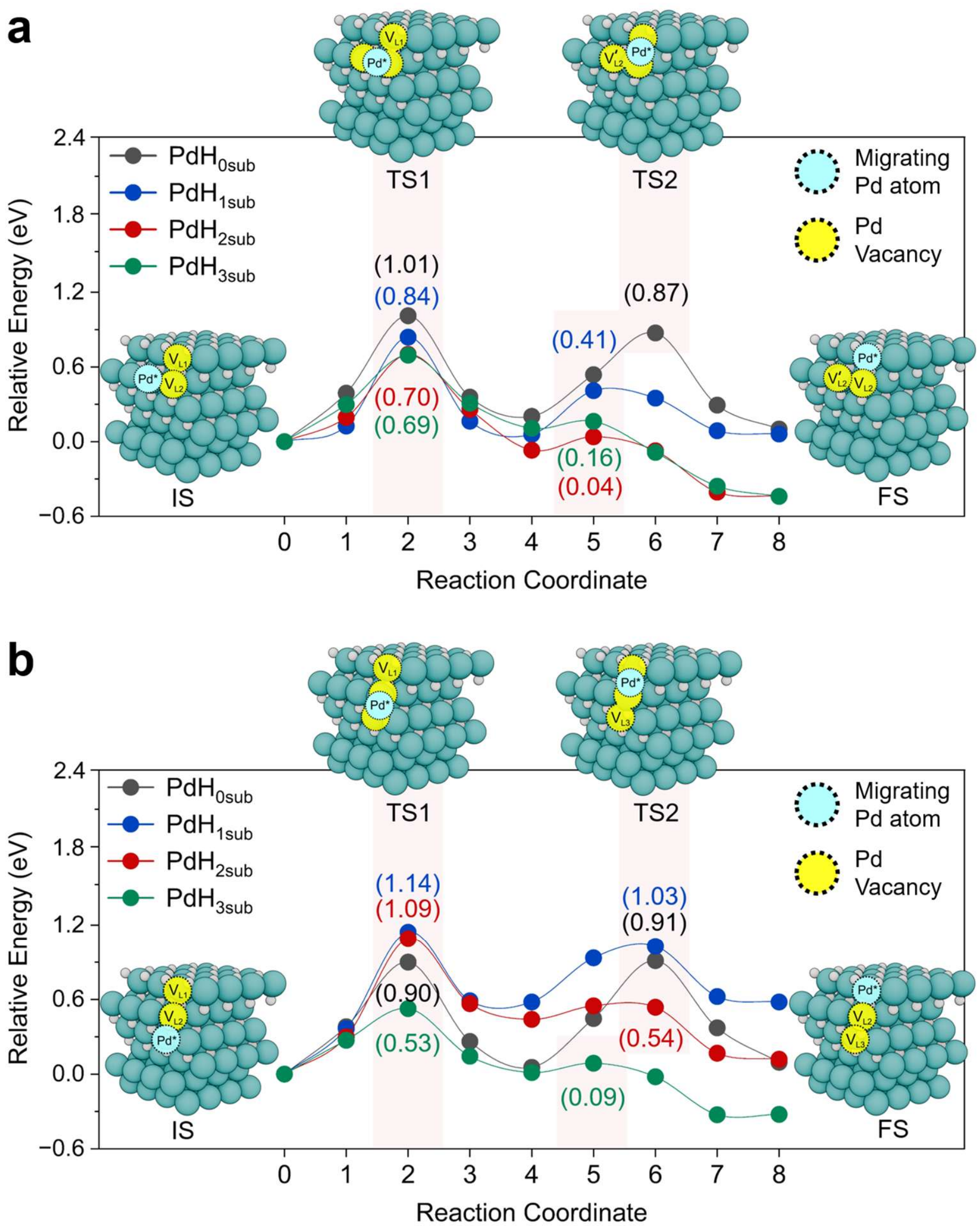

Figure S21. Subsurface hydrogen lowers the barriers for Pd migration. Reaction energy profiles for Pd migration on Pd(111) surfaces with a double-vacancy configuration calculated using the PBE functional without the D3(BJ) dispersion correction. The profiles show the relative energies of the initial, intermediate, and final states, along with the corresponding migration barriers for different subsurface hydrogen concentrations (PdH_{0sub}, PdH_{1sub}, PdH_{2sub}, PdH_{3sub}). Two distinct pathways are considered: **(a)** Route 1 involves lateral migration within the second layer (L2) followed by upward diffusion into the surface vacancy (L1), while **(b)** Route 2 proceeds via a deeper-layer exchange mechanism (L3 → L2 → L1). The first migration process is rate-determining (the highest barrier), and the energy levels relative to the starting position are indicated. Source data are provided as a Source Data file.

Table S1. Calculated work functions (Φ, eV) for the configurations along vacancy-mediated Pd self-diffusion pathways under different subsurface hydrogen loadings. The initial state corresponds to a surface vacancy, while the final state corresponds to a subsurface vacancy (V_{L2}/V_{L3}). In case of double vacancies, two diffusion pathways were considered: Route 1 (R1) and Route 2 (R2), here Route 1 involves lateral migration within the second layer (L2) followed by upward diffusion into the surface vacancy (in L1), while **(b)** Route 2 proceeds via a deeper-layer exchange mechanism (L3 → L2 → L1).

Vacancy Type	No of H sublayers	$\Phi_{initial}$	Φ_{final}	ΔΦ
**Single**	0	6.28	5.66	-0.62
**Single**	1	5.77	5.66	-0.11
**Single**	2	5.71	5.69	-0.02
**Double-R_1**	0	5.63	5.94	0.31
**Double-R_2**	0	5.63	5.73	0.10
**Double-R_1**	1	5.79	5.70	-0.09
**Double-R_2**	1	5.79	5.70	-0.09
**Double-R_1**	2	5.72	5.95	0.23
**Double-R_2**	2	5.72	5.75	0.03
**Double-R_1**	3	5.70	5.72	0.02
**Double-R_2**	3	5.70	5.90	0.20

Validation of the Atomic Cluster Expansion (ACE-PBE-D3) Potential

The large-scale molecular dynamics simulations presented in this work were performed using an Atomic Cluster Expansion potential trained on a PBE-D3 reference dataset (ACE-PBE-D3). A comprehensive description of the potential development, training procedure, and benchmarking is available as a preprint[4] and in data repository[5]. Here, we summarize the key validation results most relevant to the present investigation of hydride-induced Pd reconstruction.

Training Dataset

The ACE-PBE-D3 potential was trained using a diverse dataset of Pd–H configurations generated from density functional theory calculations. The dataset includes bulk Pd and PdH structures spanning a wide range of hydrogen concentrations, surface slabs, strained configurations, thermally perturbed structures, and hydrogen-containing environments relevant to hydriding and surface reconstruction. To ensure robust transferability, both low-energy equilibrium structures and higher-energy distorted configurations were included.

All structures within 2 eV atom^{-1} above the ground-state convex hull represent approximately 99% of the reference dataset, while a smaller subset of higher-energy configurations was retained to improve the description of short-range repulsive interactions.

Energy and Force Accuracy

The final ACE-PBE-D3 model achieves an energy root-mean-square error (RMSE) of 9 meV atom^{-1} for low-energy structures and 39 meV atom^{-1} across the complete dataset. Corresponding force RMSE values are 65 meV Å^{-1} for low-energy structures and 97 meV Å^{-1} for the full dataset. A 10% hold-out test set, excluded entirely from training, was used for validation. Figure S22(a-b) shows cross-validation plots for predicted energies and forces, demonstrating excellent agreement between ACE predictions and reference DFT calculations.

Structural, Elastic and Vibrational Properties

To assess the transferability of the potential, equilibrium properties of Pd and PdH crystals were compared against DFT calculations. Figure S22c shows the energy–volume curves for fcc-Pd, $PdH_{0.5}$, and stoichiometric PdH obtained using ACE and DFT. Excellent agreement is observed across the entire volume range considered. Figure S22d compares the equilibrium lattice constants and bulk moduli predicted by ACE with DFT and available experimental measurements. The close agreement confirms that the potential accurately reproduces the structural response of Pd and PdH phases, which is particularly important for describing hydride-induced lattice expansion and strain accumulation. Because lattice dynamics play an important role in hydriding phenomena, phonon dispersions were also evaluated. Figure S22e,f compares phonon band structures obtained using ACE and DFT for fcc-Pd and stoichiometric PdH, respectively. The ACE potential accurately reproduces the principal vibrational features of both systems and captures the essential phonon characteristics associated with hydrogen incorporation.

The present work focuses on hydride-induced Pd migration, strain relaxation, and surface reconstruction. These phenomena depend critically on the accurate description of: (i) Pd–Pd and Pd–H interactions, (ii) lattice expansion associated with hydriding, (iii) elastic response and strain accumulation, and (iv) finite-temperature atomic dynamics.

The excellent agreement between ACE and DFT for structural, elastic, energetic, and vibrational properties provides confidence that the ACE-PBE-D3 potential is sufficiently

accurate and transferable for investigating the hydrided PdH_x/Pd(111) systems considered in this study.

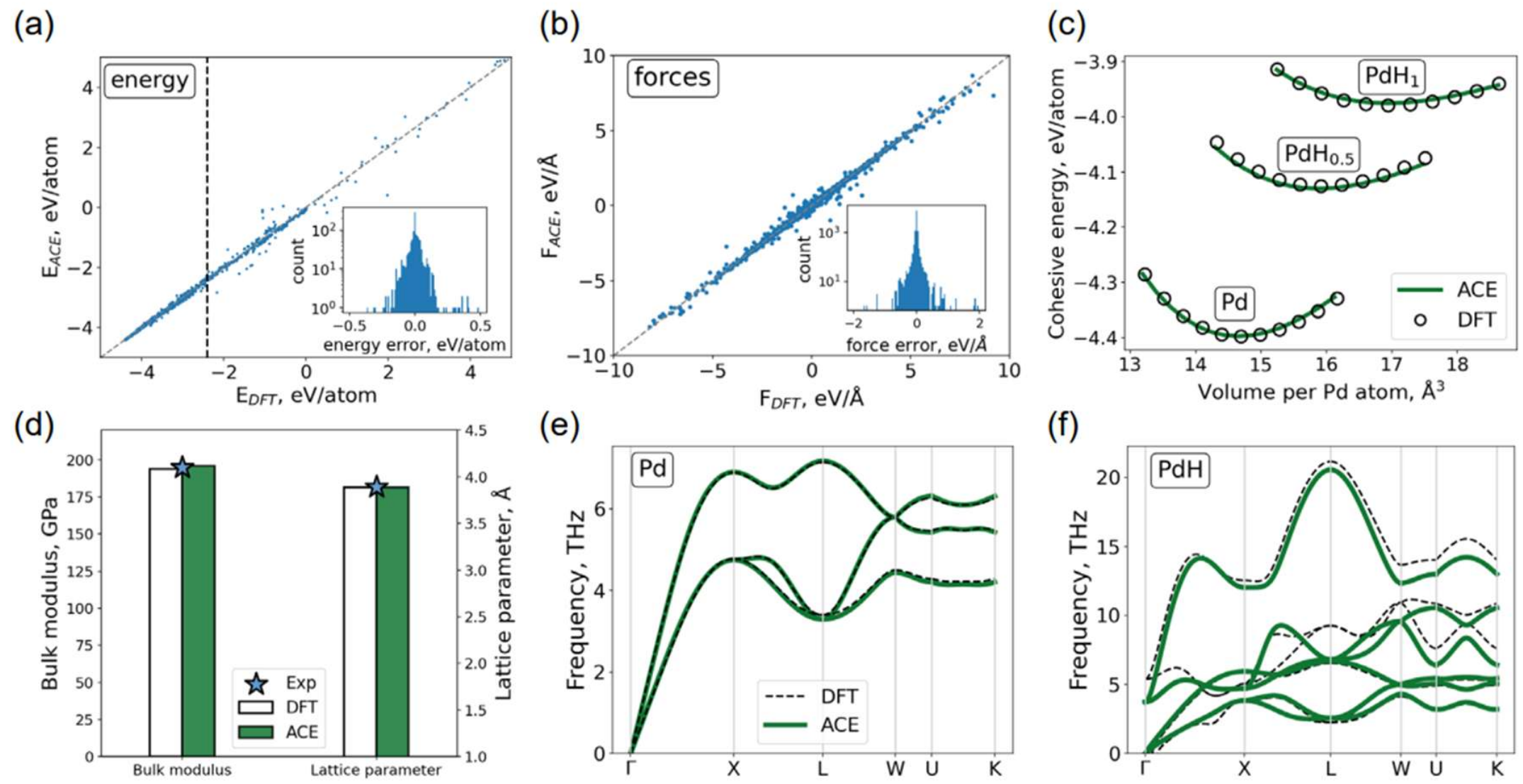

Figure S22. Validation of the ACE-PBE-D3 potential. (a) Cross-validation parity plot for predicted energies relative to reference DFT calculations, with the inset showing the corresponding error distribution. The vertical dashed line denotes the energy cutoff below which approximately 99% of the structures in the dataset are located. (b) Cross-validation parity plot for predicted forces, with the inset showing the force error distribution. (c) Energy–volume curves of fcc-Pd, $PdH_{0.5}$, and stoichiometric PdH predicted by ACE-PBE-D3 and DFT, demonstrating excellent agreement across a range of lattice volumes. (d) Comparison of equilibrium lattice constants and bulk moduli obtained using ACE-PBE-D3, DFT, and available experimental data, confirming accurate reproduction of the structural and elastic properties of Pd and PdH phases. (e,f) Phonon band structures of fcc-Pd and stoichiometric PdH, respectively, calculated using ACE-PBE-D3 and DFT, showing that the potential accurately captures the vibrational properties relevant to hydriding phenomena. Collectively, these results demonstrate the accuracy and transferability of the ACE-PBE-D3 potential for describing the structural, elastic, energetic, and vibrational properties of Pd–H systems. Adapted with permission from Ref. 10. Copyright © 2024 *Wiley-VCH GmbH*.

The same ACE-PBE-D3 potential has also been employed successfully in our previous investigations of hydriding-induced surface roughening and morphological evolution in Pd systems, further supporting its applicability to large-scale simulations of hydride-driven reconstruction processes.[2, 6, 7]

References

1. Schmidt, T.O. et al. Elucidation of Structure-Activity Relations in Proton Electroreduction at Pd Surfaces: Theoretical and Experimental Study. *Small* **18,** 2202410 (2022).
2. Ngoipala, A. et al. Hydride-Induced Reconstruction of Pd Electrode Surfaces: A Combined Computational and Experimental Study. *Adv Mater* **37,** 2410951 (2025).
3. Wang, V., Xu, N., Liu, J.-C., Tang, G. & Geng, W.-T. VASPKIT: A user-friendly interface facilitating high-throughput computing and analysis using VASP code. *Computer Physics Communications* **267**, 108033 (2021).
4. Qamar, M., Ngoipala, A., Mrovec, M., Vandichel, M. & Drautz, R. Hydride formation and phase separation in palladium nanoparticles from a transferable atomic cluster expansion potential. DOI:10.48550/arXiv.2606.09341, (2026).
5. Mrovec, M., Vandichel, M., Ngoipala, A., Qamar, M. & Drautz, R. ; Hydride formation and phase separation in palladium nanoparticles from a transferable atomic cluster expansion potential, Zenodo, DOI:10.5281/zenodo.20591787, 2026.
6. Viola, A. et al. Influence of the Size and Shape of Palladium Nanoparticles on Their Electrochemical Hydrogen Sorption Capacity. *J Am Chem Soc* **148**, 9548-9562 (2026).
7. Olson, K.J. et al. Kinetics of Hydrogen Absorption in Individual α-Phase Palladium Nanoparticles. *Acs Appl Energ Mater* **8**, 10256-10264 (2025).
