# Supplementary material for: Hydride-induced palladium self-diffusion and surface restructuring on Pd(111)
Source: Nat Commun. 2026 Sep 15;17:10839. doi: 10.1038/s41467-026-77704-9 (PMC13672345; doi:10.1038/s41467-026-77704-9)
Supplement: Supplementary file 2 [file 41467_2026_77704_MOESM2_ESM.pdf]

Description of Additional Supplementary Files

File Name: Supplementary Data 1

Description: Computational structures

Supplementary Data 1 contains the atomic structures used in the density functional theory and molecular dynamics calculations reported in this study. The files are provided as plain-text structural files and are organized into the following folders:

1. PdH_DFT_Models

This folder contains the optimized atomic coordinates (as CONTCAR files) of the PdH_x/Pd(111) models used in the density functional theory calculations with and without D3 corrections, including structures with different subsurface hydrogen concentrations, Pd-vacancy configurations and NEB trajectories.

2. PdH_MD_Models

This folder contains the initial and final atomic configurations of the PdH_x/Pd(111) systems used in the molecular dynamics simulations. The initial molecular dynamics configurations correspond to structures obtained after energy minimization. The final configurations correspond to the structures obtained after molecular dynamics equilibration.
