# Supplementary material for: Hydride-induced palladium self-diffusion and surface restructuring on Pd(111)
Source: Nat Commun. 2026 Sep 15;17:10839. doi: 10.1038/s41467-026-77704-9 (PMC13672345; doi:10.1038/s41467-026-77704-9)
Supplement: Supplementary file 3 [file 41467_2026_77704_MOESM4_ESM.pdf]

nature portfolio

# Peer Review File

# Hydride-Induced Palladium Self-Diffusion and Surface Restructuring on Pd(111)

Corresponding Author: Professor Matthias Vandichel

**This file contains all reviewer reports in order by version, followed by all author rebuttals in order by version.**

Version 0:

Reviewer comments:

Reviewer #1

(Remarks to the Author)
The authors have studied hydrogen-induced reconstruction of Pd surfaces through Pd atom mobility by means of DFT and MD simulations. The system was modeled as Pd (111) surfaces with 1-3 hydride layers. The results show enhanced Pd atom mobility towards the surface when vacancies are introduced in the surface layer. While providing insight into the behavior of these systems, the study design has significant shortcomings that limit the validity of the results. The work is therefore not recommended for publication in Nature Communications and instead suggested for a more specialized journal. Below are some comments that may help to improve the manuscript further:

1. Computational Methodology/DFT Models: Please describe the lattice constant of the slab models given the significant volume expansion due to hydrogen incorporation (DFT and MD models). This has important implications for how strained the different parts of the interface becomes. Moreover, it is mentioned that dislocations form in the MD models, which are not accounted for in the DFT models. Please comment.

2. The system is modeled with 3 fully hydrided surfaces layers. Given the fast diffusion of H in Pd and alpha-beta phase transition at significantly lower H content, this appears to be an artificial system. One could expect H to diffuse and distribute much further into the Pd lattice before allowing significant Pd atom diffusion.

3. Introduction: it is highlighted that hydriding of Pd leads to volume expansion that impacts catalytic performance. However, there are also significant changes to the electronic structure and vibrational properties, i.e., Pd and PdH are quite different materials.

4. Introduction: it is stated that Pd self-diffusion in hydrided lattices is underexplored while quite a few studies are cited. Curiously, Ref. 49 appears to be missing from the text, and this deals with exactly Pd self-diffusion in Pd–PdHx systems. The reasoning appears unnecessary – the authors could simply focus on the Pd self diffusion under initial hydriding conditions which is the focus and novelty of the study.

5. Title could be improved by including H or PdH. The use of unraveling is questionable, perhaps adjust the title to be more conclusive of the findings

Minor inputs:
The manuscript includes reminder notes of references to be included
Abstract: “upward” could be “outward” for clarity

Reviewer #2

(Remarks to the Author)
This is a very interesting manuscript by Nikkhah, Vandichel, and coworkers on surface reconstruction of Pd-based materials under cathodic reaction conditions. In the last two decades, idealized single-crystal models have been used to model catalytic processes under applied bias, although the harsh reaction conditions at the solid/ liquid interface often result in catalyst reconstruction. Little is known about reconstruction processes at the atomic scale, and the community is making efforts to comprehend surface reconstruction using a combination of DFT and MD simulations. This is also the focus of the

present work submitted to Nat Commun, which - in my opinion - is a very timely piece and of high relevance to the community. While Pd forms hydride phases under cathodic reaction conditions, the reconstruction process, the impact of hydrides, and Pd self-diffusion are not completely understood yet. Given that this article provides new insight into these phenomena, and the obtained results could also be transferred to other systems under cathodic polarization, I suggest this article for publication in Nat Commun, but ask the authors to perform a major revision prior to acceptance.

Major points:
- The flow in the main text is somewhat hampered because the authors often refer to their previous work. While the present article goes beyond the authors' previous works, it is a tedious task for the reader to check all the previous articles and fully understand the authors' discussion. I suggest that the authors extend the main text and make use of the word limit (max 5000 words) for an article. Currently, the submitted manuscript has rather the style of a communication. The presentation could also be further improved if the authors add additional figures, particularly if these figures show how they moved beyond their previous work (up to 10 figures for the main text). This will help to reduce the phrase "as shown in our previous article".
- The present article tackles surface reconstruction of a material with high relevance to electrocatalysis, considering that the reconstruction process takes place under cathodic polarization. In this sense, it might be an option to place your manuscript in the context of reconstruction and previous work in the community that gained insights into these phenomena for different systems using a combination of DFT and MD.
- The reported reconstruction of Pd takes places under cathodic polarization. Except for the calculated Pourbaix diagram in Figure 1, there is basically no information at which applied electrode potential the highlighted Pd self-diffusion takes place. I think this is one of the key information for the community to steer and control Pd reconstruction. While I do not believe that absolute values of the applied electrode potential make sense (or can be determined by ab initio theory with high accuracy), I still believe that the authors could comment on the potential range and provide additional analyses; for instance, by exploiting the work function of their surface slabs, which could be referenced to absolute value of the hydrogen electrode (about -4.60 V in VASP) to provide a potential range for Pd self-diffusion and Pd reconstruction.
- As far as I understand Figure 1a, the authors perform an analysis for a single vacancy, which is either located in the subsurface or at the surface. Why don't the authors look at two separate vacancies for the Pd(111) slab and compare the results to a single vacancy? This could provide information in how far the environment (no vacancy in the environment; vacancy in a distance of 1, 2, 3, ... atoms) modulates the Pd self-diffusion. I advise that at least the energetic picture for different reaction environments is quantified.

Minor points:
- Each figure needs to be accompanied with a single sentence indicating the main result of the plots displayed.
- Please check lines 140 and 142; obviously, the authors intended to add additional references.
- Lines 119/120: why do the authors omit vibrational and entropic contributions?
- The computational methodology section should provide information how the diffusivity coefficients were calculated based on the MD simulations.

Version 1:

Reviewer comments:

Reviewer #1

(Remarks to the Author)
The authors have addressed specific comments, resulting in an improved manuscript. However, the main concerns regarding novelty and significance, also in relation to the authors previous work, remain. Notably, the study lacks critical discussion of the main findings in relation to other literature, including experimental work. The manuscript is therefore recommended for a more specialized journal.

Reviewer #2

(Remarks to the Author)
The authors have made a thorough revision and have addressed all comments from the first review round. I fully support publication of their work in Nature Communications.

Reviewer #3

(Remarks to the Author)
This work combines DFT and machine-learning molecular dynamics simulations to investigate hydrogen-induced reconstruction and Pd atom mobility on Pd(111) surfaces. The key novelty lies in identifying the critical hydrogen loading required to activate Pd atom mobility. Regarding Reviewer #1's concern that a model with three fully hydrided surface layers is artificial because hydrogen would diffuse deeper into the Pd lattice before promoting Pd atom diffusion. The authors' response is well-reasoned and convincing. I agree with the authors' statement that "the pre-hydrided models serve as a controlled means of identifying the critical hydrogen loading at which Pd mobility is activated."
Therefore, I recommend the publication of this work after appropriate revisions based on the validation of ML-MD.
The authors employ ML-MD simulations based on an ACE-D3 type potential, yet throughout the manuscript (including the main text, Methods, and Supporting Information) there is no information on how this potential was trained, nor any quantification of its accuracy and uncertainties compared to reference DFT data. I would recommend that the authors include

the following information:
1. Details of the training dataset (e.g., number and types of configurations, range of compositions and geometries, sampling strategy);
2. Learning curves or error metrics (e.g., root-mean-square errors for energies and forces on training and test sets), at least in comparison with DFT calculations;
3. Validation of the potential against key structural and energetic properties of Pd–H systems (e.g., lattice constants, formation energies, diffusion barriers).
In addition, I would like the authors to comment on the fact that, under aqueous or electrochemical conditions, surface PdH can form naturally—as recently demonstrated, for example, where metal hydrides were identified as a new catalytically active phase at the solid–liquid interface (J. Catal. 2021, 404, 537–550). In this context, I believe the authors' findings in the present work are highly important.

Version 2:

Reviewer comments:

Reviewer #1

(Remarks to the Author)
The authors state that the discussion section has been substantially expanded to position the work within the broader literature. However, the discussing is still significantly lacking with respect to critical and quantitative comparisons of the obtained results with existing literature.

Most importantly, the novelty of the work is considered limited, in particular relative to the authors previous publications:

Hydride-Induced Reconstruction of Pd Electrode Surfaces
https://do.org/10.1002/adma.202410951

Computational surface Pourbaix diagrams to unravel cathodic hydride formation on defective palladium surfaces
(https://doi.org/10.1016/j.ijhydene.2024.02.248)

Computational unravelling of cathodic hydride formation on palladium surfaces
(https://doi.org/10.1016/j.ijhydene.2023.12.019)

Notably, the vacancy–hydrogen interactions which appear to be a significant driving force for the observed Pd atom mobility and restructuring is well-established, e.g.,

https://doi.org/10.1016/j.actamat.2022.118279
https://doi.org/10.1016/j.memsci.2024.123285

The work is therefore not considered to represent the novelty and quality required for publication in Nature Communications, and recommended for a more specialized journal.

Reviewer #3

(Remarks to the Author)
The authors have properply addressed my previous comments.

**Open Access** This Peer Review File is licensed under a Creative Commons Attribution 4.0 International License, which permits use, sharing, adaptation, distribution and reproduction in any medium or format, as long as you give appropriate credit to the original author(s) and the source, provide a link to the Creative Commons license, and indicate if changes were made.
In cases where reviewers are anonymous, credit should be given to 'Anonymous Referee' and the source.
The images or other third party material in this Peer Review File are included in the article's Creative Commons license, unless indicated otherwise in a credit line to the material. If material is not included in the article's Creative Commons license and your intended use is not permitted by statutory regulation or exceeds the permitted use, you will need to obtain permission directly from the copyright holder.
To view a copy of this license, visit https://creativecommons.org/licenses/by/4.0/

**RESPONSE TO REVIEWER COMMENTS**

Reviewer #1 (Remarks to the Author):

The authors have studied hydrogen-induced reconstruction of Pd surfaces through Pd atom mobility by means of DFT and MD simulations. The system was modeled as Pd (111) surfaces with 1-3 hydride layers. The results show enhanced Pd atom mobility towards the surface when vacancies are introduced in the surface layer. While providing insight into the behavior of these systems, the study design has significant shortcomings that limit the validity of the results. The work is therefore not recommended for publication in Nature Communications and instead suggested for a more specialized journal. Below are some comments that may help to improve the manuscript further:

1. Computational Methodology/DFT Models: Please describe the lattice constant of the slab models given the significant volume expansion due to hydrogen incorporation (DFT and MD models). This has important implications for how strained the different parts of the interface becomes. Moreover, it is mentioned that dislocations form in the MD models, which are not accounted for in the DFT models. Please comment.

**Response:** We thank the reviewer for raising this important point regarding lattice expansion, strain, and the consistency between the DFT and MD models.

The lattice constants for all $PdH_x$/Pd slab models are now explicitly reported in the revised manuscript (Methods section and Supporting Information). For the DFT calculations, all slab models were constructed from bulk Pd optimized at the PBE-D3 level after a thorough benchmark. The Pd(111) surface was cleaved from this optimized bulk structure, after which hydrogen was incrementally introduced into subsurface sites to generate increasing hydride loadings. Structural relaxation was performed using a constant volume optimization (ISIF = 4 scheme), allowing relaxation of atomic positions and the out-of-plane lattice parameter while keeping the in-plane lattice constants fixed to those of bulk Pd. Allowing full in-plane relaxation would instead correspond to a fully equilibrated bulk $PdH_x$ phase, whereas our approach is intended to model hydriding that initiates within an initially metallic Pd lattice under substrate constraint. This modelling choice reflects the physical assumption that hydriding initiates within an initially pure Pd lattice, with hydrogen incorporation occurring under the constraint of the underlying metallic framework, rather than starting from a pre-expanded bulk $PdH_x$ phase. As a result, hydride-induced strain accumulates locally in the

subsurface and surface regions, providing a controlled way to quantify how increasing hydrogen concentration modifies Pd migration barriers and local structural stability.

Regarding the formation of dislocations, we agree that these features are not observed in the DFT slab models. This is an inherent limitation of static, relatively small supercell DFT calculations, which are designed to capture local energetics and migration barriers under well-defined structural constraints. The system sizes accessible to DFT do not allow for the spontaneous nucleation and propagation of extended defects such as dislocations, nor for long-range strain relaxation processes. Importantly, the absence of dislocations in the DFT models does not compromise the validity of the calculated local migration barriers, which probe short-range atomic processes prior to large-scale plastic relaxation.

Importantly, this limitation directly motivated the use of large-scale molecular dynamics simulations with machine-learning potential (ACE-D3 type). The MD models explicitly allow for collective atomic motion, long-range strain redistribution, and defect formation over extended length and time scales. As a result, dislocations and surface reconstructions emerge naturally in the MD simulations once a critical subsurface hydride concentration is reached, providing a dynamic complement to the DFT results.

We now explicitly clarify this distinction in the manuscript, emphasizing that DFT is used to quantify thermodynamic stability and local migration barriers under controlled hydride loading, whereas ML-MD captures emergent atomistic reconstruction phenomena that are inaccessible to static DFT alone. Together, these approaches provide a consistent and multiscale description of hydride-induced Pd mobility.

"While these DFT calculations provide initial insights into the influence of hydride layers on Pd mobility, they only represent static, zero-temperature approximations and are restricted to relatively small supercells. As such, they do not capture the dynamical evolution of the surface over time, long-range strain redistribution, or the collective atomic rearrangements that accompany surface reconstruction during hydride-induced lattice relaxation. To bridge this gap, we employed ACE-PBE-D3-based MD simulations to explicitly track the time-dependent structural evolution of hydrided Pd surfaces at given temperature. This approach enables the exploration of strain relaxation mechanisms, defect formation, and collective atomic motion under varying hydrogen loadings. Given that the Pd(111) facet is the most stable and predominantly exposed surface in Pd nanostructure-based catalysts, we focused our MD investigations on $PdH_x$/Pd(111) systems with systematically varied subsurface hydride layers."

“For the DFT calculations, $PdH_x$/Pd(111) models were constructed by cleaving the optimized Pd bulk with a lattice constant of 3.891 Å (Figure S17). The 111 facet was chosen based on previous experimental and computational studies, where it was shown to be highly stable and commonly exposed over the synthesized nanoparticles.”

“The MD simulations were performed on a Pd(111) slab derived from the DFT-optimized bulk structure (a = 3.891 Å), consisting of 20 atomic layers with 576 Pd atoms per layer. For the initial $PdH_x$/Pd(111) configurations, the surface was fully covered with one monolayer of hydrogen atoms at three-fold fcc hollow sites, while subsurface hydrogen atoms were placed in octahedral interstitial sites.”

2. The system is modeled with 3 fully hydrided surfaces layers. Given the fast diffusion of H in Pd and alpha-beta phase transition at significantly lower H content, this appears to be an artificial system. One could expect H to diffuse and distribute much further into the Pd lattice before allowing significant Pd atom diffusion.

**Response:** We agree with the reviewer that the employed models represent an idealized description of the Pd–H system and do not aim to reproduce the entire chemical absorption or the electrochemical mechanism of proton reduction, including explicit solvent, applied potential, or hydrogen uptake kinetics. This choice is intentional and aligned with the primary objective of this study.

The focus of the present work is not the hydrogen uptake pathway itself, but rather the structural and mechanical response of Pd surfaces once a high local hydride concentration is established. To isolate this effect, we employ slab models in which hydrogen is pre-inserted into subsurface octahedral sites, thereby bypassing the initial adsorption and diffusion steps. This approach allows us to directly probe strain relaxation, Pd atom mobility, and surface reconstruction under β-$PdH_x$-like conditions. Accordingly, the modelled structures correspond to a post α→β phase transition regime rather than the dilute α-$PdH_x$ phase. Furthermore, in another (internationally collaborative) manuscript in preparation, the relevance of this type of system will be established for 25 nm nanoparticles, where it will be shown that, during absorption, the surface and subsurface will be populated first, followed by deeper subsurface layers.

Consistent with the reviewer’s expectation, our molecular dynamics simulations explicitly show that hydrogen atoms redistribute rapidly within the Pd lattice prior to any significant Pd atom migration. Pd diffusion and surface reconstruction occur only after sufficient local hydrogen accumulation, confirming that hydrogen mobility precedes and enables Pd atom

motion rather than occurring concurrently. The pre-hydrided models thus serve as a controlled means of identifying the critical hydrogen loading at which Pd mobility is activated.

A fully realistic description of hydrogen uptake under electrochemical conditions would require a grand-canonical framework that dynamically couples hydrogen chemical potential with atomic diffusion; such GC-MC/MD methodologies are currently under development by us, but are beyond the scope of the present study.

In the revised manuscript, we now clarify these modeling assumptions and their implications more explicitly and relate them directly to prior work employing similar hydride-preloaded Pd slab models.

"It is important to note that the pre-hydrided slab models employed here represent an idealized, hydrogen-rich regime corresponding to post α→β phase transition conditions rather than the dilute α-$PdH_x$ phase. These models are not intended to reproduce the full electrochemical hydrogen uptake pathway, including adsorption, diffusion into the bulk, and dynamic coupling to the applied potential. Instead, they are designed to isolate the structural and mechanical response of Pd surfaces once substantial subsurface hydrogen accumulation has been established. Consistent with physical expectations, our MD simulations show that hydrogen redistributes rapidly within the lattice prior to significant Pd atom migration, indicating that Pd mobility is enabled only after sufficient local hydride formation. Thus, the reported diffusivities and reconstruction behavior should be interpreted as characteristic of strongly hydrided, cathodic conditions where hydrogen-rich $PdH_x$ phases are thermodynamically stable, rather than as descriptors of early-stage dilute hydrogen incorporation."

3. Introduction: it is highlighted that hydriding of Pd leads to volume expansion that impacts catalytic performance. However, there are also significant changes to the electronic structure and vibrational properties, i.e., Pd and PdH are quite different materials.

**Response:** We thank the reviewer for this important clarification. We fully agree that hydriding of Pd not only induces volumetric expansion and lattice strain but also leads to substantial changes in electronic structure and vibrational properties. We have therefore revised the Introduction to explicitly note this.

"This phase transition is accompanied not only by a substantial volumetric expansion (≈10%) that generates residual stresses and lattice distortions, but also by pronounced changes in the

electronic structure and vibrational properties of the material, such that Pd and $PdH_x$ exhibit distinct physicochemical characteristics.[6-10]"

4. Introduction: it is stated that Pd self-diffusion in hydrided lattices is underexplored while quite a few studies are cited. Curiously, Ref. 49 appears to be missing from the text, and this deals with exactly Pd self-diffusion in Pd–PdHx systems. The reasoning appears unnecessary – the authors could simply focus on the Pd self diffusion under initial hydriding conditions which is the focus and novelty of the study.

**Response:** We thank the reviewer for pointing this out. We acknowledge that Ref. 49, which investigates Pd self-diffusion in Pd–$PdH_x$ systems, was inadvertently not cited in the original version of the manuscript. This omission was an oversight, and we have now corrected it by explicitly citing and discussing Ref. 49 in the revised Introduction.

We note, however, that the focus of Ref. 49 is on bulk Pd–$PdH_x$ models constructed as special quasirandom structures at fixed hydrogen concentrations ($x = 0.25, 0.5, 0.75$), and the analysis is performed in a bulk context. In contrast, the present work addresses Pd self-diffusion from a surface- and interface-driven perspective, specifically during the early stages of hydriding where hydride formation, strain accumulation, and surface reconstruction are strongly coupled. Our study therefore targets a distinct physical regime in which Pd atom mobility is activated locally at hydrided surfaces and near-surface interfaces, rather than describing steady-state diffusion in fully equilibrated bulk $PdH_x$ phases. These surface-mediated processes and their role in enabling Pd atom mobility are not addressed in bulk Pd–$PdH_x$ diffusion studies.

We agree with the reviewer that emphasizing the specific regime studied here, Pd self-diffusion under initial hydriding and surface reconstruction conditions, provides a clearer and more accurate framing of the novelty. Accordingly, we have revised the Introduction to explicitly acknowledge prior bulk diffusion studies, including Ref. 49 (**now Ref. 44**), and have streamlined the motivation to focus directly on Pd self-diffusion during the onset of hydriding at Pd surfaces, without relying on broader claims of under exploration.

"While numerous studies have examined hydrogen absorption and diffusion in Pd (surfaces slabs and nanoparticles),[25-39] and Pd-self diffusion in bulk Pd-$PdH_x$ systems[40], considerably less attention has been paid to Pd atom mobility at Pd surfaces during the initial stages of hydride formation, where local hydrogen accumulation, strain, and surface reconstruction are strongly coupled.[14]"

5. Title could be improved by including H or PdH. The use of unraveling is questionable, perhaps adjust the title to be more conclusive of the findings.

**Response:** We thank the reviewer for this suggestion and now updated the title as: "Hydride-Induced Palladium Self-Diffusion and Surface Restructuring on Pd(111)

Minor inputs:

The manuscript includes reminder notes of references to be included

**Response:** All corresponding references have been properly added and formatted in the revised manuscript.

Abstract: "upward" could be "outward" for clarity

**Response:** We have implemented this in the revised manuscript.

Reviewer #2 (Remarks to the Author):

This is a very interesting manuscript by Nikkhah, Vandichel, and coworkers on surface reconstruction of Pd-based materials under cathodic reaction conditions. In the last two decades, idealized single-crystal models have been used to model catalytic processes under applied bias, although the harsh reaction conditions at the solid/ liquid interface often result in catalyst reconstruction. Little is known about reconstruction processes at the atomic scale, and the community is making efforts to comprehend surface reconstruction using a combination of DFT and MD simulations. This is also the focus of the present work submitted to Nat Commun, which - in my opinion - is a very timely piece and of high relevance to the community. While Pd forms hydride phases under cathodic reaction conditions, the reconstruction process, the impact of hydrides, and Pd self-diffusion are not completely understood yet. Given that this article provides new insight into these phenomena, and the obtained results could also be transferred to other systems under cathodic polarization, I suggest this article for publication in Nat Commun, but ask the authors to perform a major revision prior to acceptance.

Major points:

The flow in the main text is somewhat hampered because the authors often refer to their previous work. While the present article goes beyond the authors' previous works, it is a tedious task for the reader to check all the previous articles and fully understand the authors' discussion. I suggest that the authors extend the main text and make use of the word limit (max 5000 words) for an article. Currently, the submitted manuscript has rather the style of a communication. The presentation could also be further improved if the authors add additional figures, particularly if these figures show how they moved beyond their previous work (up to 10 figures for the main text). This will help to reduce the phrase "as shown in our previous article".

**Response:** We thank the reviewer for this constructive suggestion and fully agree with this assessment. In the revised manuscript, we have substantially expanded the main text to improve continuity and readability, making fuller use of the permitted word limit. Where possible, key concepts, assumptions, and results that were previously referred are now explicitly described and discussed within the present manuscript, thereby rendering the manuscript more self-contained and reducing the need for the reader to consult earlier publications.

In addition, we have revised the presentation to more clearly highlight the advances beyond our prior studies. New figures have been added to the main text to directly illustrate the progression from earlier work to the current results, particularly with respect to hydride-induced Pd atom mobility, strain relaxation, and surface reconstruction. These additions reduce reliance on external references and provide a more self-contained and coherent narrative.

- The present article tackles surface reconstruction of a material with high relevance to electrocatalysis, considering that the reconstruction process takes place under cathodic polarization. In this sense, it might be an option to place your manuscript in the context of reconstruction and previous work in the community that gained insights into these phenomena for different systems using a combination of DFT and MD.

**Response:** We thank the reviewer for this suggestion and agree. In the revised manuscript, we have expanded the Introduction and Discussion to better place our results within the broader context of surface reconstruction under cathodic polarization, drawing clearer connections to previous DFT and MD studies on related catalytic systems.

"Beyond Pd systems, hydrogen-induced surface reconstruction under electrocatalytic conditions has been investigated in other transition metals using advanced theoretical approaches. For example, grand-canonical DFT (GC-DFT) combined with global optimization strategies have revealed hydrogen-driven restructuring phenomena on Cu surfaces under reducing potentials.[23,24] These studies demonstrate that adsorbate-induced stabilization of metastable surface motifs can significantly alter catalytic behavior. More recently, Alexandrova and co-workers developed a quasi-kinetic Monte Carlo framework capable of capturing potential-dependent surface evolution beyond equilibrium Boltzmann statistics, explicitly accounting for kinetic trapping under reducing and elevated-temperature conditions.[25, 26] Collectively, these advances underscore the dynamic nature of catalyst surfaces under reaction environments and emphasize the need for multiscale approaches that couple thermodynamics with atomic-scale kinetics."

"It is important to note that the pre-hydrided slab models employed here represent an idealized, hydrogen-rich regime corresponding to post α à β phase transition conditions rather than the dilute α-$PdH_x$ phase. These models are not intended to reproduce the full electrochemical hydrogen uptake pathway, including adsorption, diffusion into the bulk, and dynamic coupling to the applied potential. Instead, they are designed to isolate the structural and mechanical response of Pd surfaces once substantial subsurface hydrogen accumulation has been

established. Consistent with physical expectations, our MD simulations show that hydrogen redistributes rapidly within the lattice prior to significant Pd atom migration, indicating that Pd mobility is enabled only after sufficient local hydride formation. Thus, the reported diffusivities and reconstruction behavior should be interpreted as characteristic of strongly hydrided, cathodic conditions where hydrogen-rich $PdH_x$ phases are thermodynamically stable, rather than as descriptors of early-stage dilute hydrogen incorporation."

- The reported reconstruction of Pd takes places under cathodic polarization. Except for the calculated Pourbaix diagram in Figure 1, there is basically no information at which applied electrode potential the highlighted Pd self-diffusion takes place. I think this is one of the key information for the community to steer and control Pd reconstruction. While I do not believe that absolute values of the applied electrode potential make sense (or can be determined by ab initio theory with high accuracy), I still believe that the authors could comment on the potential range and provide additional analyses; for instance, by exploiting the work function of their surface slabs, which could be referenced to absolute value of the hydrogen electrode (about -4.60 V in VASP) to provide a potential range for Pd self-diffusion and Pd reconstruction.

**Response:** We thank the reviewer for this important comment and agree that clarifying the electrochemical potential window associated with Pd self-diffusion and reconstruction is valuable for the community.

In the present work, the applied potential enters explicitly through the surface Pourbaix analysis (Figure 1), which defines the thermodynamic stability of the hydrided $PdH_x$/Pd(111) states as a function of electrode potential. Our SPDs show that hydrogen-rich $PdH_x$ phases become thermodynamically stable under cathodic conditions, including at and below $U_{SHE} \approx 0$ V, indicating that subsurface hydride formation is highly favorable in reducing environments. The DFT migration barriers and subsequent MD simulations were then performed for these thermodynamically stable hydrided configurations.

Importantly, Pd self-diffusion is not directly driven by the applied potential but rather by hydride-induced strain and lattice softening. The applied potential controls the hydrogen chemical potential, which governs subsurface hydrogen accumulation; once a critical hydride loading is reached (corresponding to approximately three hydride sublayers in our simulations), Pd migration and surface reconstruction become activated. In this sense, Pd self-diffusion is indirectly potential controlled via hydride stability rather than occurring at a uniquely defined electrode potential.

To provide an approximate electrochemical potential window for Pd self-diffusion/reconstruction, we evaluated the work functions ($\Phi$) of representative slab configurations along the diffusion event (initial and final states) under different subsurface H loadings. Here, the initial state corresponds to a slab with a surface vacancy, whereas the final state corresponds to a configuration where the vacancy is relocated into the subsurface, i.e., a more "healed/ironed" surface. Since $\Phi = V_{\text{vac}} - E_F$ is highly sensitive to the surface dipole, this comparison provides a physical descriptor for how the surface changes during reconstruction.

Across the tested hydrogen loadings, $\Phi$ remains within a relatively narrow range indicating that Pd migration proceeds without drastic changes in the slab's electronic surface reference. Subsurface hydrogen generally reduces the $\Phi$ difference between the initial and final configurations for a single vacancy; however, for double vacancies, we do not observe any correlation. We note that mapping $\Phi$ to an absolute potential scale via $U_{\text{SHE}} \approx \Phi - 4.60\text{V}$ should be interpreted cautiously for neutral-slab calculations (no explicit double layer/solvent), and we therefore emphasize the relative trends rather than absolute potentials.

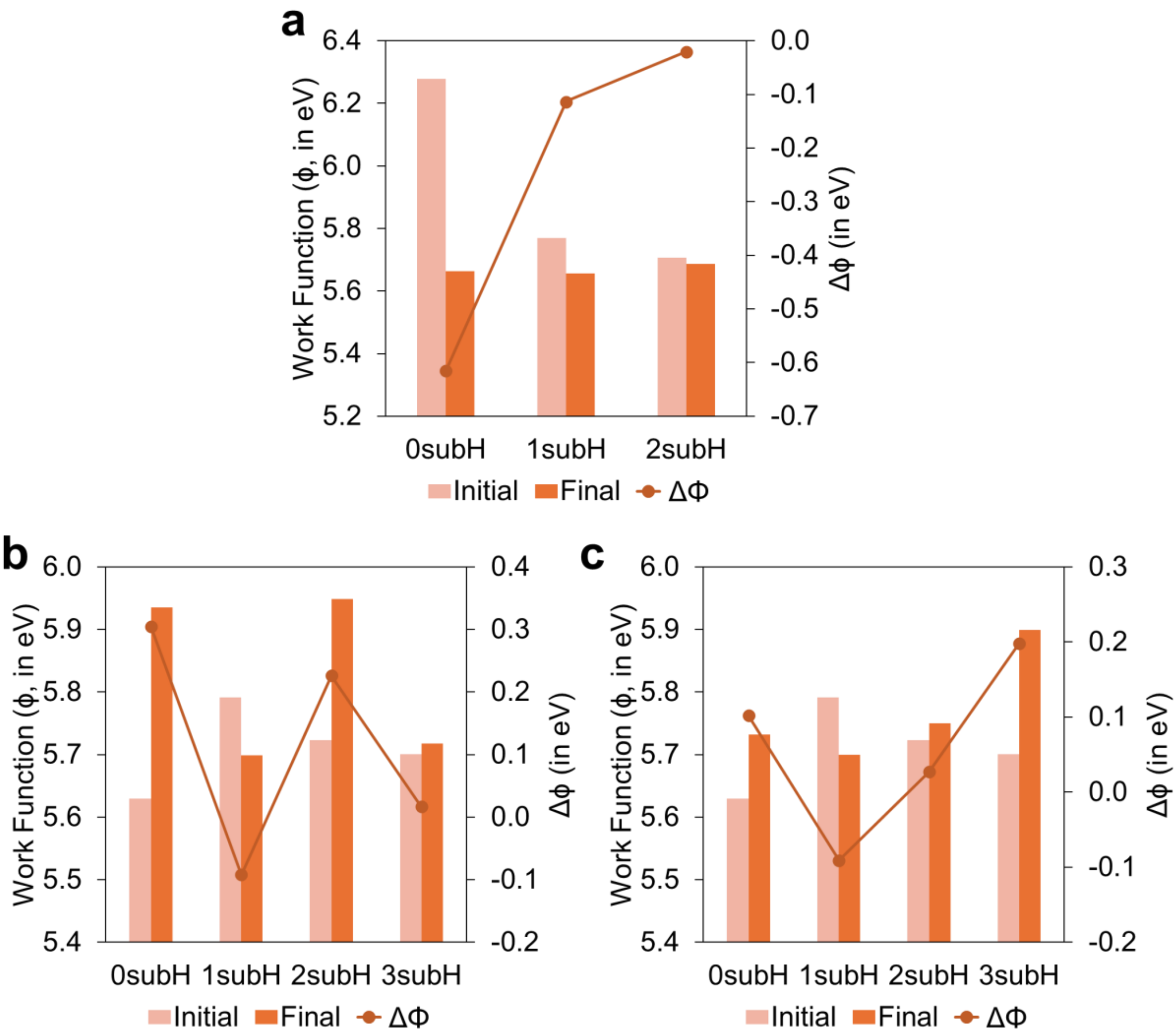

**Figure 1 (response to reviewers).** Work functions (Φ, eV) for the configurations along vacancy-mediated Pd self-diffusion pathways under different subsurface hydrogen loadings with (a) single vacancy and (b) double vacancies via route 1 (R1) and (c) double vacancies via route 2 (R2). See **Figure 2 (response to reviewers)** below for the pathways R1 and R2.

We have now clarified this connection explicitly in the manuscript, emphasizing that reconstruction is expected within the cathodic potential regime where hydrogen-rich $PdH_x$ phases are thermodynamically stable, as identified by the surface Pourbaix analysis.

"Importantly, since the hydrogen-rich $PdH_x$ states investigated in the subsequent DFT and MD analyses are thermodynamically stable within this cathodic potential regime, Pd self-diffusion and surface reconstruction should be interpreted as occurring under reducing conditions where sufficient subsurface hydrogen accumulation is achieved. In this sense, the applied potential indirectly governs Pd mobility through control of the hydrogen chemical potential and hydride loading, rather than directly modifying the migration barrier."

"In addition, we evaluated the work functions (Φ) of representative slab configurations along the vacancy-mediated diffusion coordinate to provide an approximate electrostatic reference for the potential window associated with Pd migration. The calculated work functions vary only little along the migration pathway, indicating that Pd self-diffusion proceeds without substantial changes in the electronic structure. In the single-vacancy models, subsurface hydrogen loading reduces the Φ difference between the initial (surface vacancy) and final (subsurface vacancy) states, consistent with hydrogen stabilizing reconstructed configurations. (Table S1). However, in the case of double vacancies, the work function variation does not show any correlations."

- As far as I understand Figure 1a, the authors perform an analysis for a single vacancy, which is either located in the subsurface or at the surface. Why don't the authors look at two separate vacancies for the Pd(111) slab and compare the results to a single vacancy? This could provide information in how far the environment (no vacancy in the environment; vacancy in a distance of 1, 2, 3, ... atoms) modulates the Pd self-diffusion. I advise that at least the energetic picture for different reaction environments is quantified.

**Response:** We thank the reviewer for this insightful suggestion and agree that the local vacancy environment can significantly influence Pd self-diffusion. In response, we have performed

additional calculations explicitly considering configurations with two vacancies in the Pd(111) slab at different relative separations.

The new results are presented in the revised main text (Fig. 2) and discussed in detail, providing a more comprehensive energetic picture of Pd self-diffusion across different reaction environments, as suggested by the reviewer. We believe this additional analysis significantly strengthens the mechanistic interpretation of hydride-induced Pd migration.

"To further evaluate the influence of the local vacancies on Pd self-diffusion, we extended our analysis to configurations containing two vacancies in the Pd(111) slab at different relative positions. This allows assessment of how neighbouring defects modulate migration energetics compared to the isolated single-vacancy case. Two distinct diffusion pathways were examined. In Route 1, a Pd atom from the second layer (L2) first occupies the adjacent L2 vacancy ($\mathbf{V'_{L2}}$) through a lateral in-layer hop, followed by an upward migration into the surface vacancy ($\mathbf{V_{L1}}$). In Route 2, a Pd atom from the third layer (L3) first fills the $\mathbf{V_{L2}}$, after which the atom migrates upward into the surface vacancy. Thus, Route 1 proceeds via lateral rearrangement before vertical migration, whereas Route 2 involves a deeper-layer exchange mechanism. The calculated energetics indicate that, although the detailed barriers differ between the two routes, the qualitative trend remains consistent with the single-vacancy model: increasing subsurface hydrogen loading lowers the migration barrier and stabilizes the reconstructed configuration. In other words, hydride formation facilitates Pd self-diffusion irrespective of whether the vacancy is isolated or part of a defect cluster. This suggests that hydrogen-rich environments promote defect-assisted surface restructuring even in more complex vacancy landscapes.

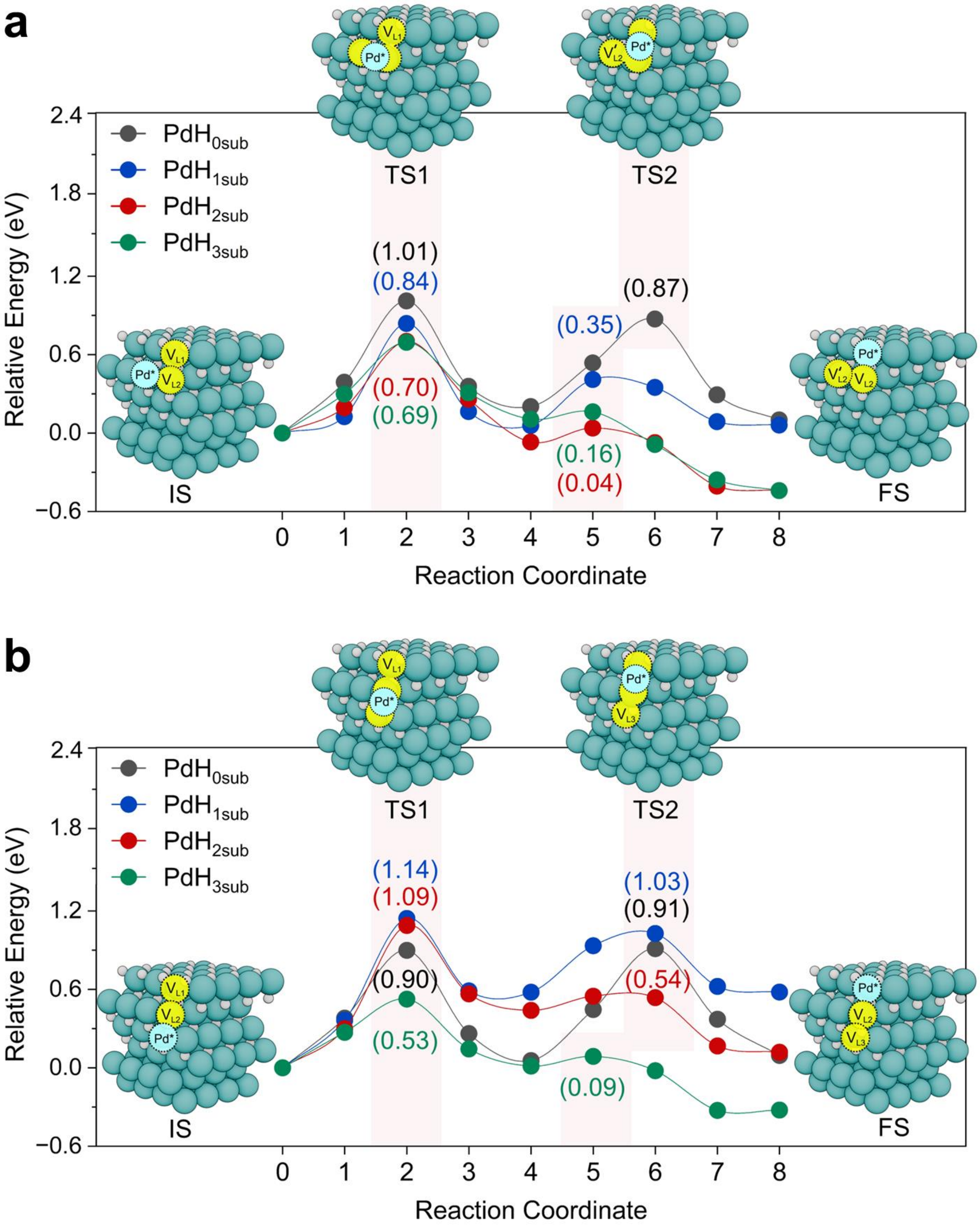


**Figure 2 (response to reviewers).** Reaction energy profiles for Pd migration on Pd(111) surfaces with a double-vacancy configuration, showing the relative energies of the initial, intermediate, and final states, along with the corresponding migration barriers for different subsurface hydrogen concentrations ($PdH_{0sub}$, $PdH_{1sub}$, $PdH_{2sub}$, $PdH_{3sub}$). Two distinct pathways are considered: (a) Route 1 (R1) involves lateral migration within the second layer (L2) followed by upward diffusion into the surface vacancy (L1), while (b) Route 2 (R2) proceeds via a deeper-layer exchange mechanism (L3 → L2 → L1)."

Minor points:

- Each figure needs to be accompanied with a single sentence indicating the main result of the plots displayed.

**Response:** We thank the reviewer for this suggestion. All figure captions have now been revised to include a concise summary sentence highlighting the main result conveyed by each figure.

- Please check lines 140 and 142; obviously, the authors intended to add additional references.

**Response:** All corresponding references have been properly added and formatted in the revised manuscript.

- Lines 119/120: why do the authors omit vibrational and entropic contributions?

**Response:** We thank the reviewer for raising this point and appreciate the opportunity to clarify the distinction between the thermodynamic analysis and the migration barrier calculations.

The omission of vibrational and entropic contributions applies specifically to the Pd migration barrier analysis, which is based on minimum-energy pathways obtained from nudged elastic band (NEB) calculations. In line with common practice for solid-state diffusion studies, the migration barriers are reported as potential energy differences ($\Delta E$) between the initial state and the saddle point, rather than free energy barriers ($\Delta G^{\ddagger}$). For atomic diffusion in crystalline solids, entropic and vibrational contributions to the barrier are typically small compared to the migration energy and often partially cancel between the initial and transition states, particularly when comparing *relative* trends across similar environments.

We note that vibrational and entropic contributions are explicitly considered where appropriate, namely in the construction of the surface Pourbaix diagrams, which are based on thermodynamic free energies.

- The computational methodology section should provide information on how the diffusivity coefficients were calculated based on the MD simulations.

**Response:** The methodology used to calculate the diffusivity coefficients from the MD simulations was already provided in the Supporting Information. To improve clarity and accessibility, we have now expanded this description and added a summary in the Computational Methodology section of the main manuscript, with explicit reference to the detailed procedure in the Supporting Information.

**RESPONSE TO REVIEWER COMMENTS**

Reviewer #1 (Remarks to the Author):

The authors have addressed specific comments, resulting in an improved manuscript. However, the main concerns regarding novelty and significance, also in relation to the authors previous work, remain. Notably, the study lacks critical discussion of the main findings in relation to other literature, including experimental work. The manuscript is therefore recommended for a more specialized journal.

**Response**: We thank the reviewer for this comment and appreciate the continued assessment of the manuscript. In response to the concerns raised during the first round of review, we substantially expanded both the Introduction and Discussion sections to better position the present work within the broader literature on catalyst reconstruction and dynamic surface evolution under reaction conditions.

Specifically, we now discuss experimental studies demonstrating hydriding-induced defect formation, strain accumulation, and surface reconstruction in Pd materials, including coherent X-ray diffraction and electrochemical investigations, alongside recent theoretical advances employing GC-DFT, global optimization approaches, and quasi-kinetic Monte Carlo simulations to describe adsorbate-induced restructuring in catalytic systems. We also provide a more comprehensive comparison with prior studies of Pd self-diffusion, vacancy migration, and hydrided Pd systems.

While hydriding-induced reconstruction has been observed experimentally, obtaining atomistic insight into the underlying mechanisms remains challenging because hydrogen is difficult to probe directly under operando conditions and because the associated structural transformations occur dynamically across multiple length and time scales. In this context, the combination of DFT, surface Pourbaix analysis, and large-scale molecular dynamics employed here provides mechanistic insight into how hydride accumulation, strain development, and Pd self-diffusion collectively drive surface restructuring.

The revised manuscript now provides a substantially broader discussion of the existing literature and experimental observations, while highlighting the distinct contribution of the present work: establishing the thermodynamic and kinetic conditions under which hydride-induced Pd atom mobility and surface reconstruction become activated.

Reviewer #2 (Remarks to the Author):

The authors have made a thorough revision and have addressed all comments from the first review round. I fully support publication of their work in Nature Communications.

**Response:** We thank the reviewer for the careful evaluation of our manuscript and for the positive assessment of the revised version. We appreciate the reviewer's constructive comments throughout the review process, which have helped improve the clarity, scope, and presentation of the work.

Reviewer #3 (Remarks to the Author):

This work combines DFT and machine-learning molecular dynamics simulations to investigate hydrogen-induced reconstruction and Pd atom mobility on Pd(111) surfaces. The key novelty lies in identifying the critical hydrogen loading required to activate Pd atom mobility. Regarding Reviewer #1's concern that a model with three fully hydrided surface layers is artificial because hydrogen would diffuse deeper into the Pd lattice before promoting Pd atom diffusion. The authors' response is well-reasoned and convincing. I agree with the authors' statement that "the pre-hydrided models serve as a controlled means of identifying the critical hydrogen loading at which Pd mobility is activated." Therefore, I recommend the publication of this work after appropriate revisions based on the validation of ML-MD. The authors employ ML-MD simulations based on an ACE-D3 type potential, yet throughout the manuscript (including the main text, Methods, and Supporting Information) there is no information on how this potential was trained, nor any quantification of its accuracy and uncertainties compared to reference DFT data.

I would recommend that the authors include the following information:

1. Details of the training dataset (e.g., number and types of configurations, range of compositions and geometries, sampling strategy);

2. Learning curves or error metrics (e.g., root-mean-square errors for energies and forces on training and test sets), at least in comparison with DFT calculations;

3. Validation of the potential against key structural and energetic properties of Pd–H systems (e.g., lattice constants, formation energies, diffusion barriers).

**Response:** We thank the reviewer for this important comment and agree that the reliability of the ACE-PBE-D3 potential should be documented more clearly in the manuscript.

The primary objective of the present work is to investigate hydride-induced Pd mobility and surface reconstruction rather than to introduce a new machine-learning interatomic potential. Consequently, a detailed description of the ACE-PBE-D3 development and training procedure was not included in the original manuscript. However, we agree that sufficient validation information should be provided to allow readers to assess the reliability of the MD results.

In the revised manuscript and Supporting Information, we have therefore added a summary of the ACE-PBE-D3 training and validation. Specifically, we now provide: (i) a concise description of the training dataset and its coverage of Pd–H configurations, (ii) quantitative error metrics for energies and forces relative to the reference DFT dataset, and (iii) validation against key structural, elastic, and vibrational properties of Pd and PdH, including equilibrium lattice parameters, bulk moduli, energy–volume curves, and phonon dispersions.

In addition, we now explicitly reference our previous studies that employed the same ACE-PBE-D3 potential and demonstrated its ability to accurately reproduce the fundamental bulk and surface properties of Pd and PdH systems relevant to hydriding and surface reconstruction. For readers interested in the complete development, training strategy, and extensive benchmarking of the ACE-PBE-D3 potential, a detailed description is now also submitted to Nature Communication (**NCOMMS-26-045161)**; which forms the basis of a separate methodological study currently under consideration for publication and its preprint and data are now available (https://arxiv.org/abs/2606.09341, https://zenodo.org/records/20591788). We believe that the additional validation data provided in the revised Supporting Information, together with the cited methodological references, sufficiently demonstrate the accuracy and transferability of the ACE-PBE-D3 potential for the hydride-induced reconstruction phenomena investigated in the present work.

**The following text was added to the Supporting Information:**

**"Validation of the Atomic Cluster Expansion (ACE-PBE-D3) Potential**

The large-scale molecular dynamics simulations presented in this work were performed using an Atomic Cluster Expansion potential trained on a PBE-D3 reference dataset (ACE-PBE-D3). A comprehensive description of the potential development, training procedure, and

benchmarking is available as a preprint[1] and in data repository[2]. Here, we summarize the key validation results most relevant to the present investigation of hydride-induced Pd reconstruction.

**Training Dataset**

The ACE-PBE-D3 potential was trained using a diverse dataset of Pd–H configurations generated from density functional theory calculations. The dataset includes bulk Pd and PdH structures spanning a wide range of hydrogen concentrations, surface slabs, strained configurations, thermally perturbed structures, and hydrogen-containing environments relevant to hydriding and surface reconstruction. To ensure robust transferability, both low-energy equilibrium structures and higher-energy distorted configurations were included.

All structures within 2 eV atom$^{-1}$ above the ground-state convex hull represent approximately 99% of the reference dataset, while a smaller subset of higher-energy configurations was retained to improve the description of short-range repulsive interactions.

**Energy and Force Accuracy**

The final ACE-PBE-D3 model achieves an energy root-mean-square error (RMSE) of 9 meV atom$^{-1}$ for low-energy structures and 39 meV atom$^{-1}$ across the complete dataset. Corresponding force RMSE values are 65 meV Å$^{-1}$ for low-energy structures and 97 meV Å$^{-1}$ for the full dataset. A 10% hold-out test set, excluded entirely from training, was used for validation. **Figure S20 (a-b)** shows cross-validation plots for predicted energies and forces, respectively, demonstrating excellent agreement between ACE predictions and reference DFT calculations.

**Structural, Elastic and Vibrational Properties**

To assess the transferability of the potential, equilibrium properties of Pd and PdH crystals were compared against DFT calculations. **Figure S20c** shows the energy–volume curves for fcc-Pd, $PdH_{0.5}$, and stoichiometric PdH obtained using ACE and DFT. Excellent agreement is observed across the entire volume range considered. **Figure S20d** compares the equilibrium lattice constants and bulk moduli predicted by ACE with DFT and available experimental measurements. The close agreement confirms that the potential accurately reproduces the structural response of Pd and PdH phases, which is particularly important for describing hydride-induced lattice expansion and strain accumulation. Because lattice dynamics play an important role in hydriding phenomena, phonon dispersions were also evaluated. **Figure S20e,f**

compares phonon band structures obtained using ACE and DFT for fcc-Pd and stoichiometric PdH, respectively. The ACE potential accurately reproduces the principal vibrational features of both systems and captures the essential phonon characteristics associated with hydrogen incorporation.

The present work focuses on hydride-induced Pd migration, strain relaxation, and surface reconstruction. These phenomena depend critically on the accurate description of:

(i) Pd–Pd and Pd–H interactions,

(ii) lattice expansion associated with hydriding,

(iii) elastic response and strain accumulation, and

(iv) finite-temperature atomic dynamics.

The excellent agreement between ACE and DFT for structural, elastic, energetic, and vibrational properties provides confidence that the ACE-PBE-D3 potential is sufficiently accurate and transferable for investigating the hydrided $PdH_x$/Pd(111) systems considered in this study.

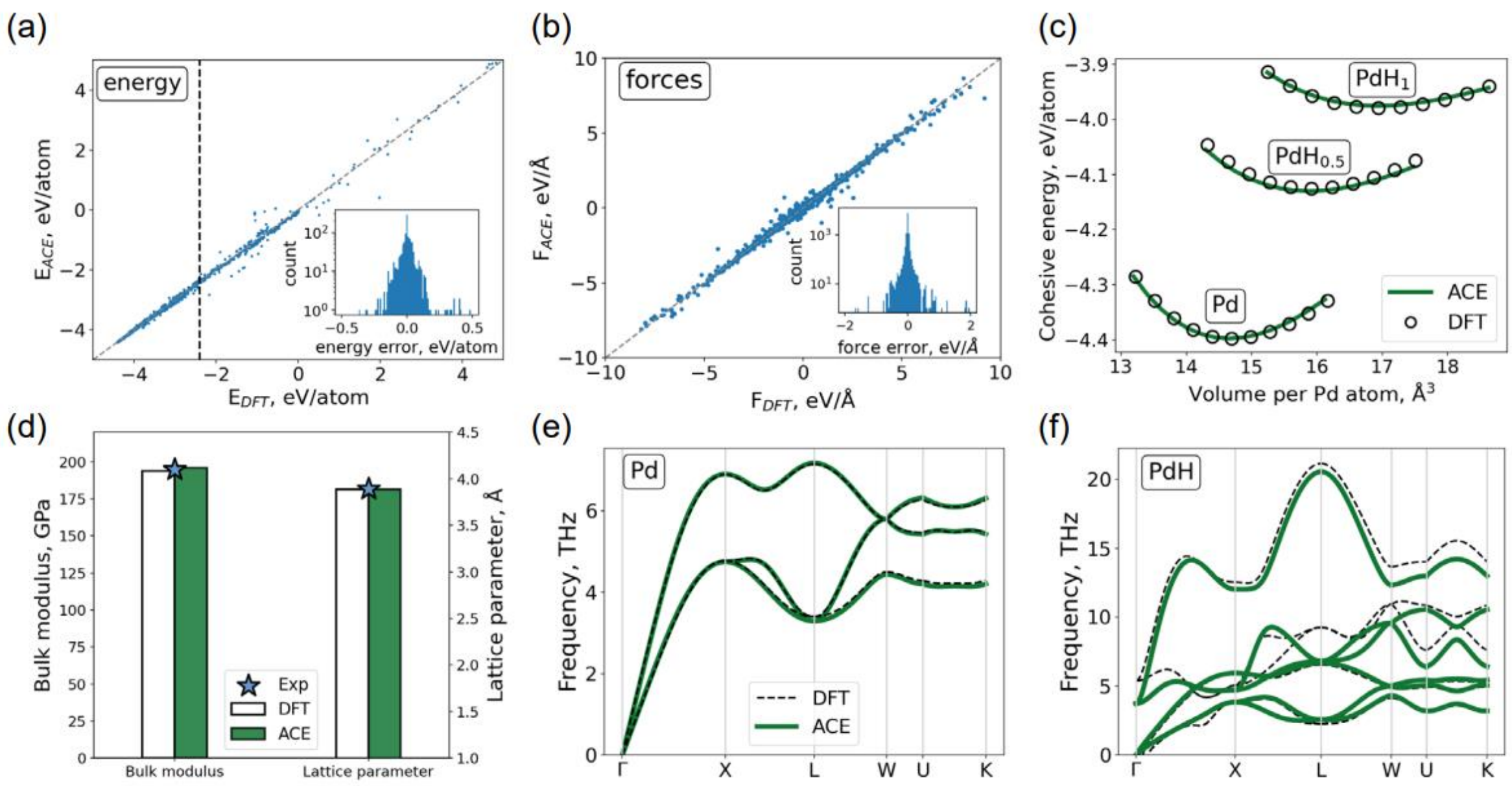


**Figure S20. Validation of the ACE-PBE-D3 potential.** (a) Cross-validation parity plot for predicted energies relative to reference DFT calculations, with the inset showing the corresponding error distribution. The vertical dashed line denotes the energy cutoff below which approximately 99% of the structures in the dataset are located. (b) Cross-validation parity plot for predicted forces, with the inset showing the force error distribution. (c) Energy–volume curves of fcc-Pd, $PdH_{0.5}$, and stoichiometric PdH predicted by ACE-PBE-D3 and DFT, demonstrating excellent agreement across a range of lattice volumes. (d) Comparison of

equilibrium lattice constants and bulk moduli obtained using ACE-PBE-D3, DFT, and available experimental data, confirming accurate reproduction of the structural and elastic properties of Pd and PdH phases. (e,f) Phonon band structures of fcc-Pd and stoichiometric PdH, respectively, calculated using ACE-PBE-D3 and DFT, showing that the potential accurately captures the vibrational properties relevant to hydriding phenomena. Collectively, these results demonstrate the accuracy and transferability of the ACE-PBE-D3 potential for describing the structural, elastic, energetic, and vibrational properties of Pd–H systems. Adapted with permission from Ref. 10. Copyright © 2024 *Wiley-VCH GmbH*.

The same ACE-PBE-D3 potential has also been employed successfully in our previous investigations of hydriding-induced surface roughening and morphological evolution in Pd systems, further supporting its applicability to large-scale simulations of hydride-driven reconstruction processes.[3-5]"

**Additional Comment:** In addition, I would like the authors to comment on the fact that, under aqueous or electrochemical conditions, surface PdH can form naturally—as recently demonstrated, for example, where metal hydrides were identified as a new catalytically active phase at the solid–liquid interface (J. Catal. 2021, 404, 537–550). In this context, I believe the authors' findings in the present work are highly important.

**Response:** We thank the reviewer for this valuable suggestion and fully agree that the growing recognition of surface and near-surface metal hydrides under aqueous and electrochemical conditions further strengthens the relevance of the present study. We have now expanded the Discussion to include recent experimental evidence demonstrating the formation of catalytically active hydride phases at solid–liquid interfaces.

"Beyond their traditional role in hydrogen storage, hydride phases are increasingly being recognized as catalytically relevant species under aqueous and electrochemical conditions.[6-8] For example, Xia and Wang demonstrated that interfacial water dissociation at Pd-supported catalysts can generate Pd hydrides that act as a new catalytically active phase at the solid–liquid interface, directly participating in catalytic turnover rather than merely serving as hydrogen reservoirs.[9] Such findings suggest that hydride formation can fundamentally alter both the catalytic and structural state of Pd under operating conditions. Consequently, understanding how hydriding influences not only reactivity but also atomic mobility, defect evolution, and surface reconstruction is essential for establishing realistic structure–property relationships in Pd-based catalysts."
